# Recent advances in the chemistry of 2-chloroquinoline-3-carbaldehyde and related analogs

**DOI:** 10.1039/c7ra11537g

**Published:** 2018-02-23

**Authors:** Wafaa S. Hamama, Mona E. Ibrahim, Ayaa A. Gooda, Hanafi H. Zoorob

**Affiliations:** Department of Chemistry, Faculty of Science, Mansoura University El-Gomhoria Street Mansoura 35516 Egypt wshamama53@gmail.com wshamama@yahoo.com +2050 2246254 +2050 2242388

## Abstract

This review highlights the recently cited research data in the literature on the chemistry of 2-chloroquinoline-3-carbaldehyde and related analogs and their applications over the period from 2013 to 2017. It covers: synthesis of quinoline ring systems and reactions adopted to construct fused or binary quinoline-cord heterocyclic systems. The biological evaluation and the synthetic applications of the target compounds were illustrated.

## Abstract 2

This review describes the recent publications reported on the chemistry of 2-chloroquinoline-3-carbaldehydes. Heterocyclic quinoline ring systems are binary and fused cycles.

## Graphical abstract

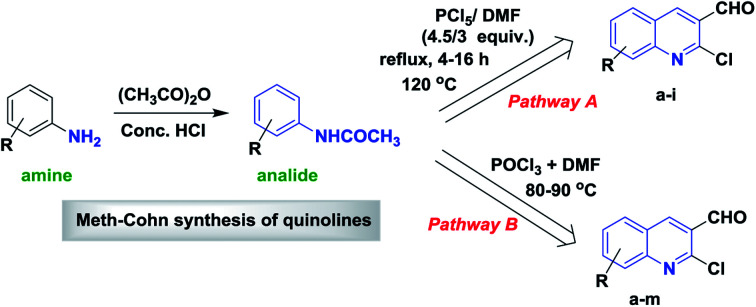

## 1. Introduction and scope

**Fig. 1:**
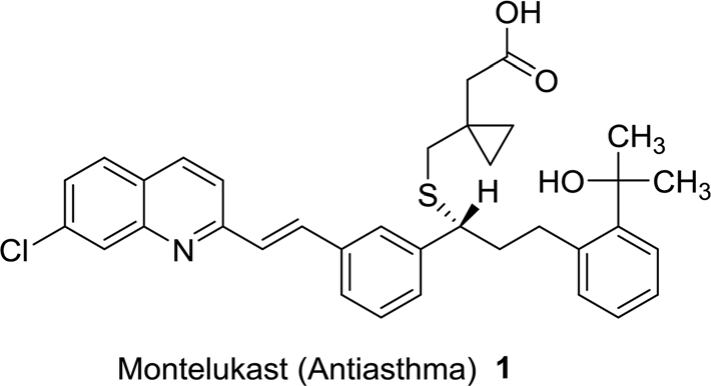
Structure of antiasthma agent incorporating quinoline nucleus.

Quinolines are aromatic compounds that consist of a benzene ring fused with a pyridine heterocyclic system. Quinolines are known also as benzo[*b*]pyridine and 1-azanaphthalene with one nitrogen atom in one benzene ring and none in the other ring or at the ring junction. Heterocycles containing a nitrogen atom possess high and interesting medicinal and pharmaceutical properties.^1–4^ Montelukast (1) is a drug used as an antiasthma agent (Fig. 1).^5^

**Fig. 2:**
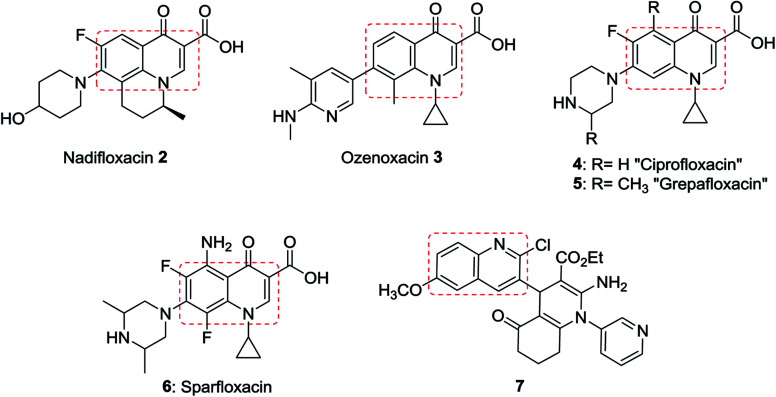
Structures of the most potent antibiotics and antimicrobial agents.

In addition, quinolines are the main core of many types of natural products,^6,7^ drugs,^8–10^ and were found in many synthetic heterocyclic compounds in order to enhance the biological and medicinal properties. Compounds incorporating quinoline ring system exhibited various biological,^11,12^ and pharmaceutical activities *e.g.* anti-tuberculosis,^13^ antiplasmodial,^14^ antibacterial,^15,16^ antihistamine,^17^ antifungal,^18^ antimalarial,^19,20^ anti-HIV,^21^ anticancer,^22^ anti-inflammatory,^23,24^ anti-hypertensive,^25^ and antioxidant activities.^26^ In addition, the use of quinolines as tyrokinase PDGF-RTK inhibitor,^27^ inositol 5′-phosphatase (SH_2_),^28^ DNA gyrase B inhibitors as *Mycobacterium tuberculosis*,^29^ and DNA topoisomerase inhibitors,^30^ were reported. Nadifloxacin (2) is a racemic fluoroquinolone launched as a topical antibiotic in Japan in 1993 to treat acne and methicillin-resistant staphylococcal infections. The *S*-enantiomer was found to be more active than the racemic mixture and had pharmacokinetic properties amenable to systemic use.^31^ Ozenoxacin (3) is a non-fluorinated quinolone with broad-spectrum activity against a variety of susceptible and resistant Gram-positive bacteria.^32^ Ciprofloxacin (4) and Grepafloxacin (5) were considered as the most effective drugs with the IC_50_s of <10 mg mL^−1^.^33^ Sparfloxacin (6) is reported as the antibiotic standard for antimicrobial tests.^34^ Hexahydro-[3,4′-biquinoline]-3′-carboxylate 7 showed a compelling antimicrobial activity at 6.25 μg mL^−1^ with a 96% inhibition (Fig. 2).^35^

**Fig. 3:**
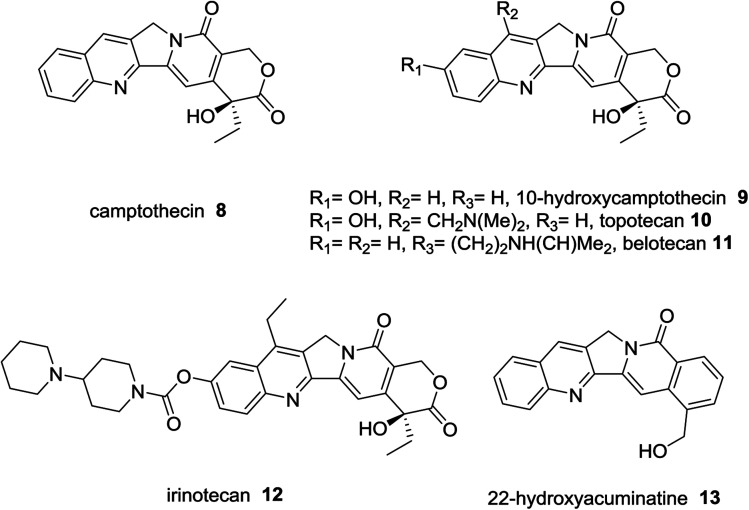
Structures of natural alkaloids and anticancer drugs.

Camptothecin (8) was extracted from Chinese plant; bark and stem of *Camptotheca acuminate* as a natural alkaloid prevent the growth of tumor cells.^36^ Topotecan (10),^37^ belotecan (11),^38^ and irinotecan (12),^39^ were reported as anticancer drugs. 22-Hydroxyacuminatine (13) is a synthetic structure related to natural products (Fig. 3).^40^

Recently, Abdel-Wahab and R. E. Khidre,^41^ have reviewed the chemistry of 2-chloroquinoline-3-carbaldehyde during the period from 1979 to 1999. The reactions are classified according to the reactivity of chlorine atom and aldehydic group. In 2012, Abdel-Wahab *et al.*,^42^ have reviewed the chemical reactions, synthetic methods and biological applications of 2-chloroquinoline-3-carbaldehydes which were reported from 1999 to 2011. The reactions are classified as addition, reduction, condensation and substitution reactions.

In continuation of the previous researches on the synthesis and reactions of quinolines,^43–47^ we described herein the literature survey of different strategies developed so far for the synthesis of 2-chloroquinoline-3-carbaldehyde and their analogs as well as to highlight their reactivity and their use as building blocks in the synthesis of variable heterocyclic systems of potent biological properties.

## 2. Synthesis of quinoline ring systems

### 2.1. Synthesis of 2-chloro-3-formylquinolines

**Scheme 1:**
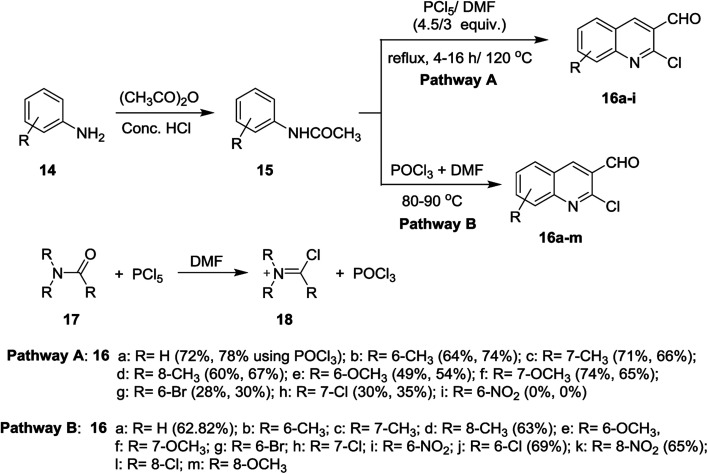
Synthesis of 2-chloro-3-formylquinolines 16a–m.

Meth-Cohn synthesis of quinolines,^48^ was reported using Vilsmeier formylating agent resulting from the reaction of DMF with phosphorus oxychloride.^49^ Treatment of acetanilides 15 with phosphorus pentachloride (4.5 equiv.) in *N*,*N*-alkylformamide (3 mol equiv.) at 120 °C for 4 h gave 2-chloro-3-formylquinolines 16a–i. Phosphorus pentachloride reacted *in situ* with *N*,*N*-alkylformamide 17 to form the formylating agent 18.^50^ Acetylation of aromatic amines 14a–m (dissolved in HCl) with acetic anhydride gave the corresponding substituted *N*-phenylacetamides 15a–m, which after treatment with Vilsmeier's reagent afforded the substituted 2-chloro-3-formylquinolines 16a–m following the methods reported by Meth-Cohn (Scheme 1).^5,49,51–56^ The better method for the synthesis of quinolines depending on the nature of the substituents in order to obtain the best yields.

**Scheme 2:**
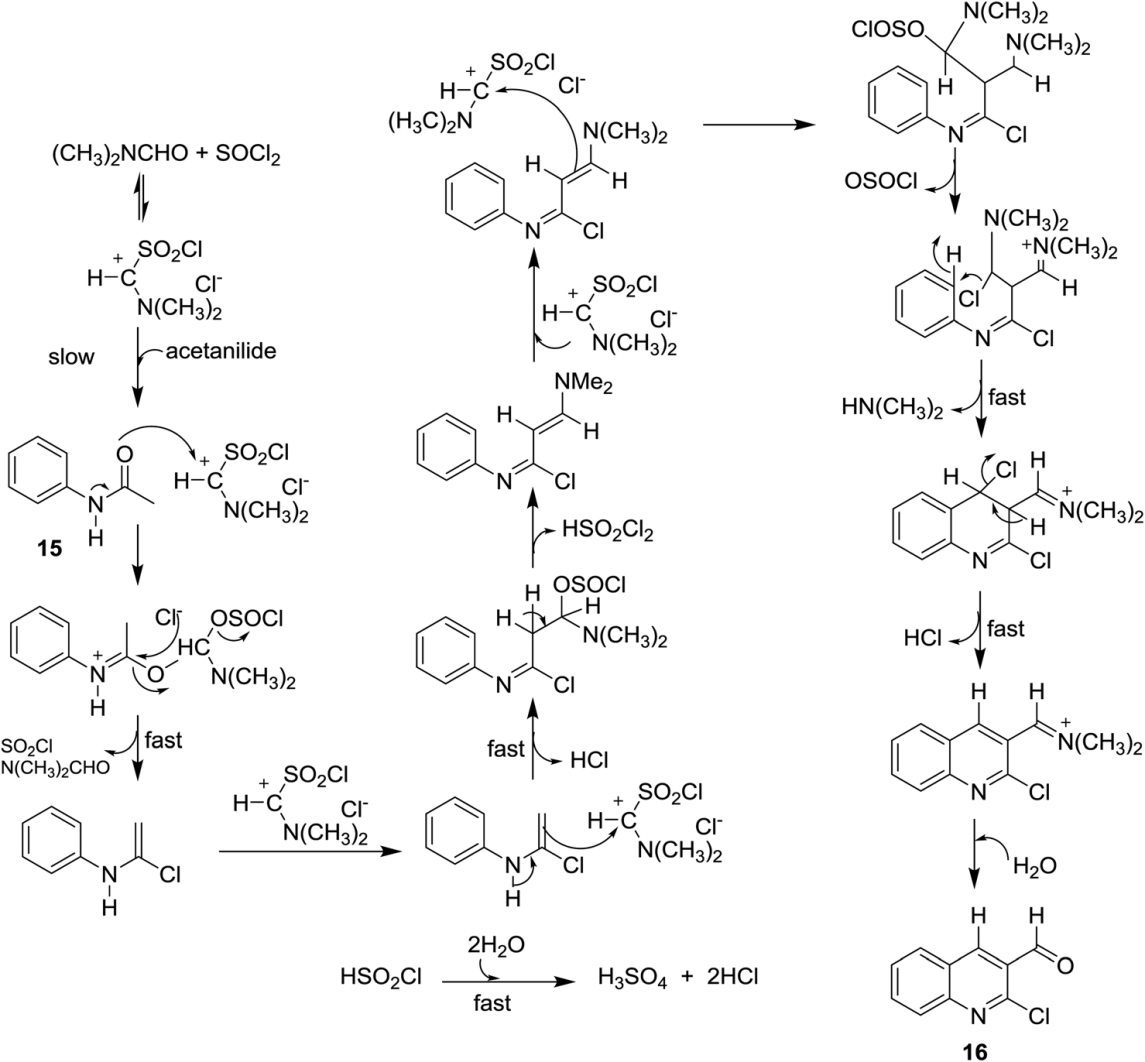
Mechanism of the formation of 2-chloro-3-formylquinoline.

Aneesa *et al.*,^57^ have reported the effect of transition metal ions such as Cu(ii), Ni(ii), Co(ii), and Cd(ii) on the synthesis of quinolines through the Vilsmeier reagent with acetanilides 15. Vilsmeier reagent was prepared by reaction of thionyl chloride (SOCl_2_) or phosphorus oxychloride (POCl_3_) with *N*,*N*-dimethylformamide (DMF). The reactions of each of acetanilide, 2-methyl-4-nitro-acetanilide, 2,4-dimethyl-acetanilide or 4-nitroacetanilide with the prepared Vilsmeier reagent afforded the respective quinolines 16 through the mechanistic pathway reported in Scheme 2. Kinetically, the followed reaction is a second order in which it depends on the Vilsmeier reagent and anilide substrate and the rate determining step is the reaction between them.

**Scheme 3:**
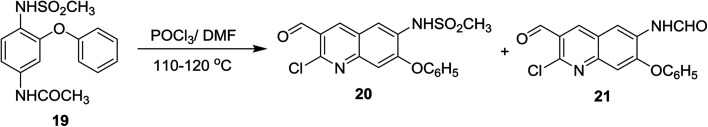
Vilsmeier–Haack formylation of acetamide 19.

Vilsmeier–Haack formylation of *N*-(4-(methylsulfonamido)-3-phenoxy-phenyl)acetamide (19),^58^ with phosphorus oxychloride in DMF gave a mixture of two substituted quinolone derivatives 20 and 21 in (1 : 1) molar ratio (Scheme 3).^59,60^

### 2.2. Synthesis of 2-oxo-3-formyl-1,2-dihydroquinolines

**Scheme 4:**
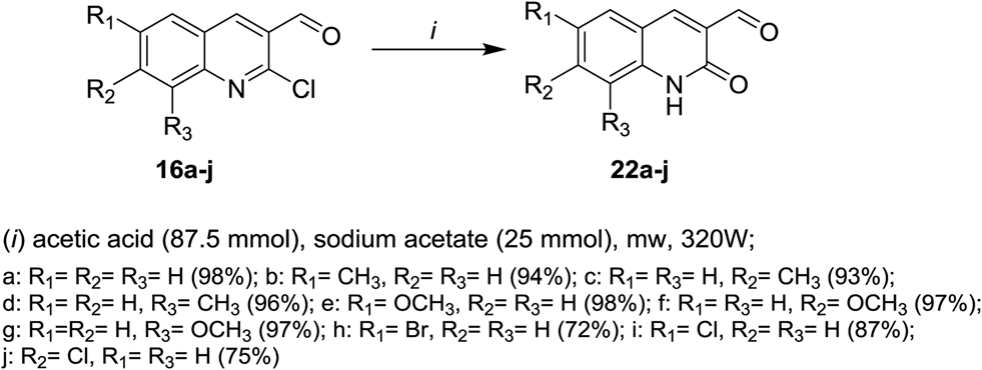
Synthesis of 3-formyl-1,2-dihydroquinolin-2-ones 22a–j.

Microwave irradiation reactions of 2-chloro-3-formylquinolines 16a–j with acetic acid containing sodium acetate at 320 W under the optimized reaction conditions afforded 6,7,8-trisubstituted-2-oxo-1,2-dihydro-quinoline-3-carbaldehydes 22a–j (Scheme 4).^61^

## 3. Reactions

### 3.1. Reductive amination of formyl group

**Scheme 5:**
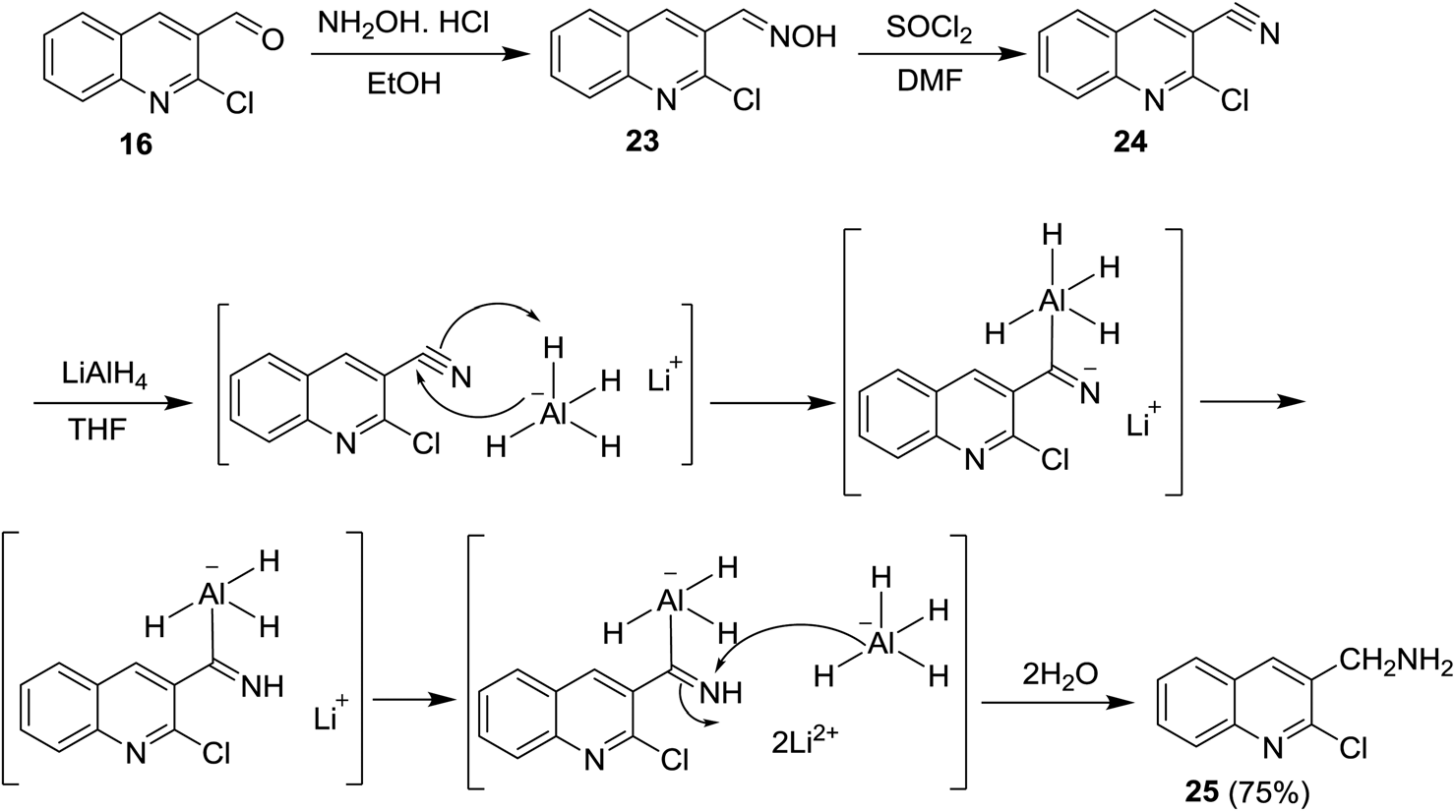
Synthesis of (2-chloroquinolin-3-yl)methanamine (25).

Reduction of C=N was achieved using lithium aluminum hydride (LiAlH_4_) and sodium boron hydride (NaBH_4_) reagents. Condensation of quinoline 16 with hydroxylamine hydrochloride followed by treatment with thionyl chloride in DMF afforded the respective 2-chloro-3-cyanoquinoline (24). Reduction of nitrile group of 24 with LiAlH_4_ in THF yielded the desired (2-chloroquinolin-3-yl)methanamine (25) in a good yield. The mechanism of reduction process of the nitrile function with LiAlH_4_ proceeded as shown in Scheme 5.^62^

**Scheme 6:**
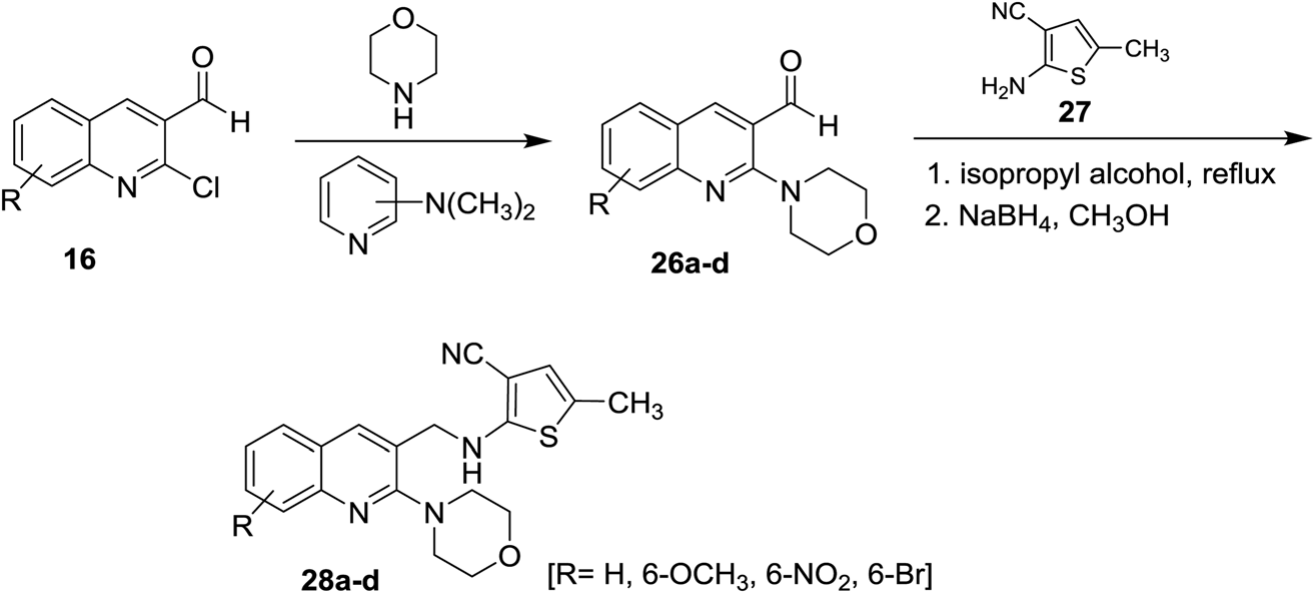
Reaction with secondary amine followed by condensation and reduction.

Treatment of quinolines 16 with morpholine in the presence of catalytic amount of dimethylaminopyridine gave 2-morpholinoquinoline-3-carbaldehydes 26a–d. Further refluxing of 26a–d with 2-amino-5-methyl-thiophene-3-carbonitrile (27) in isopropyl alcohol followed by reduction of the formed imine (C=NH) bond with sodium boron hydride in methanol afforded substituted 3-cyano-5-methyl-2-(((2-morpholinoquinolin-3-yl)methyl)amino)thiophenes 28a–d (Scheme 6). The corresponding bromo derivative 28d exhibited the highest antibacterial activity against *Escherichia coli*, *Staphylococcus aureus* and *Bacillus spizizenii* and antifungal activity against *Aspergillus Niger*, *Aspergillus Brasiliensis* and *Curvularia Lunata* microorganisms.^5^

### 3.2. Alkylation at C_2_ carbon atom

**Scheme 7:**
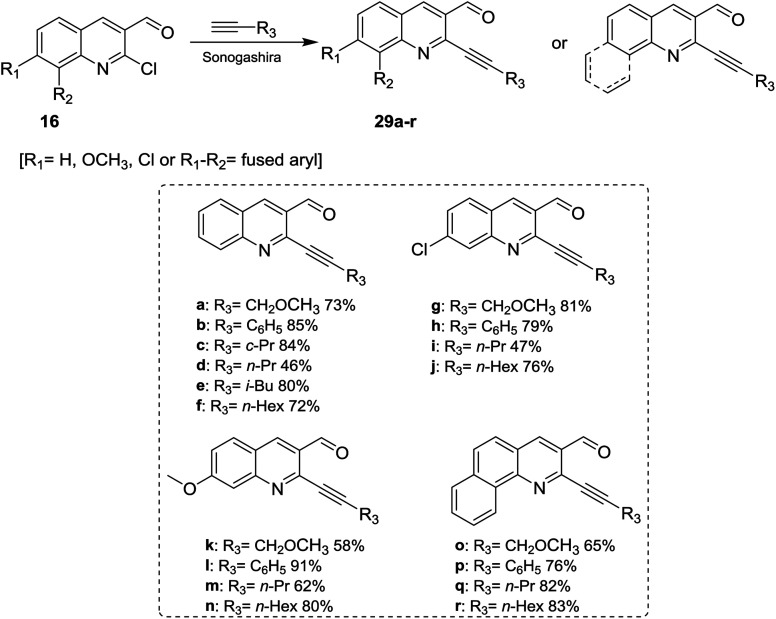
Synthesis of 2-alkyl derivatives of substituted 3-formyl-quinolines.

Sonogashira coupling reaction of alkyne derivatives with quinolones 16 in anhydrous DMF or THF containing trimethylamine in the presence of [PdCl_2_(PPh_3_)_2_] and CuI as catalysts yielded the desired 2-alkynyl-3-formyl-quinolines 29a–r (Scheme 7).^63^

### 3.3. Synthesis of Schiff bases

**Scheme 8:**
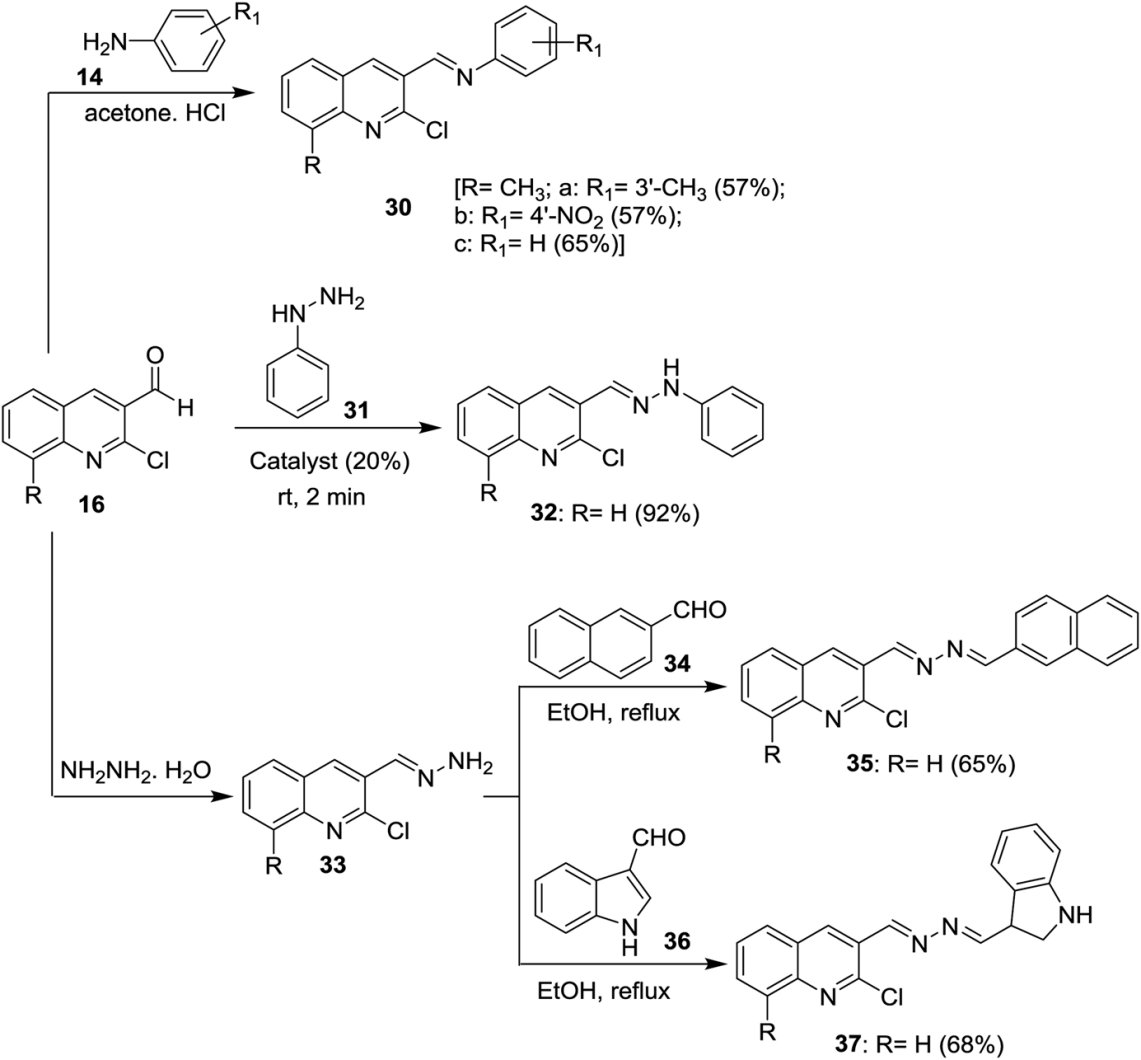
Condensation of 16 with aromatic amines and hydrazine derivatives.

Condensation of 2-chloro-8-methylquinoline-3-carbaldehyde (16d) with substituted anilines 14 in acetone afforded the respective 1-(2-chloro-8-methylquinolin-3-yl)-*N*-(substituted-phenyl)methanimine 30a–c.^51^ Schiff base 32 was synthesized by condensation of quinoline 16 with phenyl hydrazine (31) in the presence of natural surfactant (Acacia pods) in a short reaction time.^64^ Condensation of quinoline 16 with hydrazine hydrate gave 2-chloro-3-(hydrazonomethyl)quinoline (33) which reacted with each of 2-naphthaldehyde (34) and 1*H*-indole-3-carbaldehyde (36) by condensation in refluxing ethanol to produce the desired hydrazono-quinolines 35 and 37, respectively (Scheme 8).^62^ The addition of a catalyst increases the reaction yield and lower the reaction time.

**Scheme 9:**
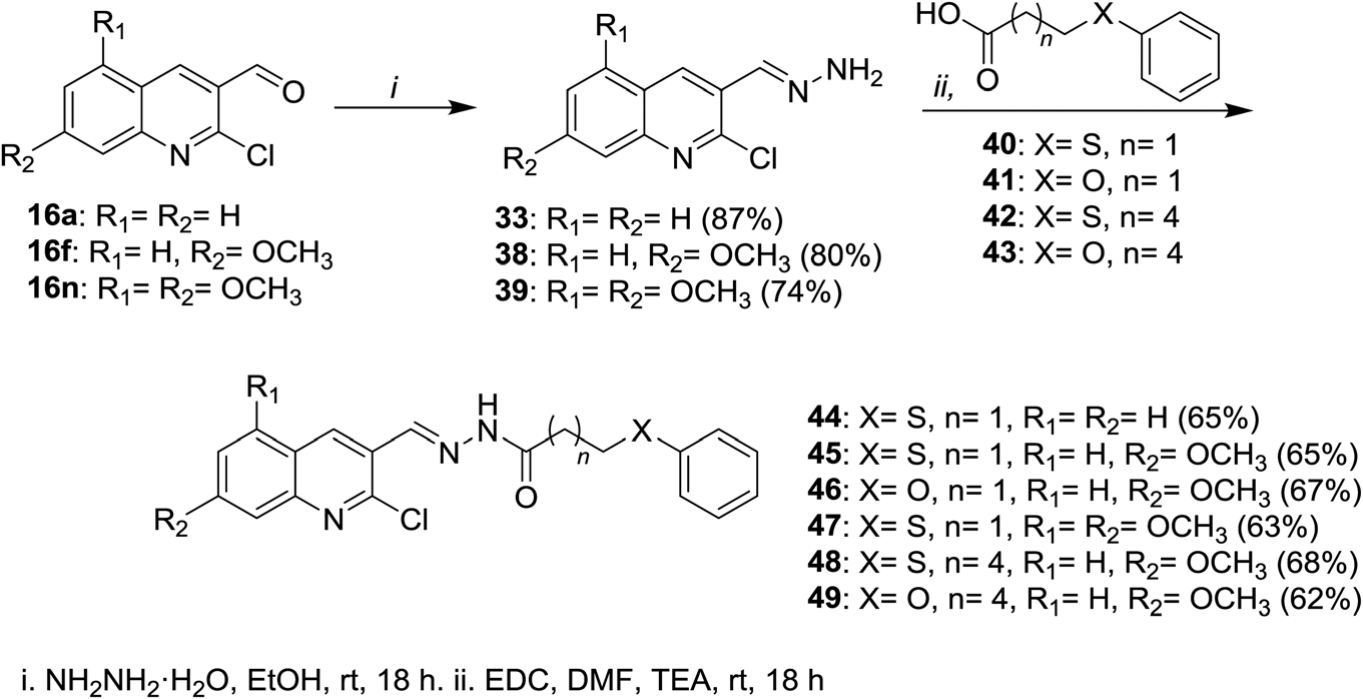
Condensation of quinolines 16 with hydrazine followed by reaction with substituted-carboxylic acids.

Condensation of each of quinolines 16a, 16f and 16n in ethanol with hydrazine hydrate gave the respective hydrazono-quinolines 33, 38 and 39, respectively. Further condensation reaction of hydrazono-quinolines 33, 38 and 39 with substituted-carboxylic acids 40–43 in DMF containing 1-ethyl-3-(3-dimethyl-aminopropyl)carbodiimide hydrochloride (EDC) and TEA afforded the corresponding amides 44–49, respectively (Scheme 9).^65^

**Scheme 10:**
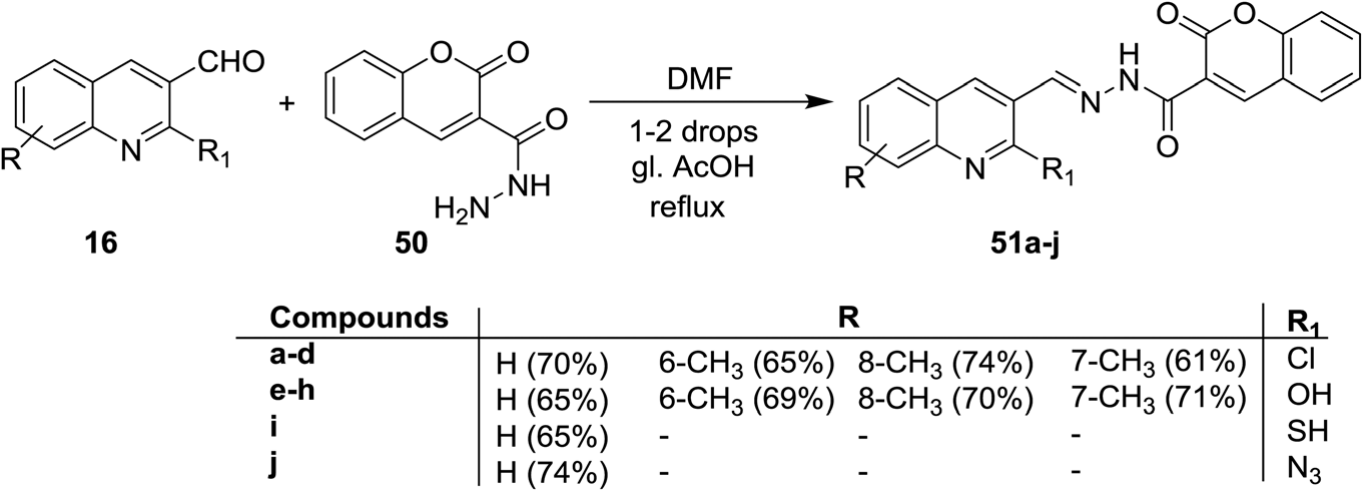
Condensation of 16 with carbohydrazide.

Condensation of quinolones 16 with 2-oxo-2*H*-chromene-3-carbohydrazide (50) in DMF containing catalytic drops of glacial acetic acid gave the respective Schiff bases 51a–j (Scheme 10). Compounds 51a–j has no antifungal activity against *A. Niger* and *A. Clavatus* microorganisms.^66^

**Scheme 11:**
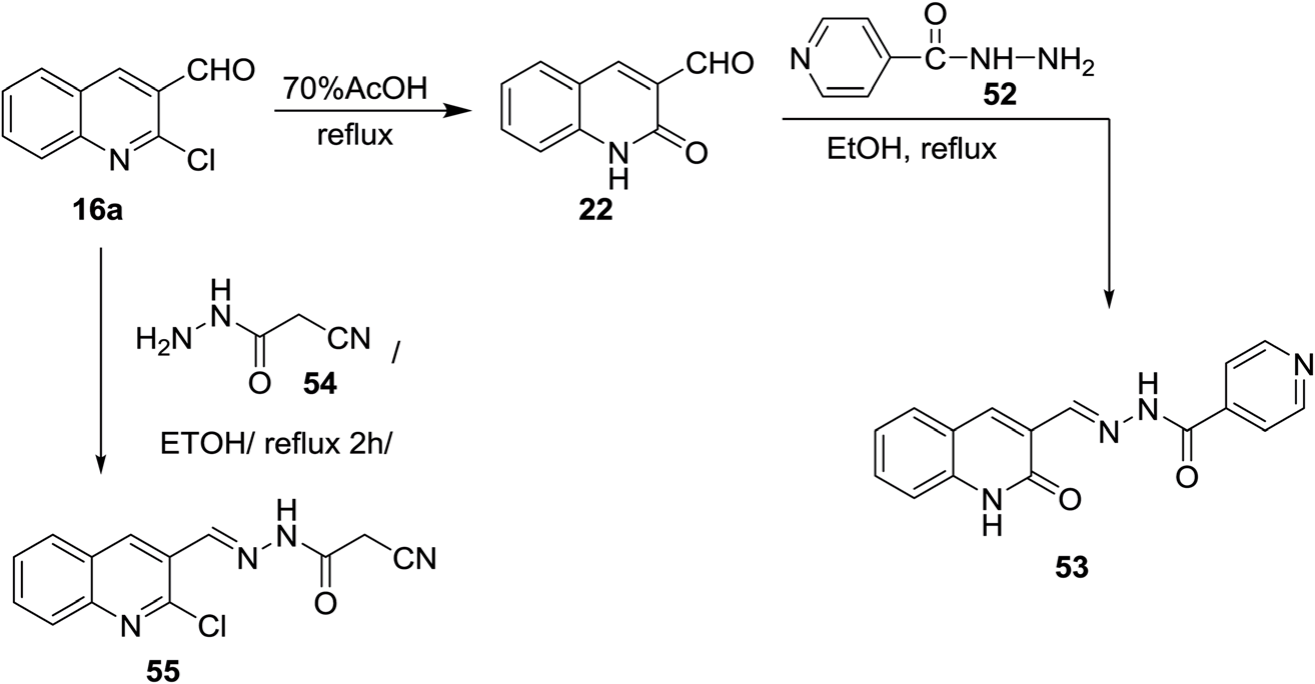
Condensation of 3-formyl-quinolin-2-one 16a with hydrazide derivatives.

Refluxing of the desired 2-chloro-3-formylquinoline (16a) in acetic acid gave quinolin-2-one derivative 22. Condensation of 22 with isonicotino-hydrazide (52) in refluxing ethanol yielded the desired benzohydrazide 53. *N*′-((2-Oxo-1,2-dihydroquinolin-3-yl)methylene)benzohydrazide (53) was used as a ligand for the preparation of complexes.^67^ Condensation of 16a with cyanoacetic acid hydrazide 54 in refluxing ethanol gave the corresponding acetohydrazide 55 (Scheme 11). 2-Cyano-*N*′-((2-oxo-1,2-dihydroquinolin-3-yl)methylene)aceto-hydrazide (55) was considered as a reactive synthetic precursor for the synthesis of several heterocycles *i.e.* pyrazoles, pyridines, coumarines and pyrazines.^68^

### 3.4. Synthesis of α,β-unsaturated ketones

**Scheme 12:**
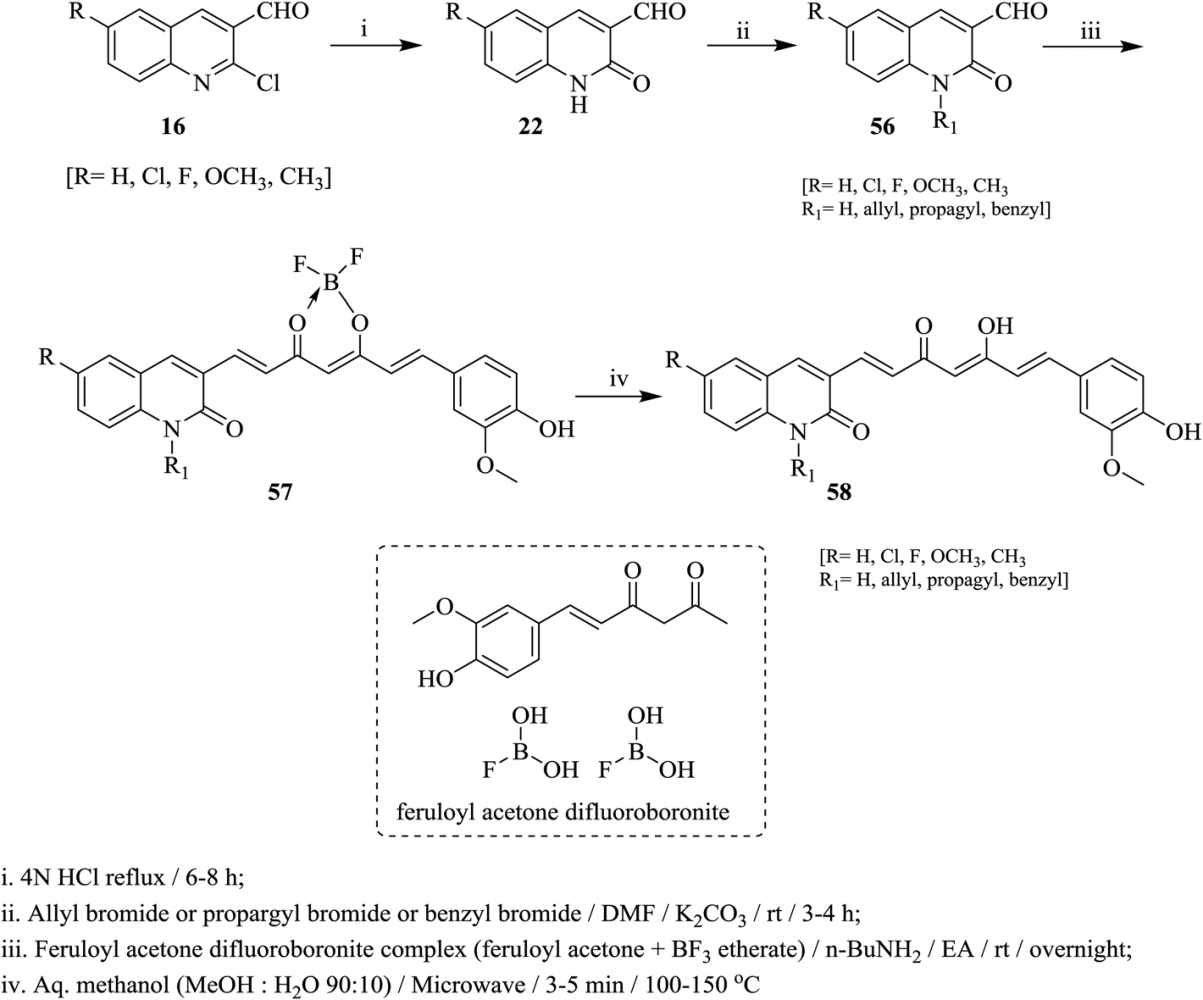
Synthesis of quinoline-curcumin analogues.

Heating of quinolines 16 with hydrochloric acid afforded the respective quinolinones 22, which was alkylated with propargyl- or benzyl- or allyl-bromides in DMF containing potassium carbonate at room temperature to give *N*-alkylquinolines 56. Condensation of *N*-alkylquinolines 56 with complex of feruloyl acetone difluoroboronite in the presence of *n*-butylamine followed by heating in a mixture of methanol/water afforded the respective 3-(5-hydroxy-7-(4-hydroxy-3-methoxyphenyl)-3-oxohepta-1,4,6-trien-1-yl)-1,6-disubstituted-quinolin-2(1*H*)-ones 58 (Scheme 12).^69^

**Scheme 13:**
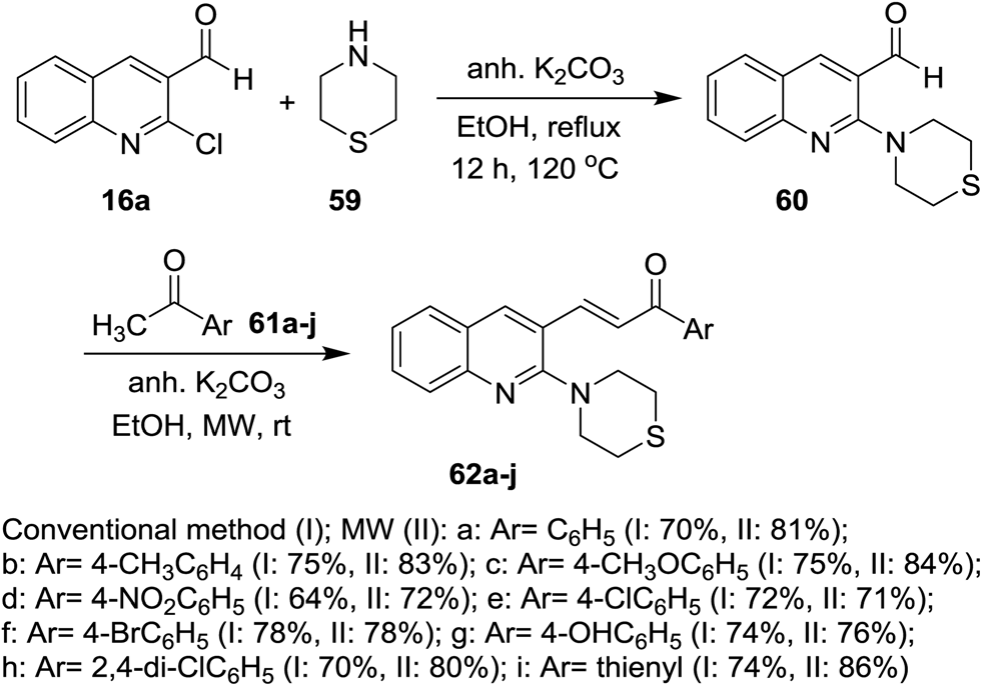
Synthesis of 1-aryl-3-(2-thiomorpholinoquinolin-3-yl)prop-2-en-1-ones.

On the other hand, 2-chloroquinoline-3-carbaldehyde (16a) reacted with thiomorpholine (59) by heating in ethanol containing anhydrous potassium carbonate to furnish 2-thiomorpholino-quinoline-3-carbaldehyde (60). Compound 60 reacted with acetophenones 61a–j in ethanol containing anhydrous K_2_CO_3_ under microwave (MW) irradiation conditions to afford the respective unsaturated ketones 62a–j, respectively (Scheme 13).^70^ The best method to obtain the highest yield is reported using MW technique.

**Scheme 14:**
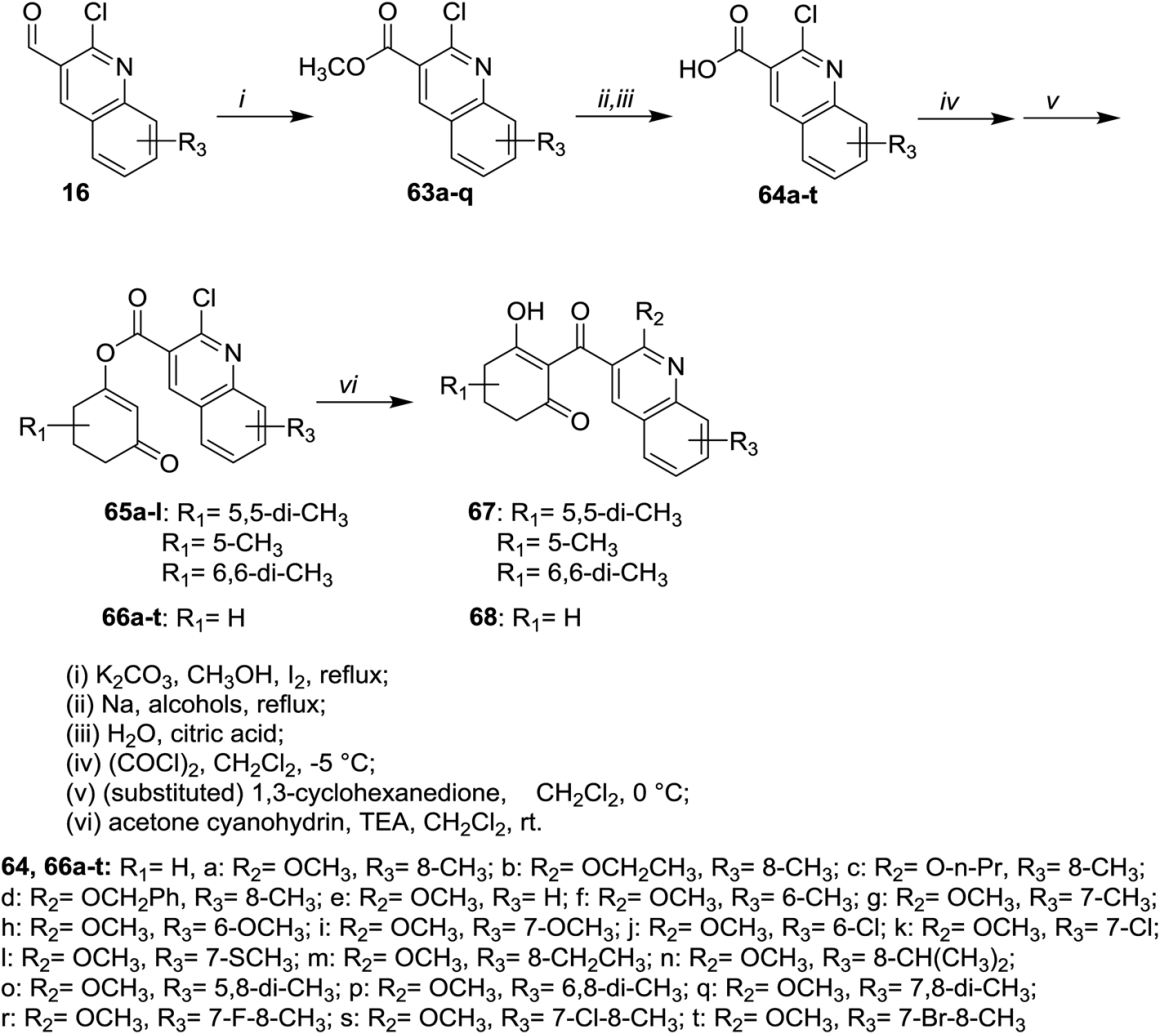
Synthesis of disubstituted-3-oxocyclohexenyl-2-chloroquinoline-3-carboxylates.

Heating of 2-chloro-3-formylquinolines 16 in methanol containing potassium carbonate and iodine gave the respective esters 63a–q, respectively. The role of iodine is to oxidize the aldehydic group to the corresponding acid followed by condensation with methanol to form the esters 63a–q (70–98%). Refluxing of 63a–q in sodium alkoxides or aryloxides followed by hydrolysis of the ester group in acid medium afforded carboxylic acids 64a–t. Esterification followed by chloroformylation of 64a–t afforded the respective quinolines 65a–l, 67 and 68 (Scheme 14).^71^

### 3.5. Amination at C_2_ of quinoline nucleus

**Scheme 15:**
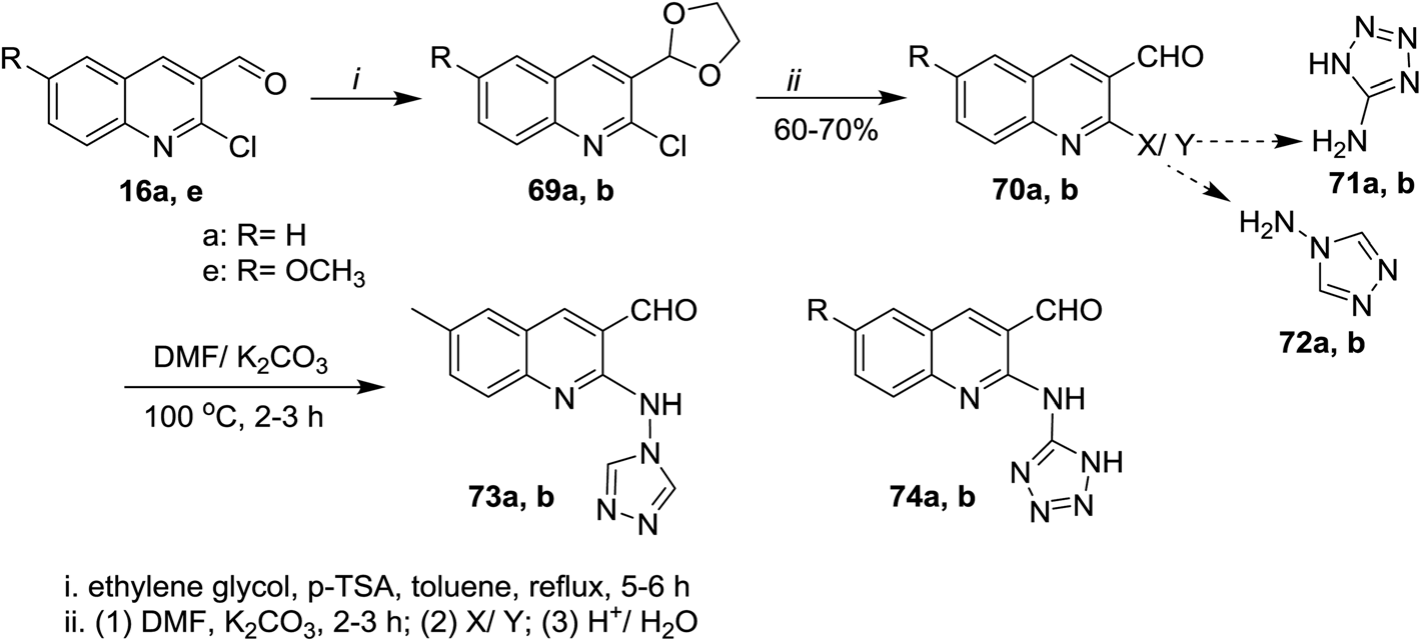
Synthesis of quinoline attached 2-aminotriazole and aminotetrazole skeletons.

Treatment of quinolines 16a and 16e with ethylene glycol in refluxing toluene containing *p*-toluenesulfonic acid led to the formation of the desired intermediate 2-chloro-3-(1,3-dioxolan-2-yl)-quinoline 69. Heating of the formed intermediate (separated in a dried organic layer from the previous step) with each of 4*H*-1,2,4-triazol-4-amine (71) or 1*H*-tetrazol-5-amine (72) in DMF containing potassium carbonate afforded the corresponding amino-triazolyl and tetrazolyl derivatives 73a,b and 74a,b, respectively. The products 73a,b and 74a,b were obtained through nucleophilic displacement of chlorine atom with the hydrogen of amino group (Scheme 15).^72^

**Scheme 16:**
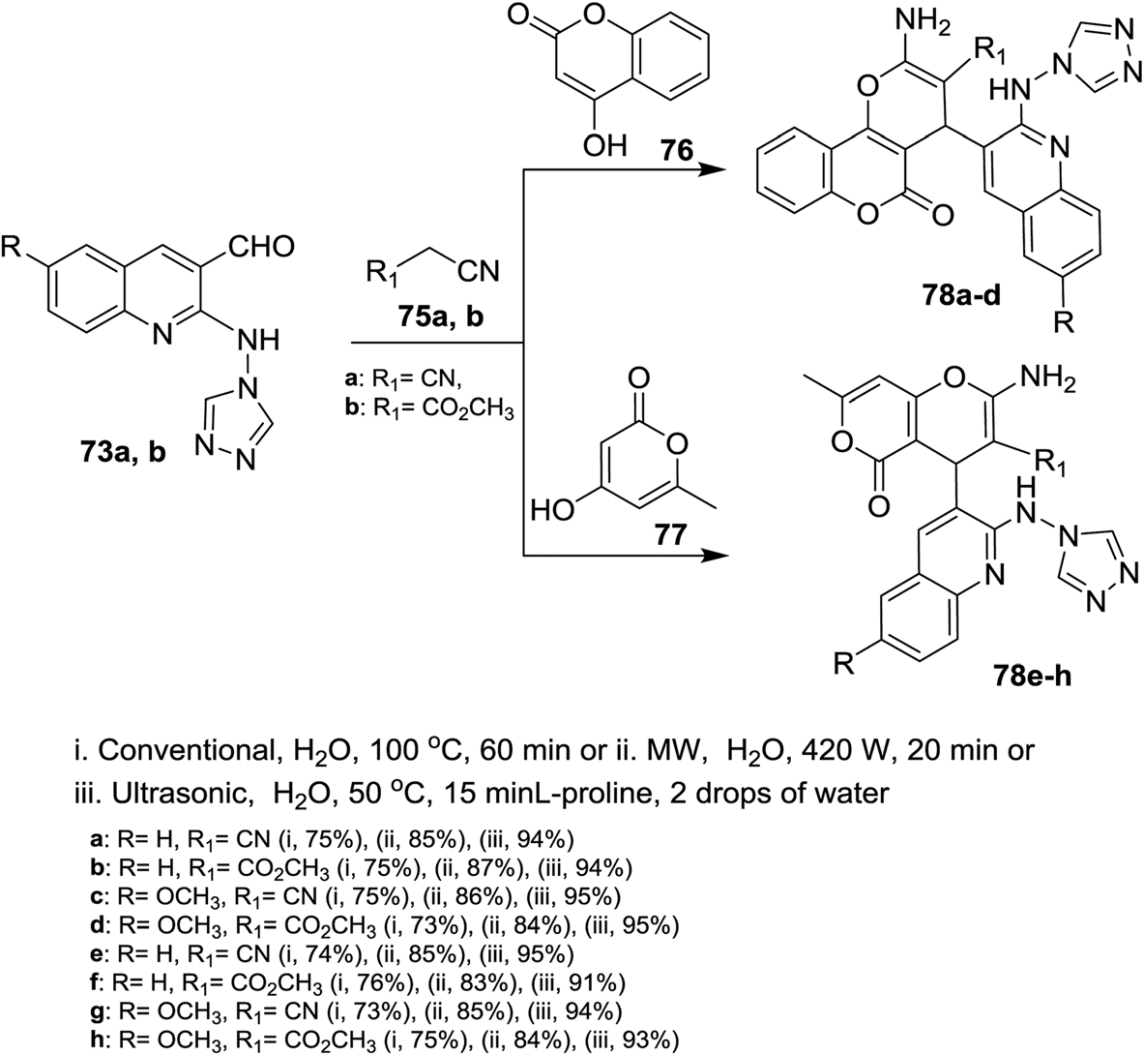
Synthesis of pyranochromenones and pyranopyranones systems.

Multicomponent reactions of 1,2,4-triazolylamino-quinolines 73a,b with each of malononitrile (75a) or methyl 2-cyanoacetate (75b) and 4-hydroxy-2*H*-chromen-2-one (76) in water under heating conditions or microwave or ultrasonic irradiation in the presence of l-proline as a catalyst afforded the respective triazolylamino-quinolinyl-pyrano[3,2-*c*]chromenones 78a–d in good to excellent yields. Similarly, compounds 73a,b reacted *via* one-pot reactions with nitriles 75a,b and 4-hydroxy-6-methyl-2*H*-pyran-2-one (77) to give the corresponding 1,2,4-triazolyl-amino-quinolinyl-pyrano[4,3-*b*]pyranones 78e–h (Scheme 16). The reactions were carried out using different catalysts such as piperidine, pyridine, triethylamine, sodium hydroxide and l-proline. The best products yield (80–85%) and lowest reactions time were achieved in case of using l-proline as a catalyst.^72^

**Scheme 17:**
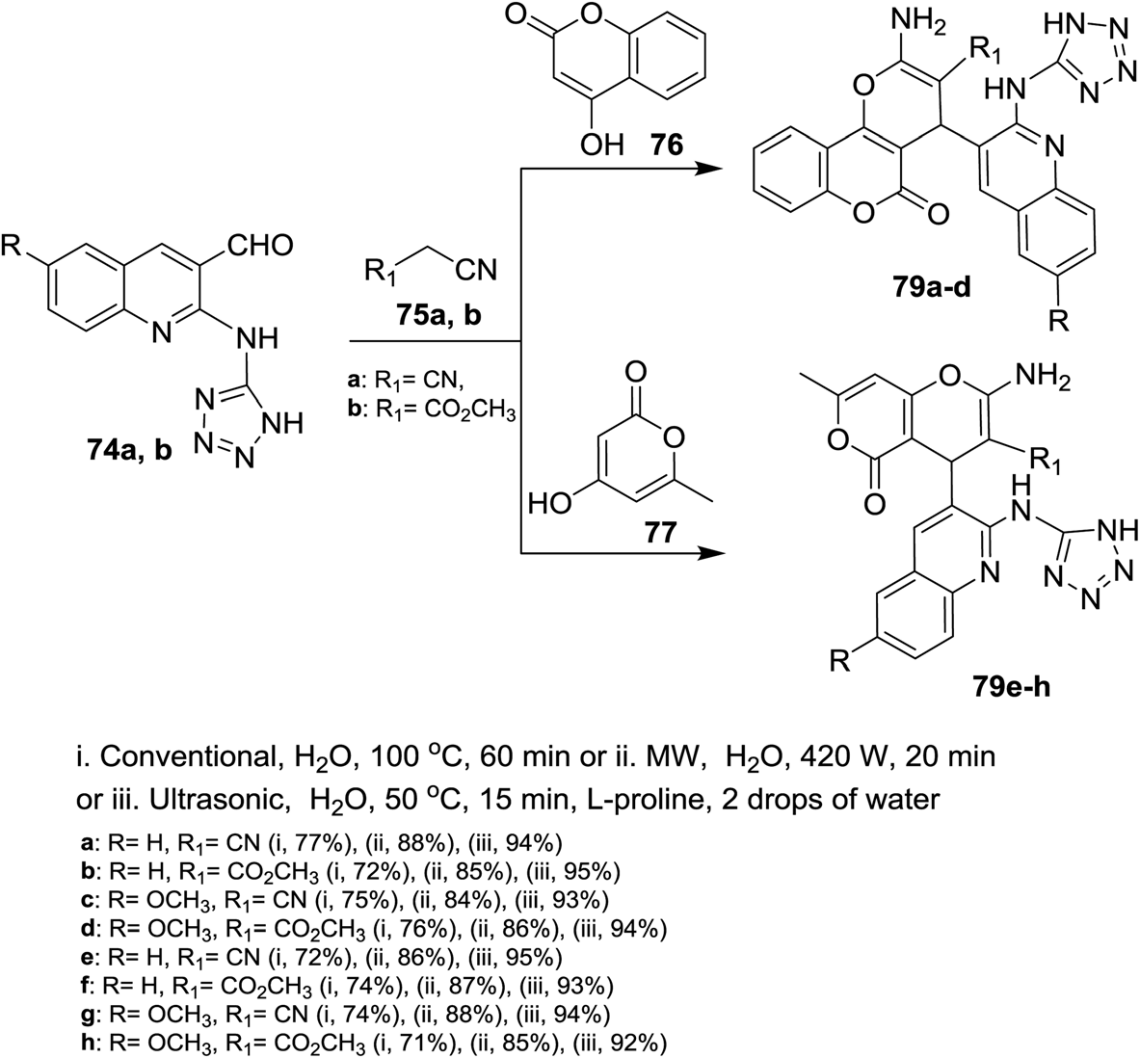
Synthesis of quinolinyl-pyrano(chromenones) and (pyranones).

Similarly, multicomponent one-pot reactions of 1*H*-tetrazolyl-amino-quinolines 74a,b with each of malononitrile (75a) or methyl 2-cyanoacetate (75b) and 4-hydroxy-2*H*-chromen-2-one (76) or 4-hydroxy-6-methyl-2*H*-pyran-2-one (77) under the optimized conditions in the presence of l-proline as a catalyst afforded the respective 1*H*-tetrazolylamino-quinolinyl-pyrano[3,2-*c*](chromenones) and (pyranones) 79a–d and 79e–h, respectively (Scheme 17).^72^

**Scheme 18:**
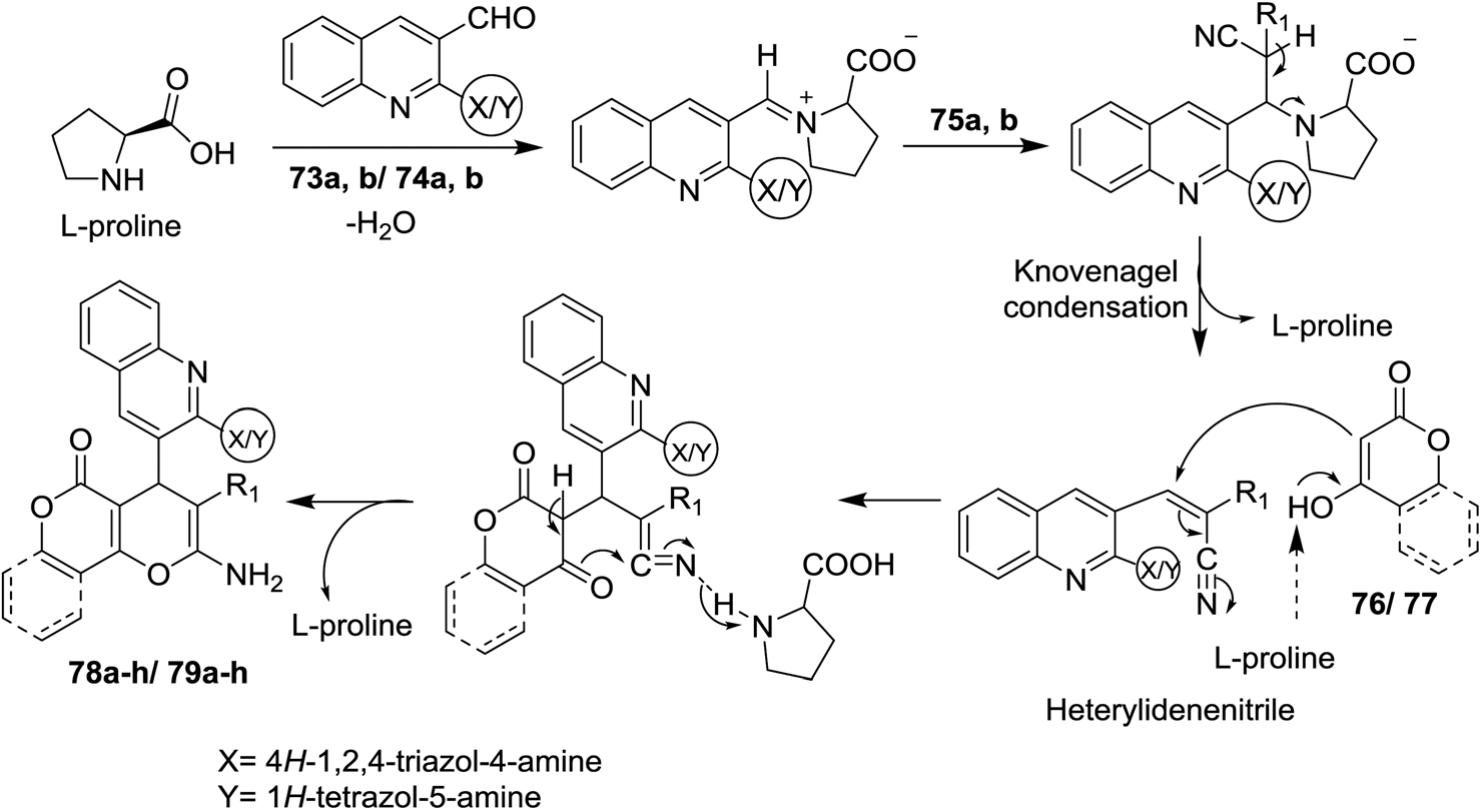
Suggested mechanism for the formation of pyrano[3,2-*c*]chromenones and pyrano[4,3-*b*]pyranones derivatives.

The reaction mechanism for the formation of 1*H*-(triazolyl)/(tetrazolyl) amino-quinolinyl-pyrano[3,2-*c*](chromenones) and (pyranones) 78a–h and 79a–h is reported through initial condensation of l-proline with *N*-heteryl-quinolines 73a,b or 74a,b followed by nucleophilic addition of the active methylenes, Knoevenagel condensation and intramolecular cyclization with the loss of the l-proline catalyst molecule (Scheme 18).^72^

**Scheme 19:**
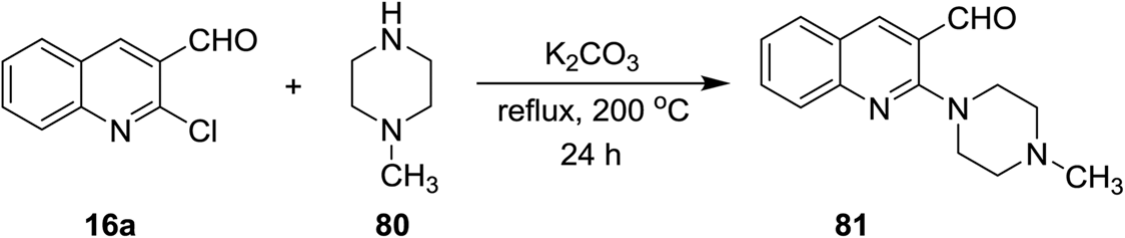
Synthesis of *N*-methyl-piperazinylquinoline.

The reaction of quinoline 16a with *N*-methylpiperazine (80) in the presence of basic medium of potassium carbonate afforded 2-(4-methyl piperazin-1-yl)quinoline-3-carbaldehyde (81) through elimination of HCl molecule (Scheme 19).^73^

## 4. Synthesis of fused heterocyclic systems

### 4.1. Synthesis of pyrrolo[3,4-*b*]quinolinone

**Scheme 20:**
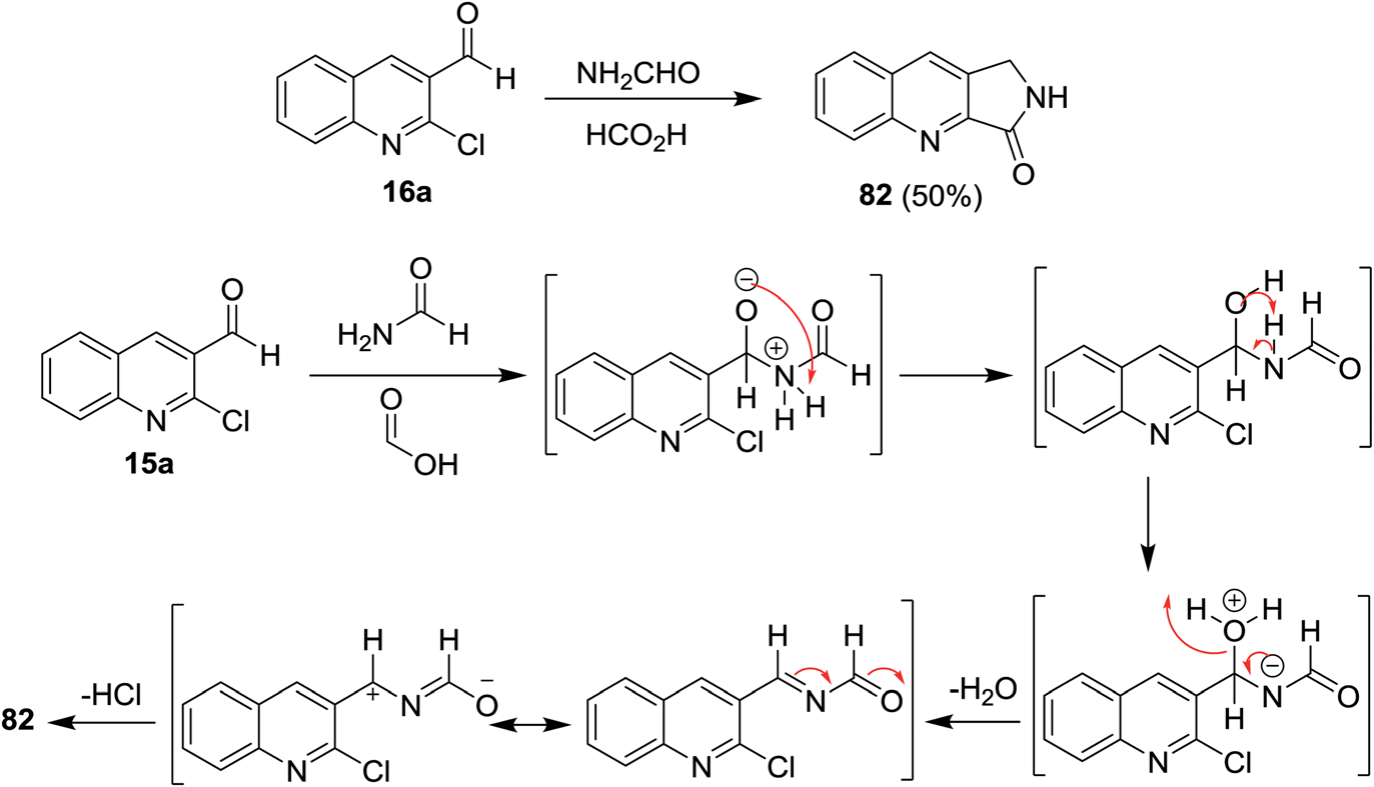
Mechanistic pathway to prepare pyrrolo[3,4-*b*]quinolin-3-one 82.

Heating of quinoline 16a with formamide and formic acid in ethanol for 8 h afforded the fused cyclic 1,2-dihydro-3*H*-pyrrolo[3,4-*b*]quinolin-3-one (82). The mechanism of the reaction was illustrated through the initial addition of an amino group of formamide to the aldehydic carbonyl of quinoline 16a, followed by condensation to form *N*-((2-chloroquinolin-3-yl)methylene)formamide intermediate. Elimination of HCl molecule from the formed intermediate gave the target product 82 (Scheme 20).^62^

### 4.2. Synthesis of pyrazoloquinolines

**Scheme 21:**
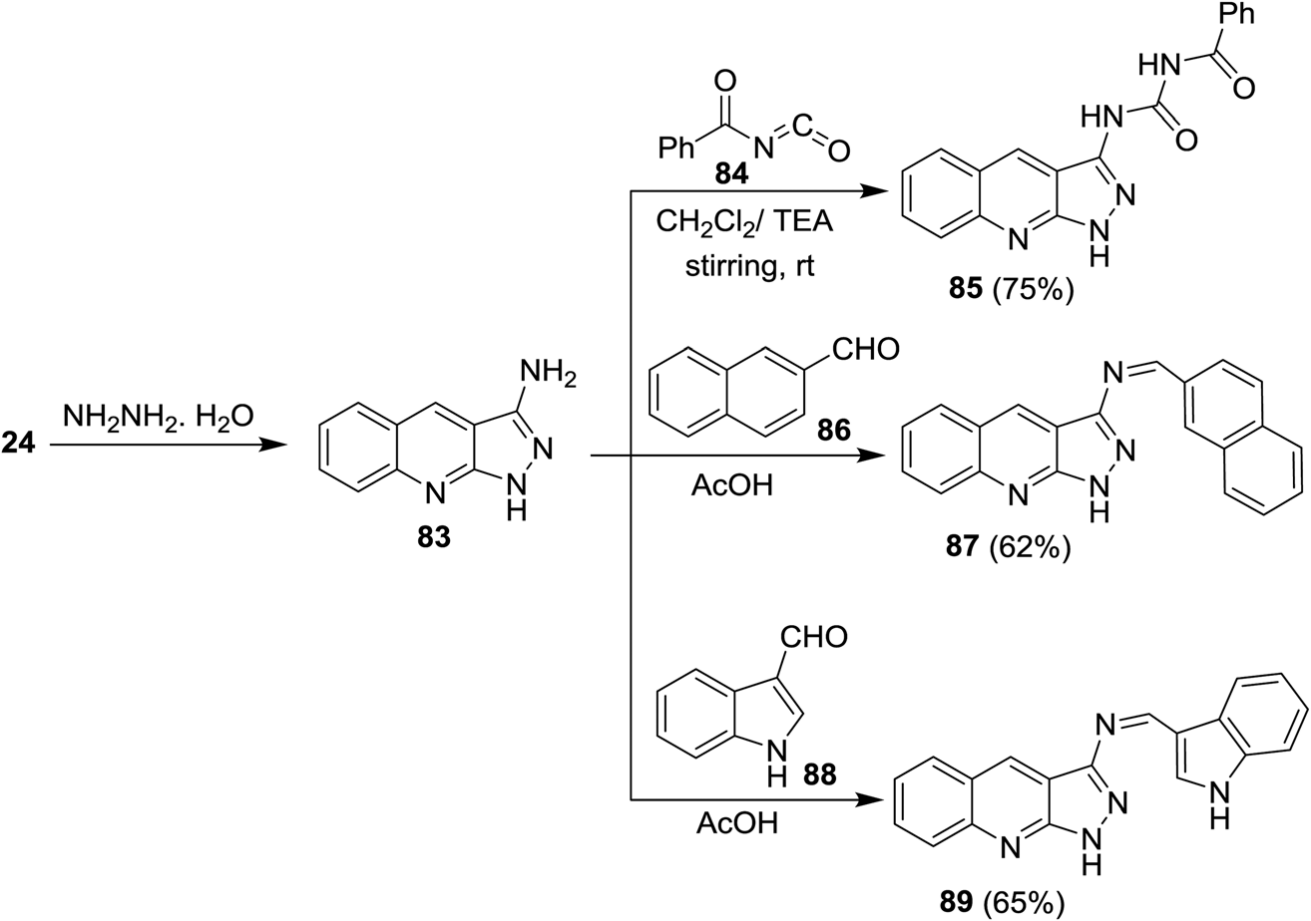
Synthesis of pyrazolo[3,4-*b*]quinolines.

Cycloaddition reaction of quinoline 24 with hydrazine hydrate gave 1*H*-pyrazolo[3,4-*b*]quinolin-3-amine (83). Treatment of 83 with benzoyl isocyanate (84) in dichloromethane containing triethylamine afforded the corresponding carbamoyl-benzamide 85. The reaction was preceded by the addition of the amino group to the imino carbonyl. Heating of 83 with each of 2-naphthaldehyde (86) and 1*H*-indole-3-carbaldehyde (88) in ethanol containing catalytic amount of acetic acid (3 drops) yielded the respective Schiff bases 87 and 89, respectively, through condensation in acidic medium (Scheme 21).^62^

**Scheme 22:**
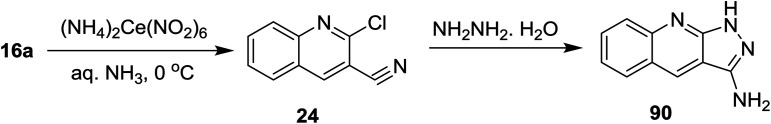
Synthesis of 3-amino-1*H*-pyrazolo[3,4-*b*]quinoline.

Treatment of quinoline 16a with aqueous ammonia in the presence of ceric ammonium nitrate gave 2-chloroquinoline-3-carbonitrile (24). Cycloaddition of 24 with hydrazine hydrate gave 1*H*-pyrazolo[3,4-*b*]quinolin-3-amine (90) (Scheme 22).^74^

**Scheme 23:**
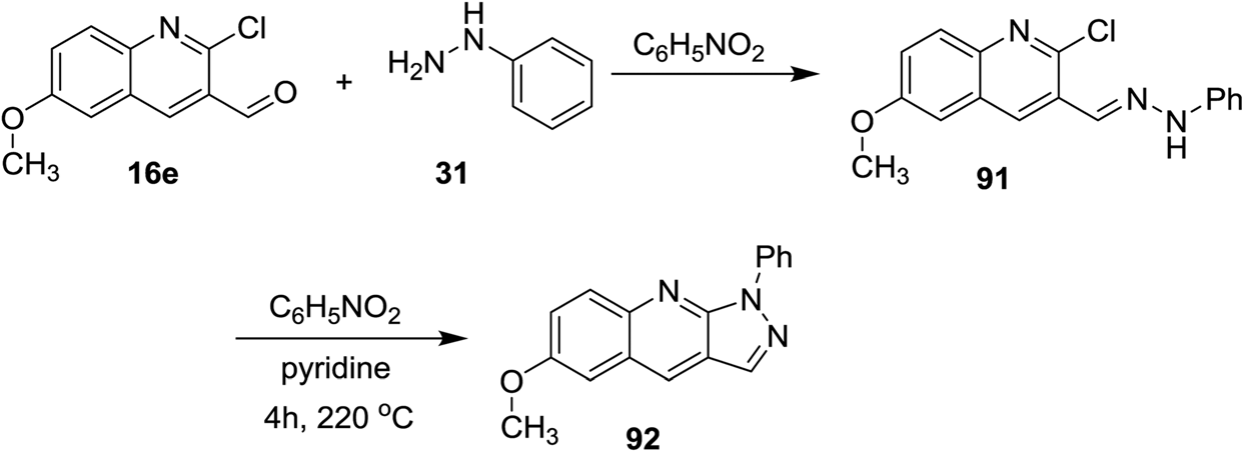
Synthesis of 1*H*-pyrazolo[3,4-*b*]quinoline.

Further condensation of 2-chloro-6-methoxyquinoline-3-carbaldehyde (16e) with phenyl hydrazine (31) gave the desired Schiff base 91, which followed intramolecular cyclization through heating in nitrobenzene containing a catalytic amount of pyridine to afford 6-methoxy-1-phenyl-1*H*-pyrazolo[3,4-*b*]quinoline (92) (Scheme 23).^74^

### 4.3. Synthesis of tetrazoloquinolines

**Scheme 24:**
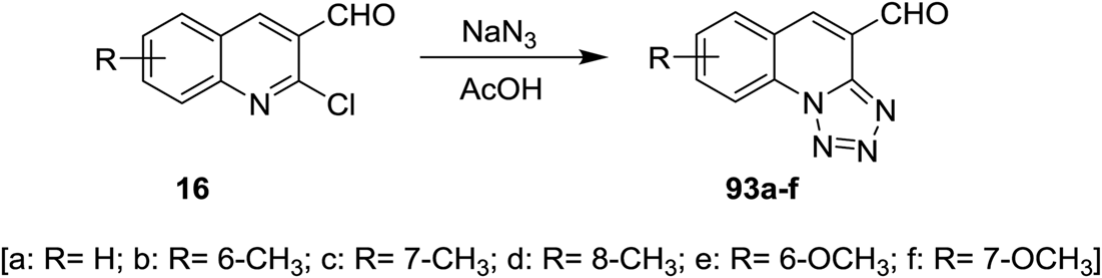
Synthesis of tetrazolo[1,5-*a*]quinoline-4-carbaldehydes.

Tetrazolo[1,5-*a*]quinoline-4-carbaldehydes 93a–f were prepared through reactions of 2-chloroquinoline-3-carbaldehydes 16 with sodium azide in acetic acid (Scheme 24).^55,75,76^

**Scheme 25:**
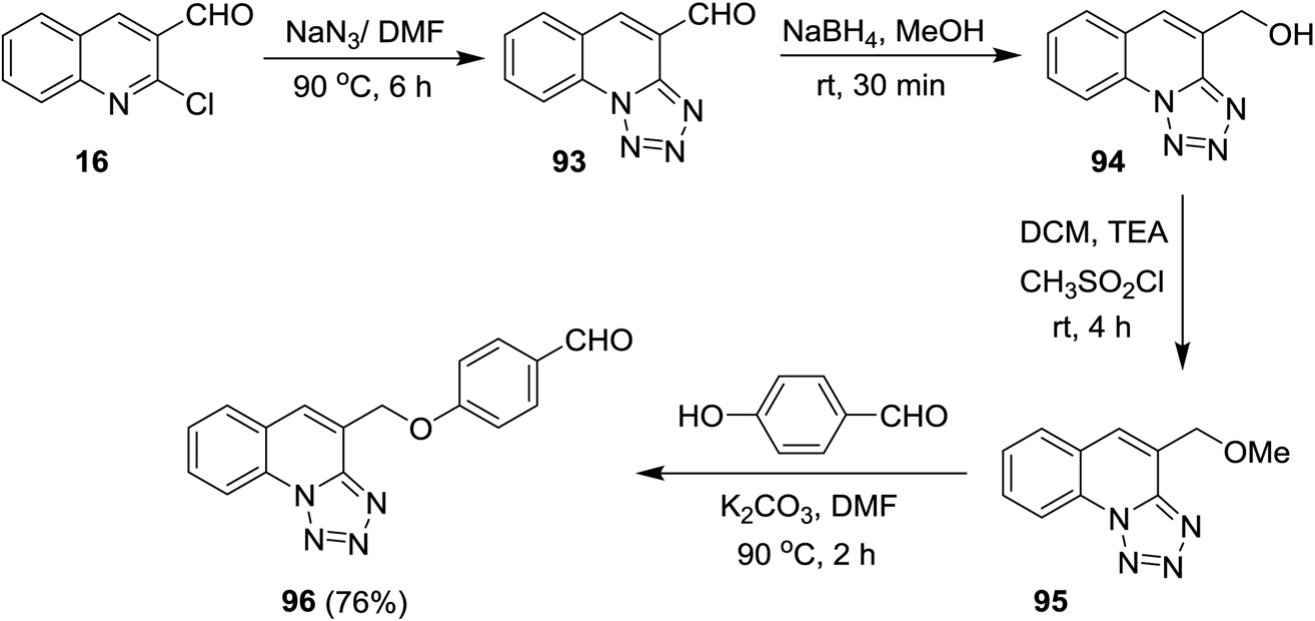
Synthesis of tetrazolo[1,5-*a*]quinolines.

Reduction of tetrazolo[1,5-*a*]quinoline-4-carbaldehyde (93a) with sodium borohydride in methanol yielded the corresponding alcohol 94 which after methylation with methanesulfonyl chloride in dichloromethane containing catalytic triethylamine gave 4-(methoxymethyl)tetrazolo[1,5-*a*]quinoline (95). Stirring of 95 with 4-hydroxybenzaldehyde in hot DMF containing potassium carbonate afforded the respective ether 96 in 76% yield (Scheme 25).^77^

**Scheme 26:**
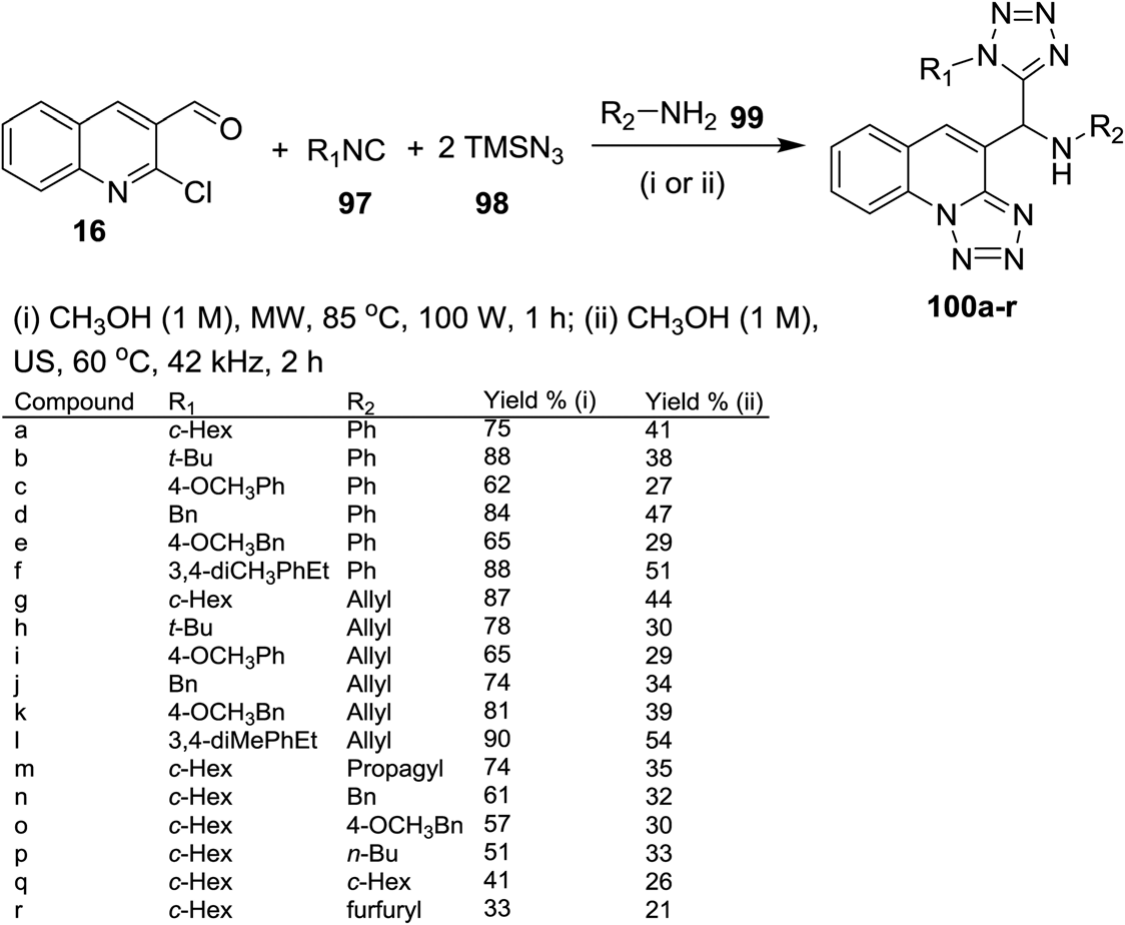
Synthesis of 1*H*-tetrazolyl-tetrazolo[1,5-*a*]quinolinyl-methanamines through Ugi-azide/S_N_Ar/ring chain azido-tautomerization method.

A four-component reaction of quinoline 16 with alkyl isocyanides 97, azido-trimethylsilane (98) and amines 99 in methanol under microwave or ultrasound (US) irradiation conditions gave the respective 1*H*-tetrazolyl-tetrazolo[1,5-*a*]quinolinyl-methanamines 100a–r in moderate yields (Scheme 26). The products 100a–r were obtained through a one-pot Ugi-azide method, nucleophilic substitution and ring chain azido-tautomerization.^78^

**Scheme 27:**
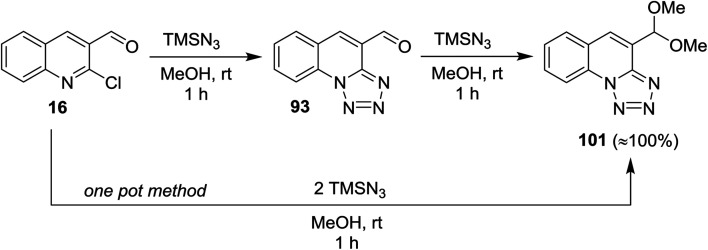
Synthesis of tetrazolo[1,5-*a*]quinoline 101 according to S_N_Ar/ring chain azido-tautomerization/nucleophilic addition conditions.

Treatment of quinoline 16 with azidotrimethylsilane in methanol gave tetrazolo[1,5-*a*]quinoline-4-carbaldehyde (93), which reacted with another mole of azidotrimethylsilane to produce the respective 4-(dimethoxy-methyl) tetrazolo[1,5-*a*]quinoline (101). The product 101 was also obtained through one-pot reaction of quinoline 16 with two moles of azidotrimethylsilane (Scheme 27). The reaction proceeded *via* nucleophilic substitution reaction followed by ring chain azido-tautomerization and nucleophilic addition.^78^

**Scheme 28:**
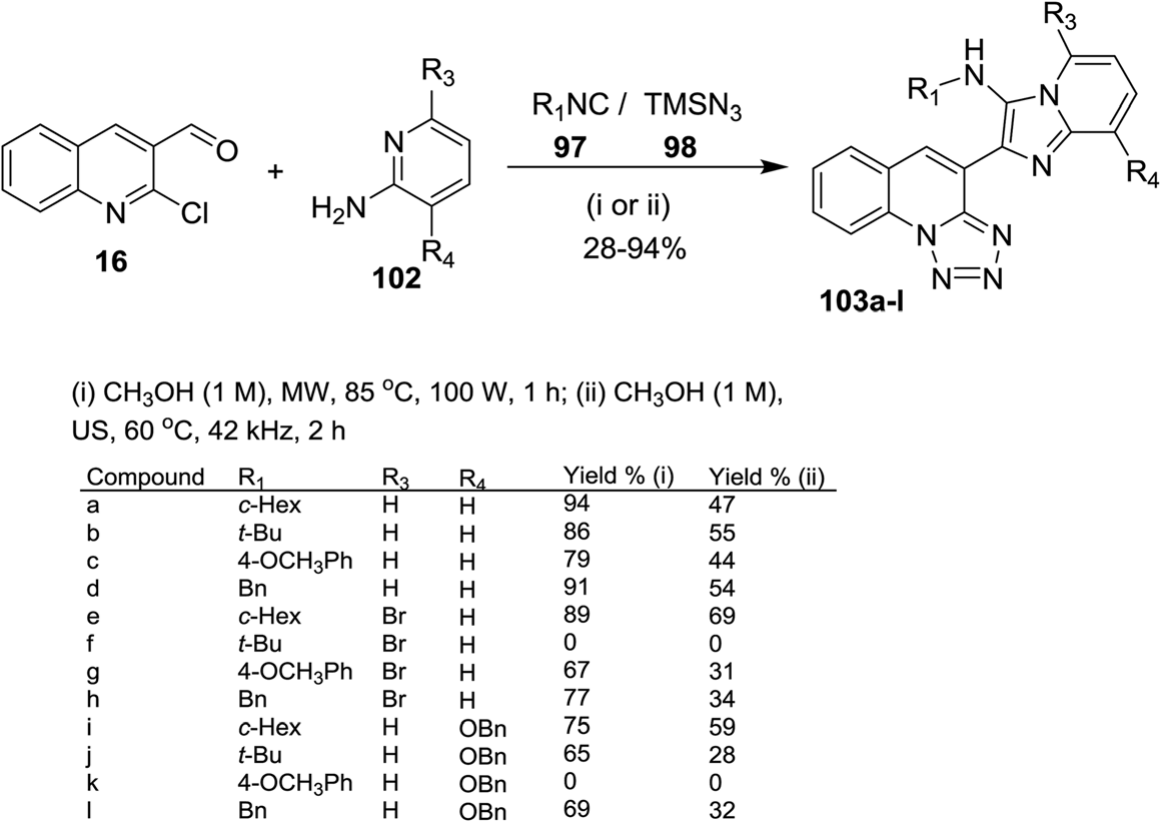
Synthesis of imidazopyridin-tetrazoloquinolines using GBB/S_N_Ar/ring chain azido-tautomerization method.

Groebke–Blackburn–Bienaymé (GBB) reaction was used for the synthesis of imidazopyridines.^79^ One-pot multicomponent reactions of quinolone 16 with alkyl isocyanides 97, azidotrimethylsilane (98) and aminopyridines 102 under optimized conditions (microwave or ultrasound (US) irradiation) afforded a series of imidazopyridin-tetrazoloquinolines 103a–l (Scheme 28). The products 103a–l prepared in better yields using MW irradiation conditions. Compound 103f was not formed due to the steric hindrance between a *tert*-butyl group and bromine atom of the pyridine ring. Also, compound 103k was not obtained due to the steric hindrance between tetrazoloquinoline ring and NH of the isocyanide group.^78^ Kishore *et al.* have reported similar synthetic routes to prepare imidazo[1,2-*a*]pyridine-chromones and the bulky substituents such as bromine and *tert*-butyl substituents did not give the products due to the high steric hinderance.^80^

**Scheme 29:**
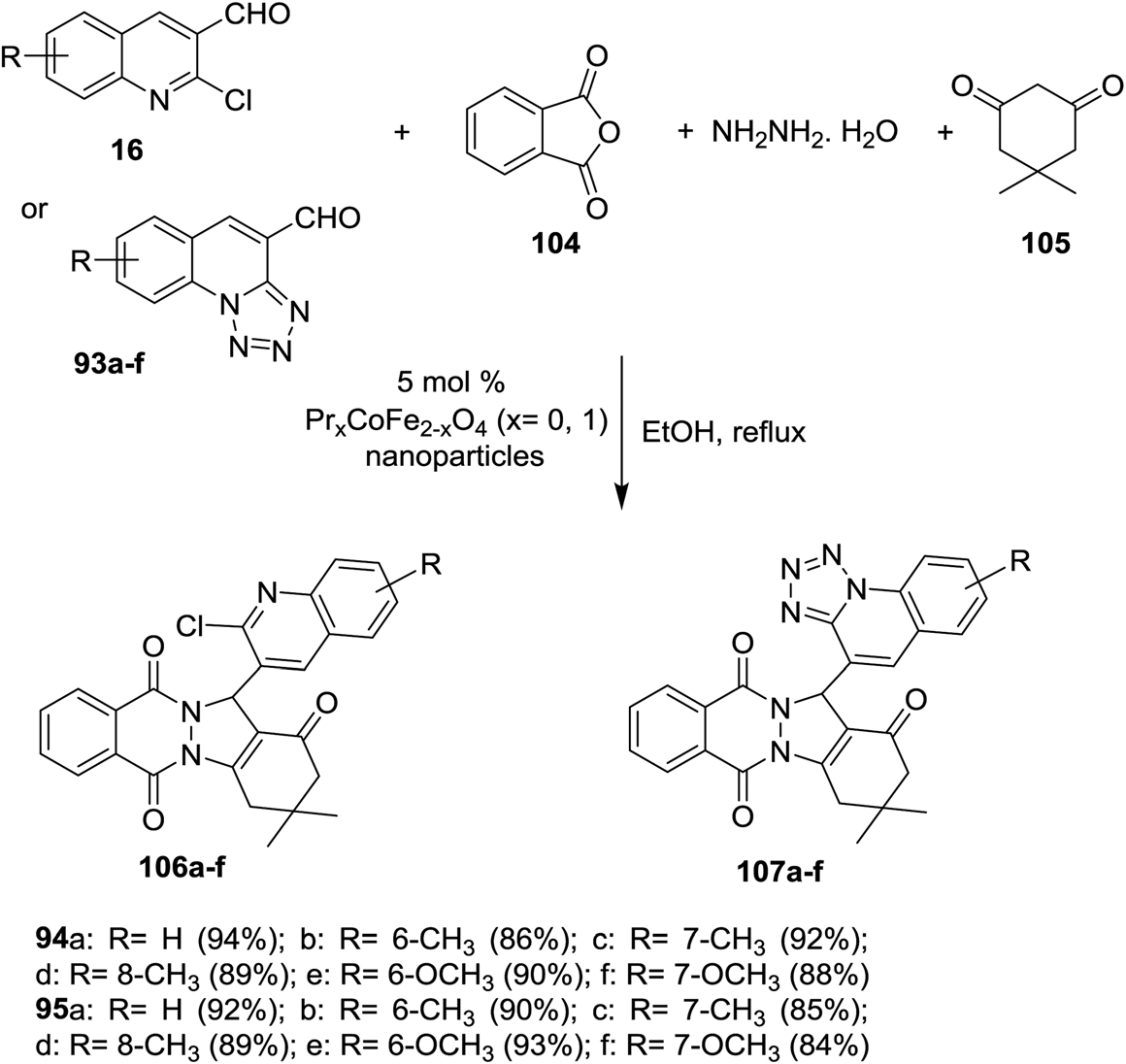
Multicomponent synthesis of tetrazoloquinolinyl-indazolo-phthalazine-triones by the aid of nanoparticles catalyst.

Multicomponent reaction of phthalic anhydride (104), hydrazine hydrate, 5,5-dimethylcyclohexane-1,3-dione (105) and each of quinolines 16 or tetrazolo[1,5-*a*]quinoline-4-carbaldehyde 93a–f in refluxing ethanol in the presence of Pr_*x*_CoFe_2_-*x*O_4_ (5 mol%) nanoparticles as a catalyst yielded quinolinyl-indazolo[1,2-*b*]phthalazine-triones 106a–f and tetrazolo[1,5-*a*] quinolinyl-indazolo[1,2-*b*]phthalazine-triones 107a–f, respectively (Scheme 29). The synthesized compounds 106 and 107a–f are considered as amalgamation biofilm inhibitors. Quinolines 106a (IC_50_ = 30 μM), 106b (IC_50_ = 46.5 μM) and 107e (IC_50_ = 46.5 μM) are potent antimicrobial agents more than the antibiotic standard ciprofloxacin.^55^

### 4.4. Synthesis of pyranoquinolinones

**Scheme 30:**
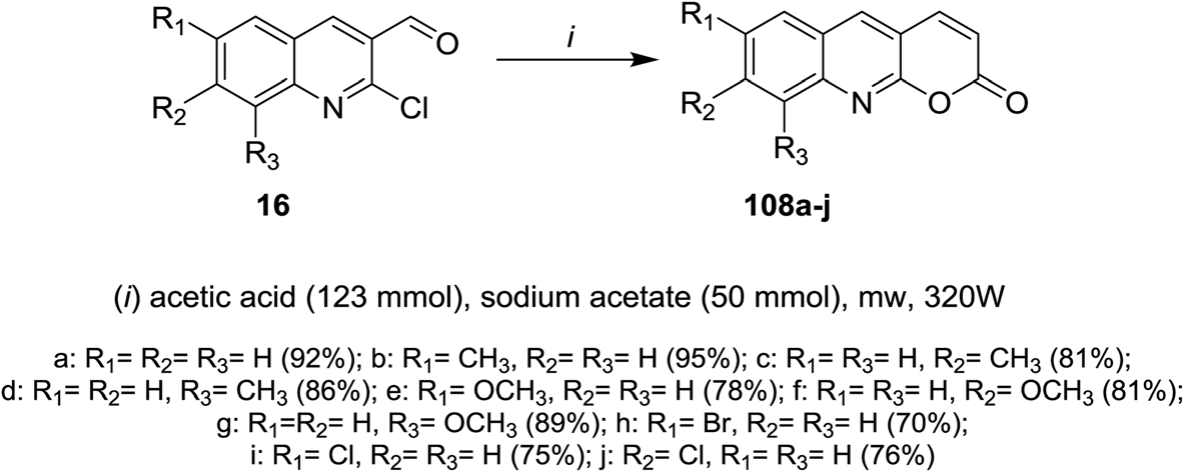
Synthesis of 2*H*-pyrano[2,3-*b*]quinolin-2-ones.

Microwave irradiation (MW) reactions of 2-chloro-3-formylquinolines 16 with concentrated buffer solution of acetic acid containing sodium acetate afforded the respective 7,8,9-trisubstituted-2*H*-pyrano[2,3-*b*]quinolin-2-ones 108a–j (Scheme 30).^61^

### 4.5. Synthesis of thiopyranoquinolines

**Scheme 31:**
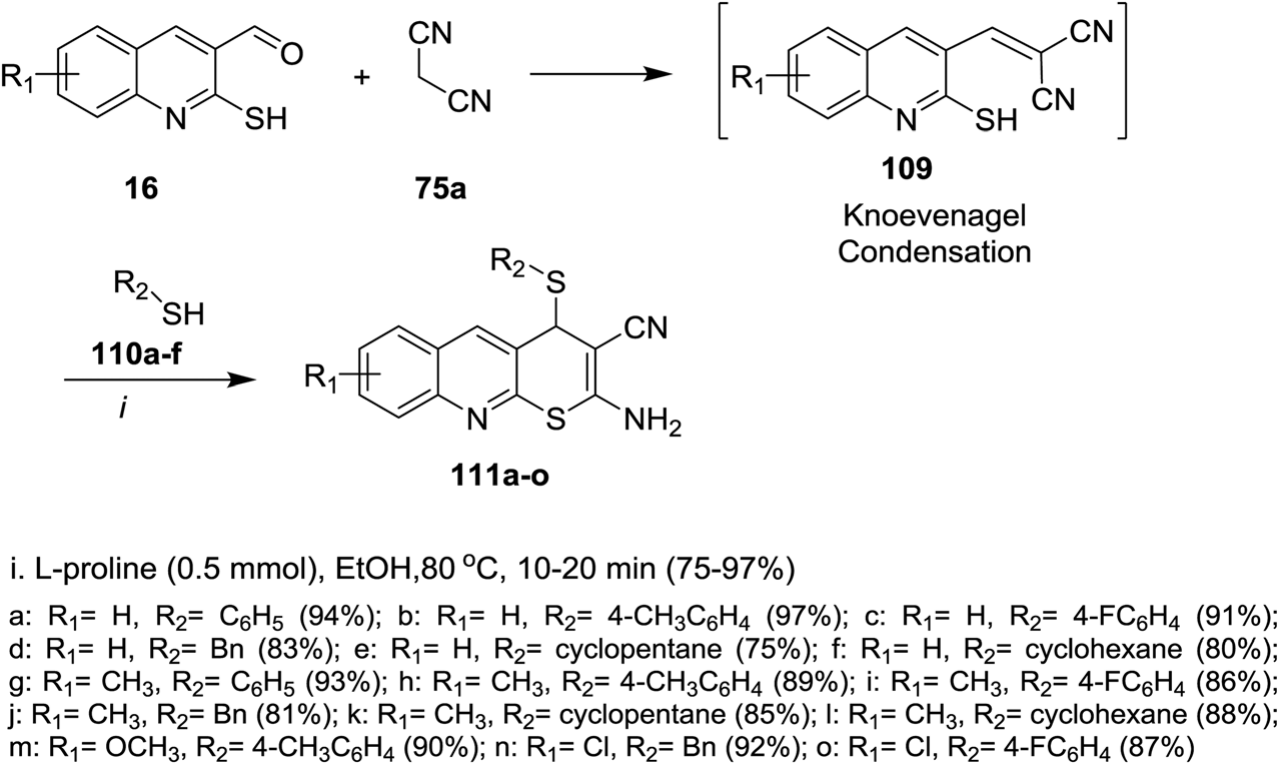
Synthesis of 4*H*-thio-pyrano[2,3-*b*]quinolines.

The reaction of 2-mercaptoquinoline-3-carbaldehyde 16 with malononitrile (75a) and thiophenol (110a) to yield 2-amino-4-(phenylthio)-4*H*-thio-pyrano[2,3-*b*]quinoline-3-carbonitrile (111a) was carried out following different reaction conditions in various solvents such as (ethanol, acrylonitrile, toluene, DMF, 1,4-dioxane, methylene chloride, water and methanol) at room temperature → 120 °C in the presence of a catalyst *e.g.* (l-proline, K_2_CO_3_, CS_2_CO_3_, piperidine, NaOH, TEA or a mixture of pyrrolidine: AcOH (1 : 1)). The highest reaction yield% (94%) was obtained using l-proline as a catalyst in ethanol at 80 °C (Scheme 31).^81^

**Scheme 32:**
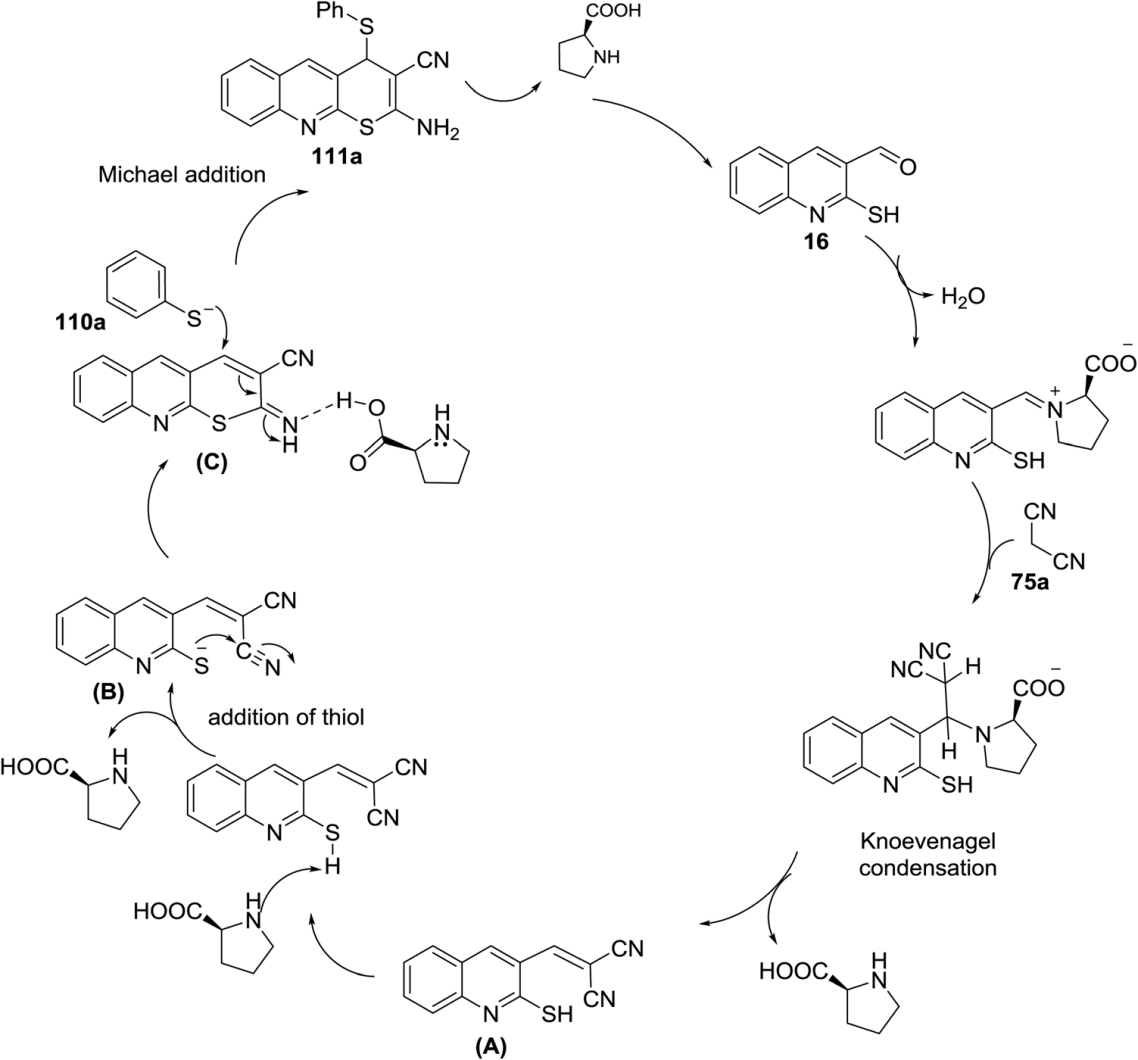
Mechanistic pathway to synthesize 4*H*-thiopyrano[2,3-*b*]quinolines.

The mechanism of the formation of thiopyrano[2,3-*b*]quinoline-3-carbonitrile 111a as shown in Scheme 32.^81^l-Proline catalyze the Knoevenagel condensation and the addition of thiol. The next step is the interaction of l-proline with the formed arylidene to facilitate the Michael addition step.

### 4.6. Synthesis of dihydrodibenzo[1,8]naphthyridinones

**Scheme 33:**
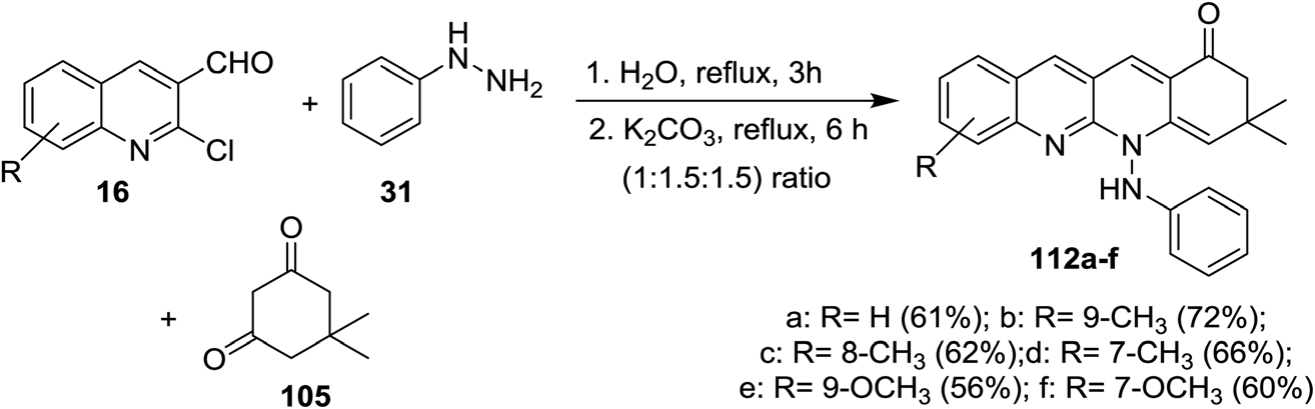
Synthesis of fused dihydrodibenzo[*b*,*g*][1,8]naphthyridin-1(2*H*)-ones.

Condensation of equimolar amounts of phenyl hydrazine (31) with 5,5-dimethylcyclohexane-1,3-dione (105) followed by reaction with substituted-2-chloro-3-formyl-quinolines 16 in the presence of potassium carbonate through one-pot multicomponent reaction afforded dihydrodibenzo[*b*,*g*][1,8] naphthyridin-1(2*H*)-ones 112a–f (Scheme 33).^82^

**Scheme 34:**
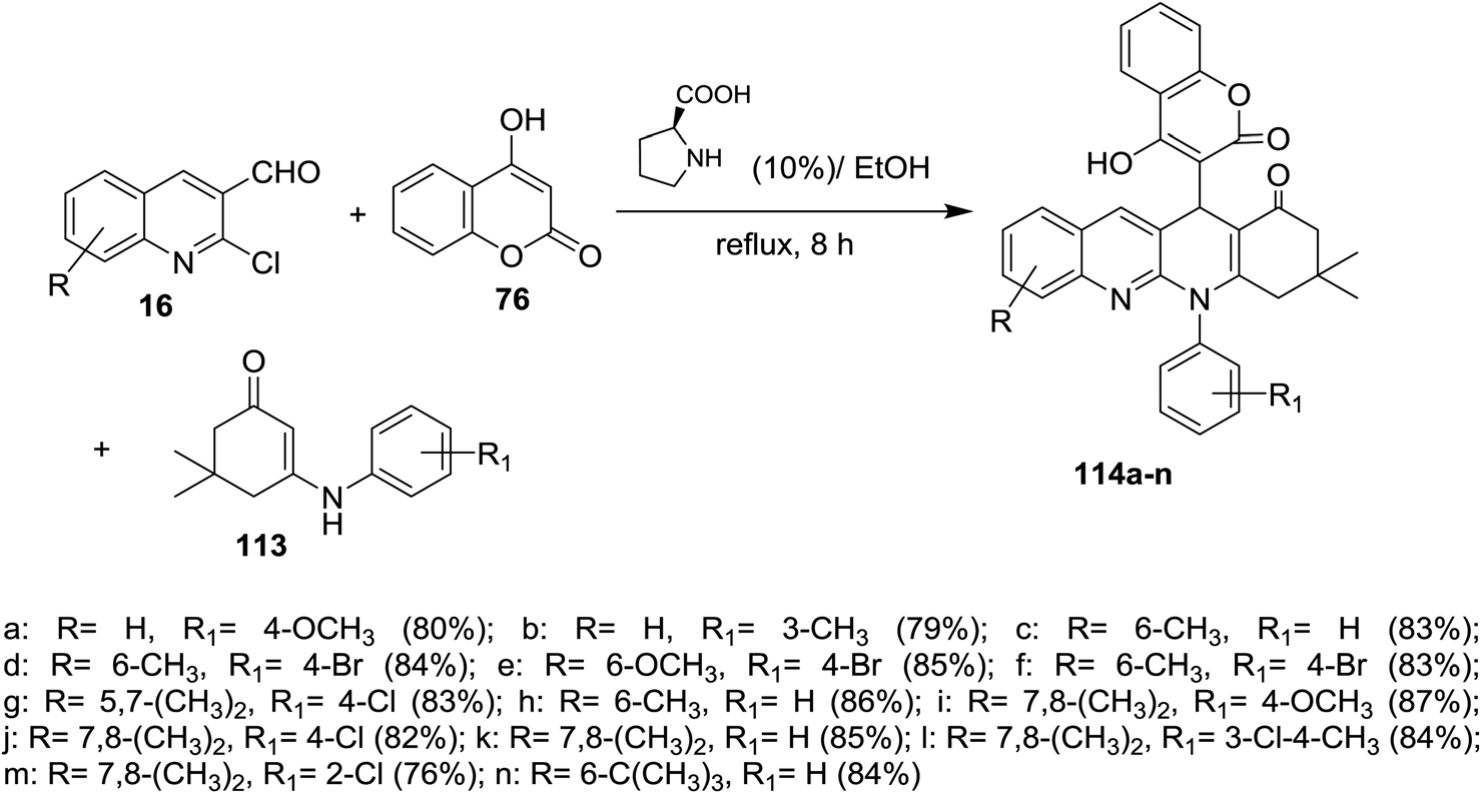
Synthesis of tetrahydrodibenzo[*b*,*g*][1,8]naphthyridin-1(2*H*)-ones.

Multicomponent reaction of substituted 2-chloro-3-formylquinolines 16 with 4-hydroxy-2*H*-chromen-2-one (76) and substituted 5,5-dimethyl-3-(phenylamino)-cyclohex-2-en-1-ones 113 in refluxing ethanol containing l-proline yielded the respective tetrahydrodibenzo[*b*,*g*][1,8]naphthyridin-1(2*H*)-ones 114a–n (Scheme 34). It was noticed that the best yields were obtained using l-proline as a catalyst in refluxing ethanol rather than using catalysts such as cesium carbonate or sodium hydroxide or piperidine, *etc*… and solvents such as water or DMF or chloroform or toluene or acetonitrile.^83^

**Scheme 35:**
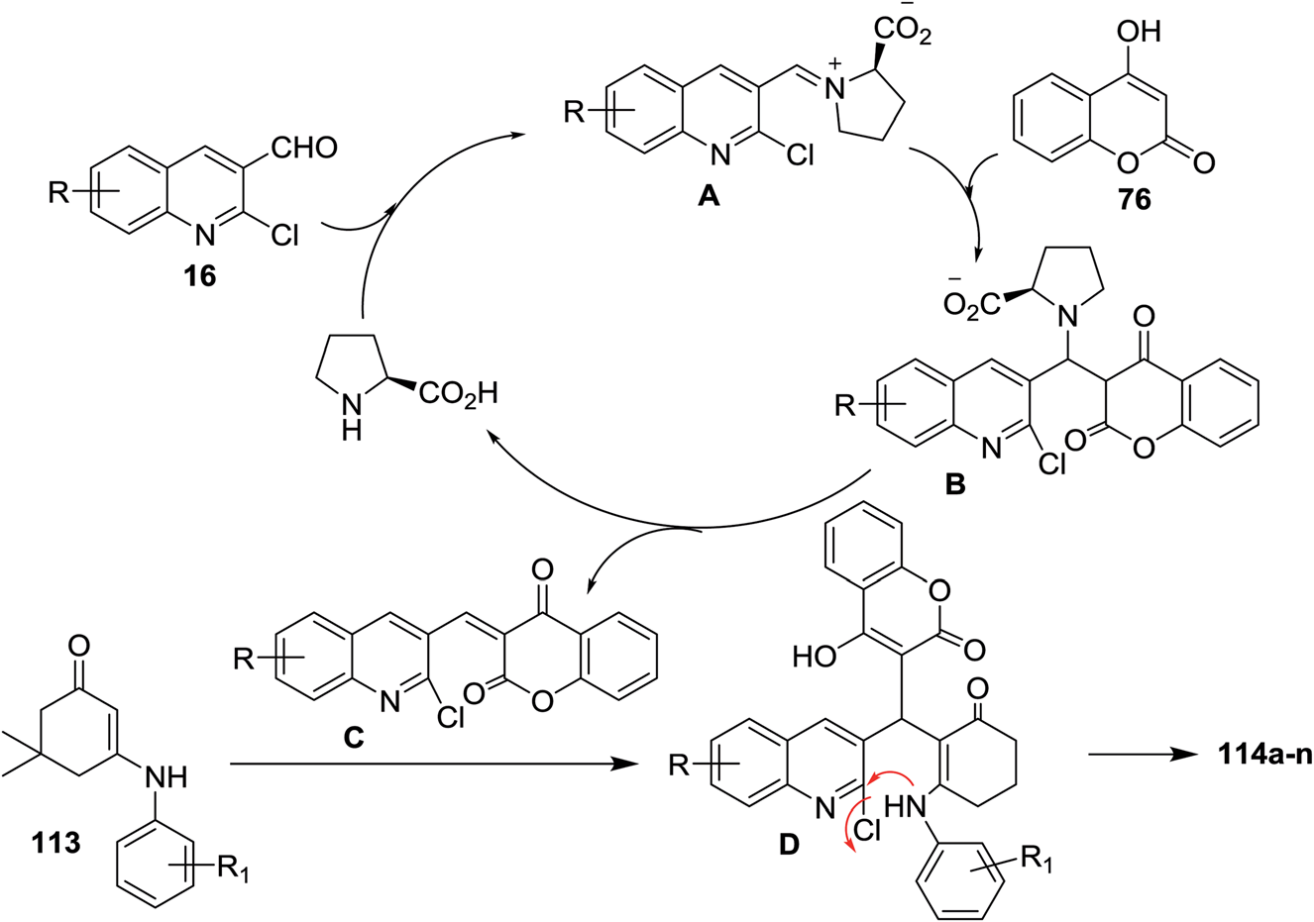
Mechanistic pathway for the synthesis of compounds 114a–n.

The mechanism of formation of compounds 114a–n is explained as shown in Scheme 35. The formation of iminium ion (intermediate A) was catalyzed by l-proline by a reversible reaction with aldehydes 16. Knoevenagel condensation of 76 with the reactive iminium ion (intermediate A) produces intermediate (B). Elimination of l-proline led to the formation of intermediate (C) which reacted with enaminone 113 to produce the intermediate D. Finally, intramolecular cyclization of intermediate D gave tetrahydrodibenzo[*b*,*g*][1,8]naphthyridin-1(2*H*)-ones 114a–n.^83^

**Scheme 36:**
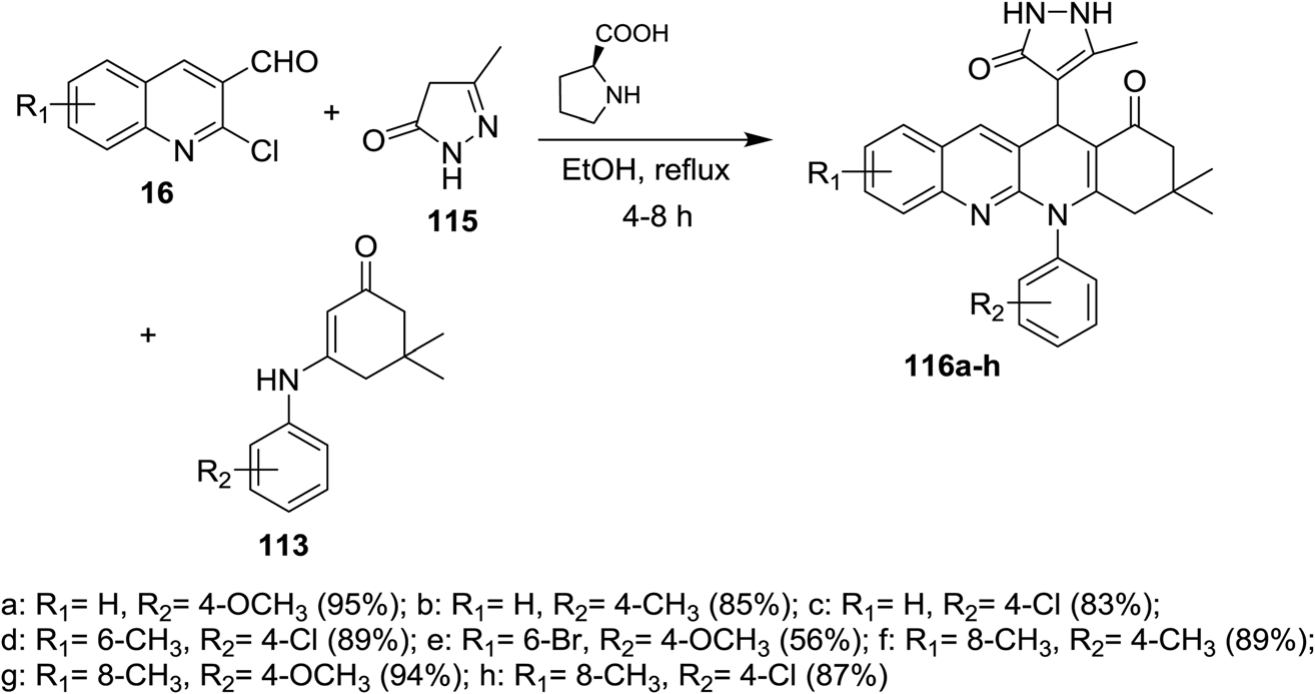
Synthesis of tetrahydrodibenzo[*b*,*g*][1,8]naphthyridin-1(2*H*)-ones.

A series of tetrahydrodibenzo[*b*,*g*][1,8]naphthyridin-1(2*H*)-ones 116a–h were prepared through multicomponent reactions of substituted 2-chloro-3-formylquinolines 16 with 5-methyl-2,4-dihydro-3*H*-pyrazol-3-one (115) and 5,5-dimethyl-3-(phenylamino)cyclohex-2-en-1-one 113 by heating in ethanol in the presence of catalytic l-proline (Scheme 36).^84^

### 4.7. Synthesis of pyrimido[1,8]naphthyridines and chromenoquinolinyl-pyrimidines

**Scheme 37:**
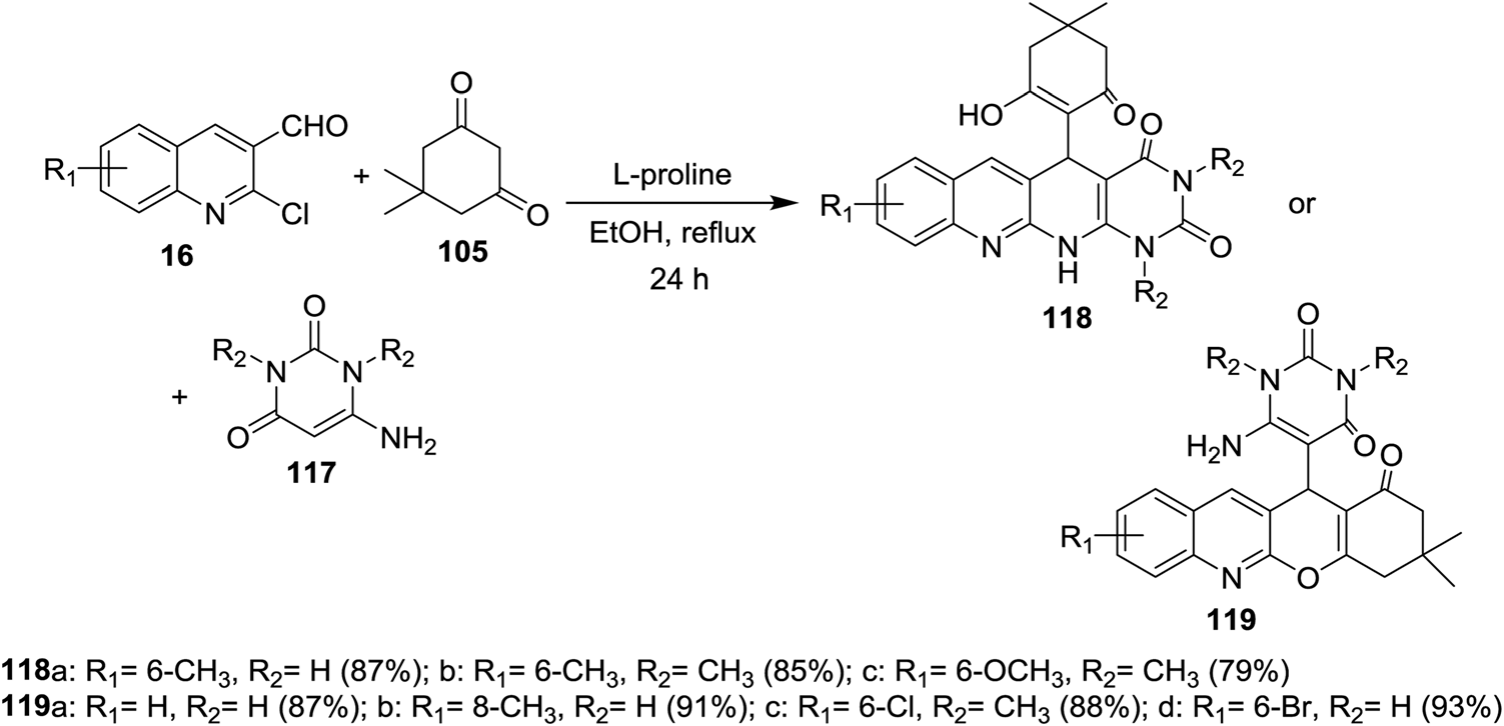
Synthesis of dihydrobenzo[*b*]pyrimido[1,8]naphthyridine-diones and 1*H*-chromenoquinolinyl-pyrimidine-diones.

Multicomponent reactions of substituted 2-chloro-3-formylquinolines 16 with 5,5-dimethylcyclohexane-1,3-dione (105) and 6-amino-pyrimidine-2,4(1*H*,3*H*)-diones 117 in refluxing ethanol containing l-proline yielded the desired dihydrobenzo[*b*]pyrimido[1,8]naphthyridine-2,4(1*H*,3*H*)-diones 118a–c and 1*H*-chromeno[2,3-*b*]quinolinyl-pyrimidine-2,4(1*H*,3*H*)-diones 119a–d (Scheme 37).^85^

**Scheme 38:**
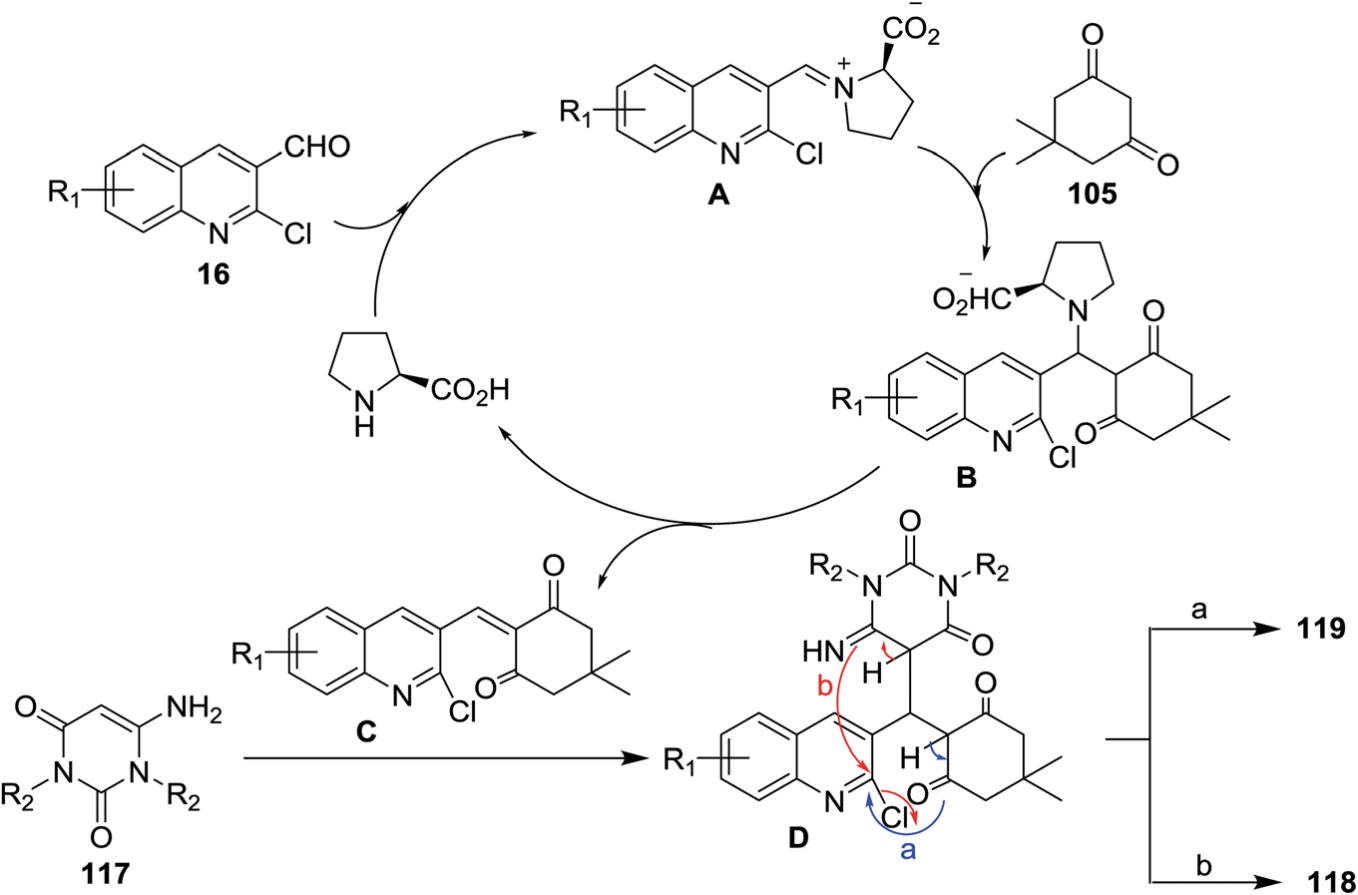
Mechanistic pathway for the synthesis of compounds 118 and 119.

The mechanistic pathways for the formation of compounds 118a–c and 119a–d were explained as shown in Scheme 38. The iminium ion (intermediate A) was formed through a reversible reaction of l-proline with aldehyde 16. The higher reactivity of intermediate A facilitate the Knoevenagel condensation with 105, through intermediate B, and after l-proline elimination, the intermediate C might be formed. The addition of aminouracil derivatives 117 furnish the intermediate D, which followed intramolecular cyclization initiated from N or O atoms to give compounds 118a–c and 119a–d, respectively.^85^

### 4.8. Synthesis of pyrazolopyrano-quinolinyl-pyrimidines

**Scheme 39:**
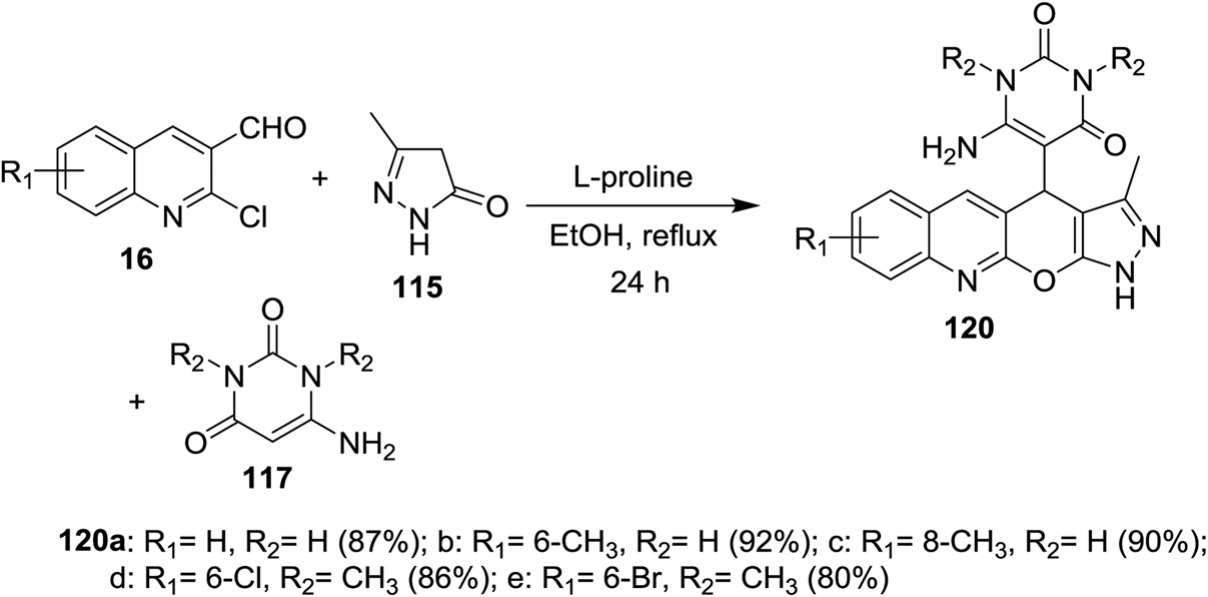
Synthesis of dihydropyrazolo[4′,3′:5,6]pyrano[2,3-*b*]quinolinyl-pyrimidine-diones.

Multicomponent one-pot reactions of substituted 2-chloro-3-formylquinolines 16 with 6-amino-pyrimidine-2,4(1*H*,3*H*)-diones 117 and 5-methyl-2,4-dihydro-3*H*-pyrazol-3-one (115) in refluxing ethanol catalyzed by l-proline gave the corresponding dihydropyrazolo[4′,3′:5,6]pyrano[2,3-*b*]quinolinyl-pyrimidine-2,4(1*H*,3*H*)-diones 120a–e (Scheme 39).^85^

### 4.9. Synthesis of oxazepinoquinolines

**Scheme 40:**
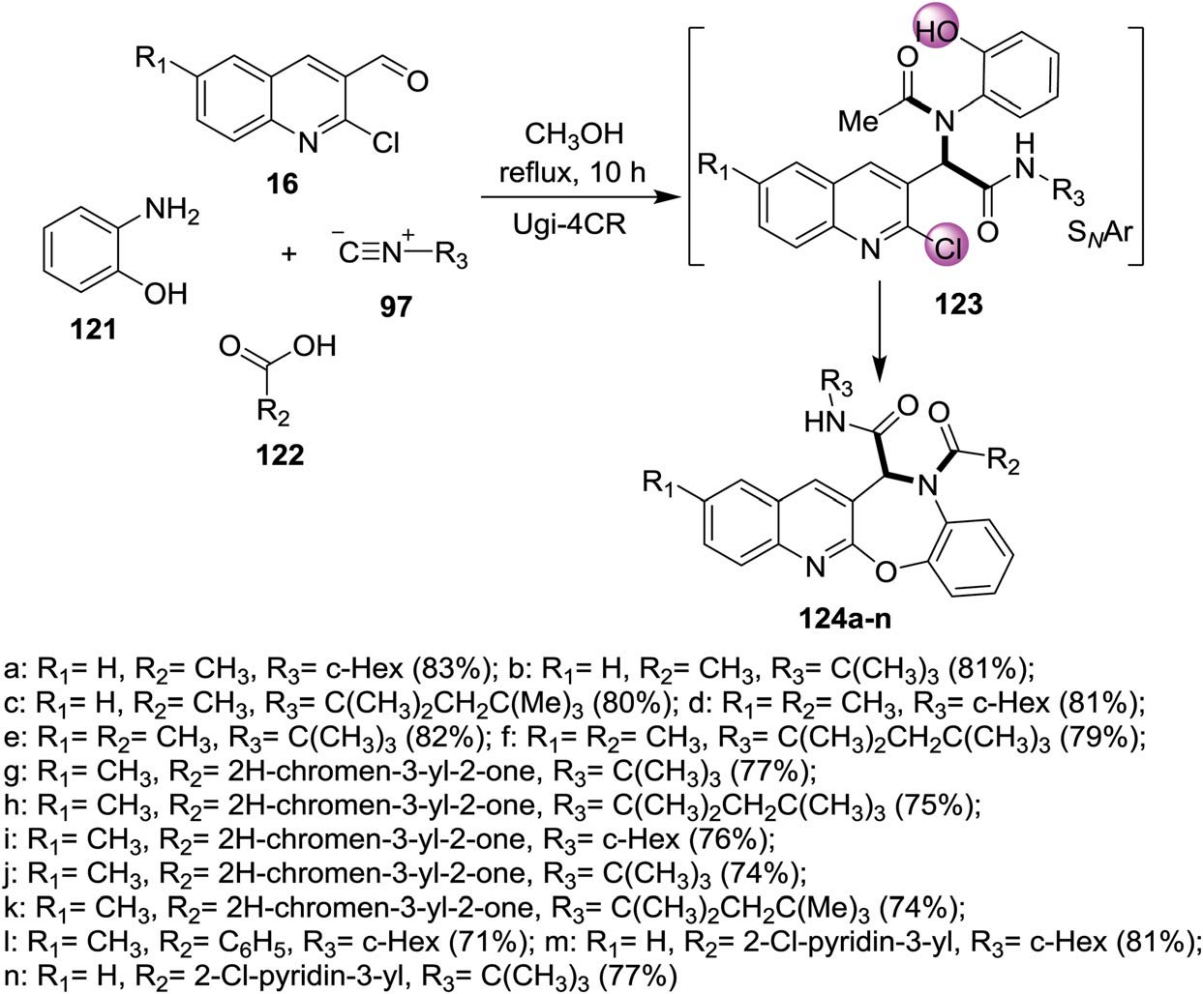
Synthesis of benzo[2,3][1,4]oxazepino[7,6-*b*]quinolines.

One-pot multicomponent reaction of quinolines 16 with 2-aminophenol (121), acids 122 and isocyanides 97 in refluxing methanol to give the corresponding benzo[2,3][1,4]oxazepino[7,6-*b*]quinolines 124a–n (Scheme 40). Ugi-4CR reaction conditions were used to prepare the investigated compounds 124a–n in (58–85%) yields in the absence of bases.^86^

## 5. Synthesis of binary heterocyclic systems

### 5.1. Synthesis of quinolinyl-azetidinones

**Scheme 41:**
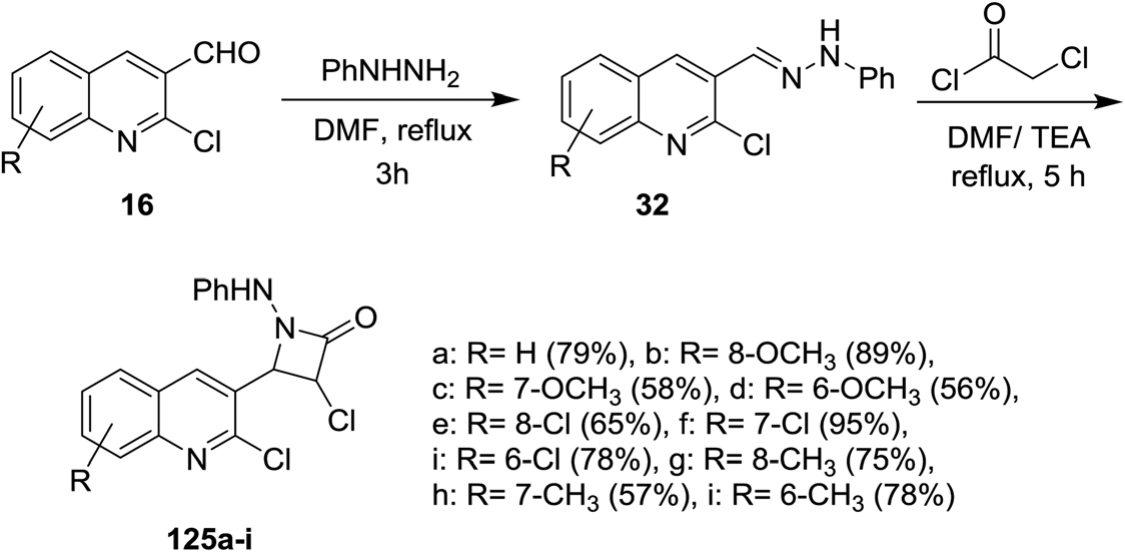
Synthesis of quinolin-3-yl-azetidin-2-ones.

Condensation of quinolines 16 with phenylhydrazine in refluxing DMF gave the respective Schiff bases 32. Cycloaddition reactions of 32 with chloroacetyl chloride in DMF containing triethylamine (TEA) yielded 3-chloro-4-(2-chloroquinolin-3-yl)-1-(phenylamino)azetidin-2-ones 125a–i in moderate to good yields (Scheme 41). The investigated compounds 125a–i exhibited good diuretic activity.^54^

### 5.2. Synthesis of quinolinyl-furan derivatives

**Scheme 42:**
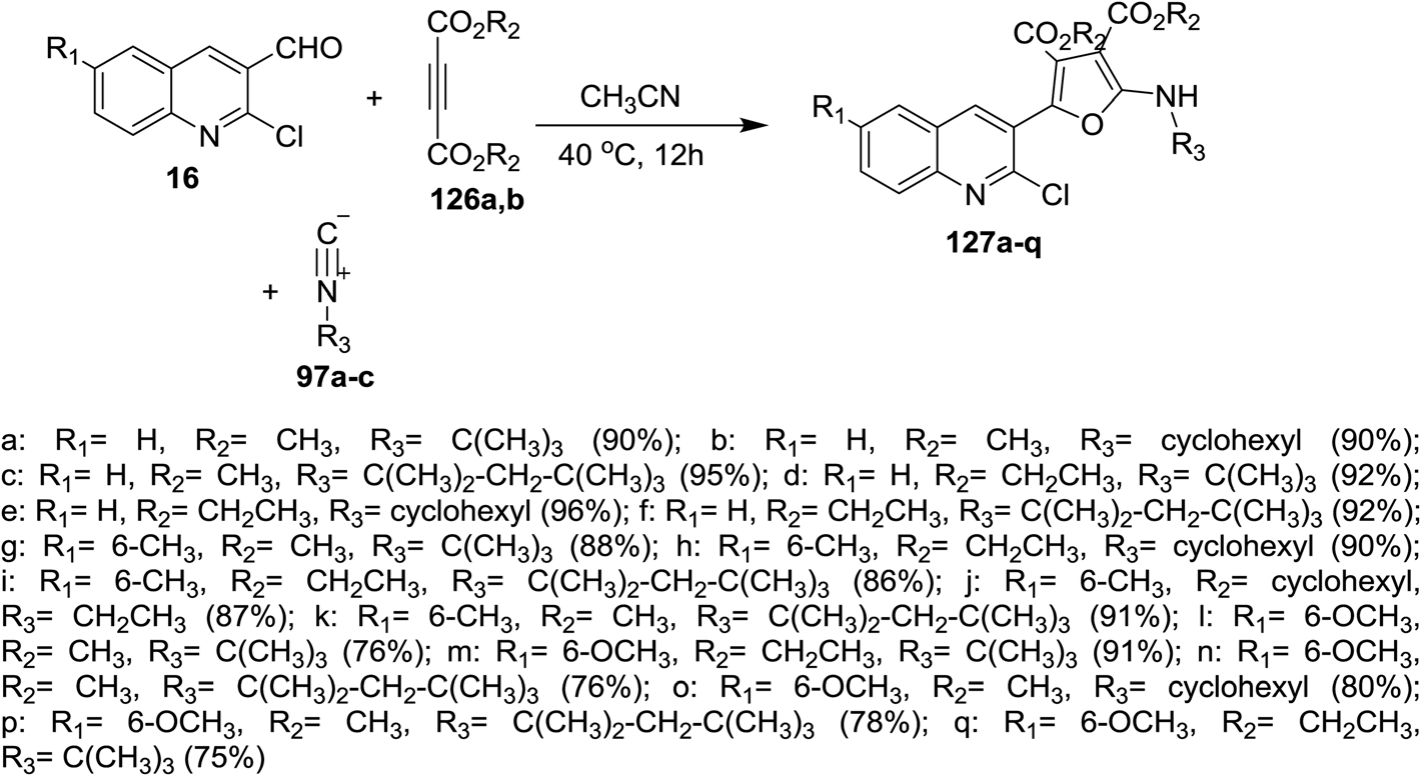
Synthesis of quinolinyl-furan-3,4-dicarboxylates.

One-pot multicomponent reactions of 16 with dialkyl but-2-ynedioates 126a,b, and isocyano alkanes 97a–c in acetonitrile gave quinolinyl-furan-3,4-dicarboxylates 127a–q in good yields (Scheme 42). The reaction was proceeded by the nucleophilic attack of carbonium ion of isocyanides to C≡C followed by nucleophilic attack of the formed anion to formyl group of 16 to afford the target compounds 127a–q.^87^

### 5.3. Synthesis of quinolinyl-pyrazoles

**Scheme 43:**
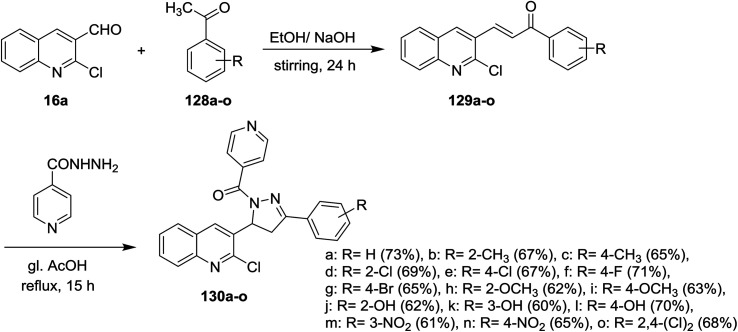
Synthesis of chloroquinolinyl-pyrazolyl-pyridines.

Claisen–Schmidt condensation of quinoline 16a with acetophenones 128a–o in ethanol containing sodium hydroxide as a base at room temperature gave the respective chalcone derivatives phenylpropenones 129a–o. Condensation of chalcones 129a–o with isonicotinohydrazide in refluxing glacial acetic acid afforded the corresponding chloroquinolinyl-pyrazolyl-pyridines 130a–o (Scheme 43). Compounds 130f, 130n, and 130o exhibited good antibacterial activity against *E. coli* at 50 μg mL^−1^ MIC, while at lower concentration (MIC = 12.5 μg ml^−1^), compound 130e have the highest inhibition against *E. coli*. Most of compounds 130a–o exhibited no to moderate toxicity against HeLa cells cultured at 100 μg mL^−1^.^88^

**Scheme 44:**
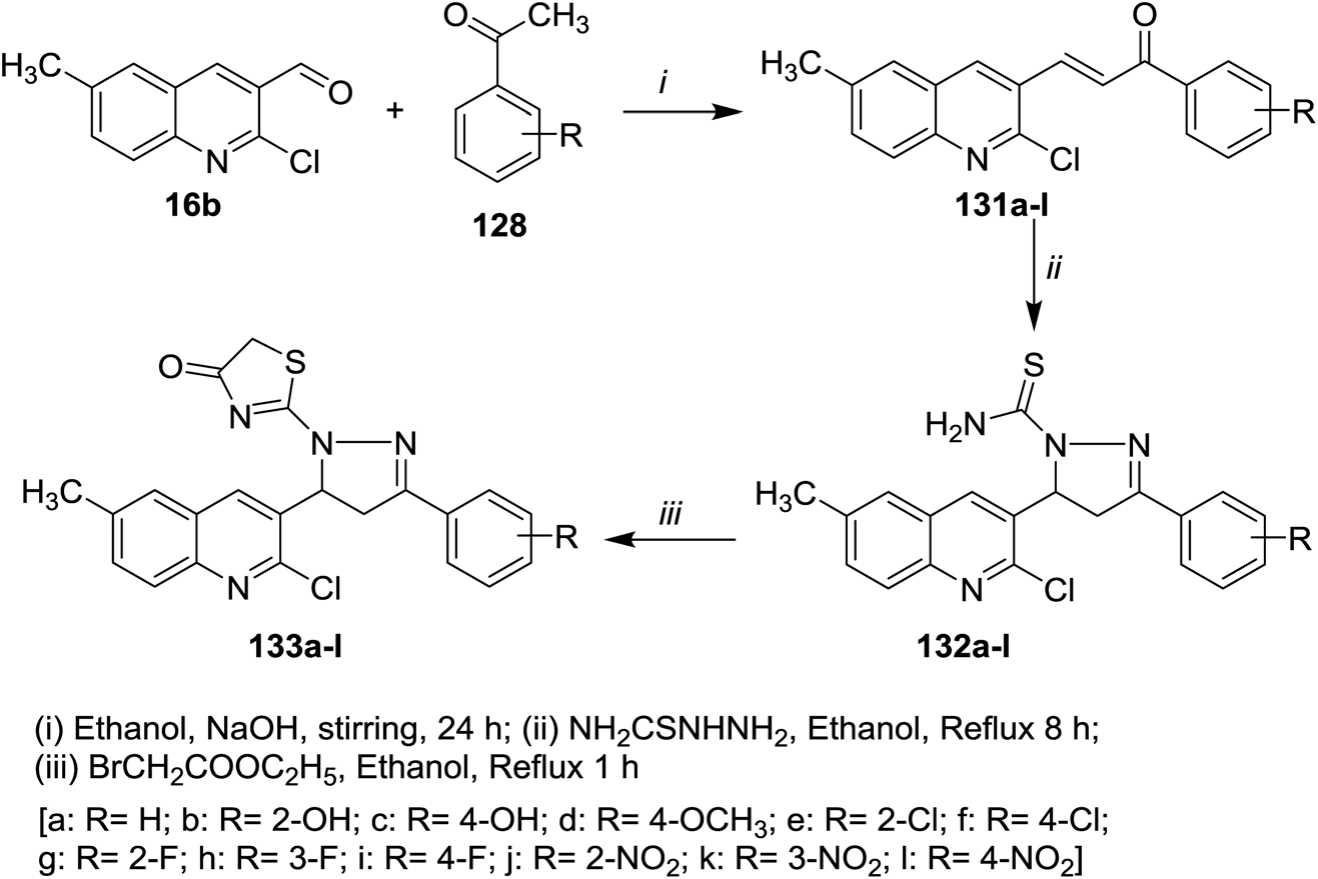
Synthesis of quinolin-3-yl-pyrazol-1-yl-thiazol-4(5*H*)-ones.

Furthermore, condensation of 2-chloro-3-formyl-6-methylquinoline (16b) with acetophenones 128 in ethanol containing sodium hydroxide gave the respective unsaturated ketones 131a–l. Cyclocondensation of 131a–l with hydrazinecarbothioamide by heating in ethanol afforded quinolinyl-pyrazole-1-carbothioamides 132a–l, respectively. Refluxing of 132a–l with ethyl bromoacetate gave the respective thiazol-4(5*H*)-ones 133a–l (Scheme 44).^89^

### 5.4. Synthesis of quinolinyl-oxazolidines

**Scheme 45:**
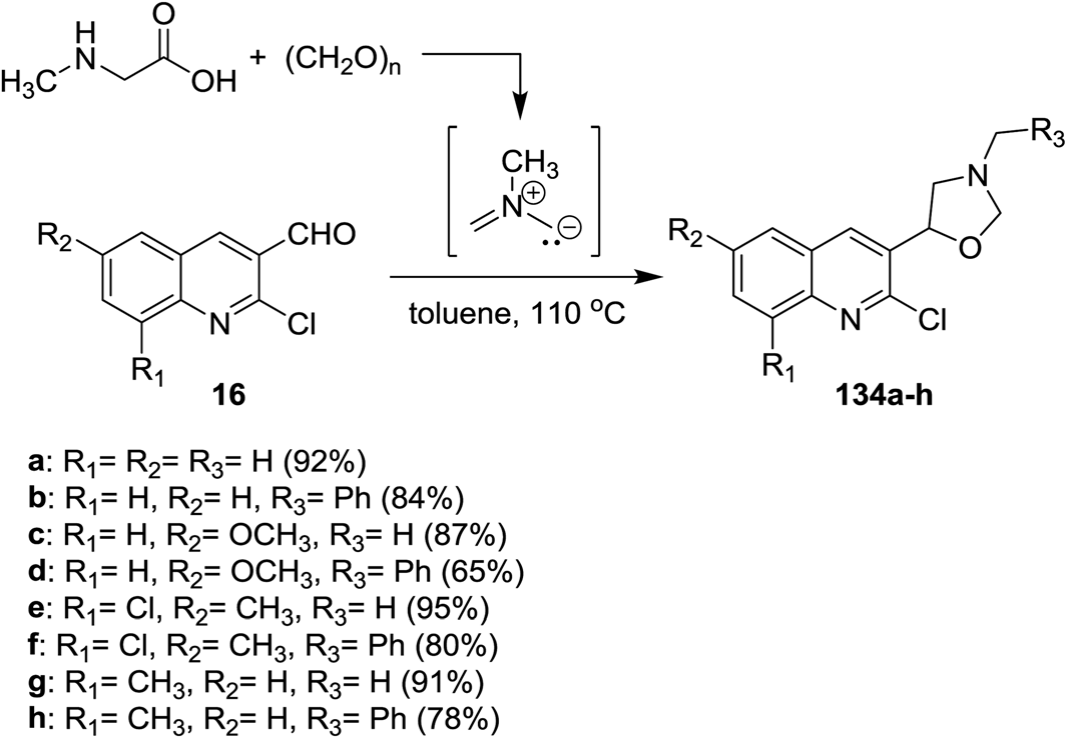
Synthesis of 2-chloroquinolinyl-oxazolidines.

Multicomponent reactions of quinolines 16 with methylglycine and formaldehyde in boiling toluene afforded the desired chloroquinolinyl-oxazolidine derivatives 134a–h in excellent yields (Scheme 45). The products were obtained through cycloaddition 1,3-dipolar reactions of the formed *N*-methyl-*N*-methylenemethanideaminium intermediate to quinolines 16.^52^

### 5.5. Synthesis of quinolinyl-thiazolidinones

**Scheme 46:**
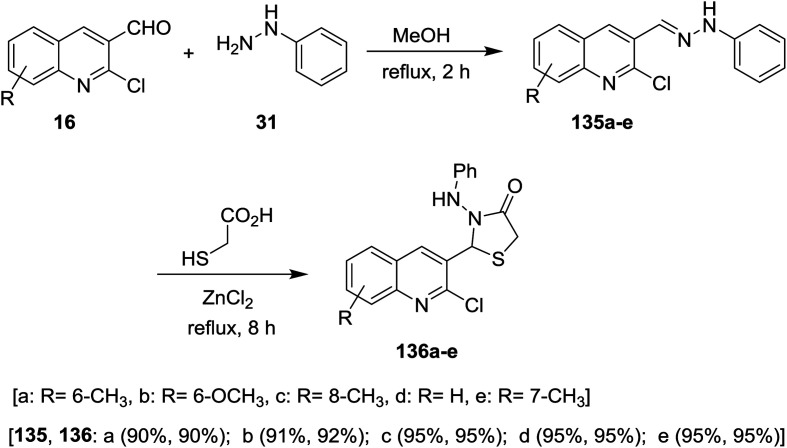
Synthesis of 2-(2-chloroquinolin-3-yl)-3-(phenylamino)thiazolidin-4-ones.

Condensation of quinolines 16 with phenyl hydrazine (31) in refluxing methanol gave the respective Schiff bases 135a–e. Treatment of 2-chloro-3-((2-phenylhydrazono) methyl) quinolines 135a–e with thioglycolic acid in boiling methanol containing a catalytic amount of zinc chloride afforded substituted 2-(2-chloroquinolin-3-yl)-3-(phenylamino)thiazolidin-4-ones 136a–e (Scheme 46). Compounds 136a–e have no antibacterial activity against *Salmonella typhi*, *E. coli*, *Bacillus subtilis* and *Staphylococcus aureus* microorganisms and no antifungal activity against *Aspergillus niger*. Compounds 136a–e exhibited good activity against *Penicillium chrysogenum*, while compounds 136a, 136c and 136e have activity towards *Fusarium moniliforme* and 136d against *Aspergillus flavus* microorganisms.^90^

**Scheme 47:**
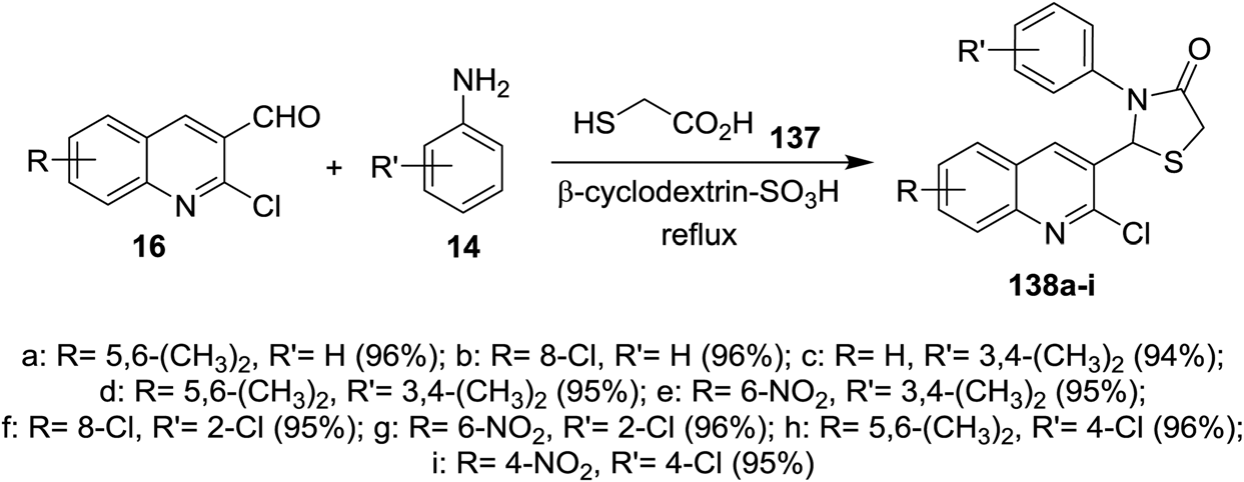
Synthesis of quinolinyl-thiazolidin-4-ones.

Solvent free multicomponent reactions of substituted 2-chloro-3-formylquinolines 16 with substituted anilines 14 and 2-mercaptoacetic acid (137) in the presence of catalytic β-cyclodextrin-SO_3_H furnished the respective quinolinyl-thiazolidinones 138a–i (Scheme 47). The best yields were obtained under the optimized conditions rather than using solvents such as methanol, ethanol, toluene, DMF or acetic acid and the yield% depends on the type of the catalyst.^91^

### 5.6. Synthesis of quinolinyl-1,3,4-oxadiazole

**Scheme 48:**
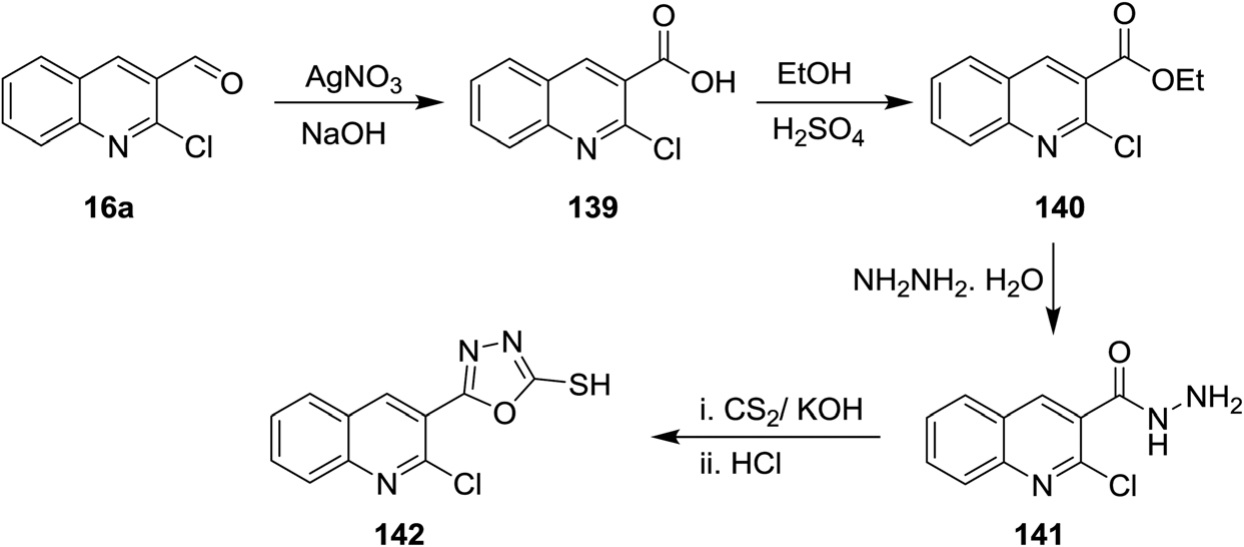
Synthesis of quinolin-3-yl-1,3,4-oxadiazole-2-thiol.

Oxidation of quinoline 16a with silver nitrite in the presence of sodium hydroxide gave the corresponding acid 139, which was esterified in ethanol containing sulfuric acid to yield the corresponding ester 140. Treatment of this ester 140 with hydrazine hydrate followed by reaction with carbon disulfide and hydrochloric acid gave 5-(2-chloroquinolin-3-yl)-1,3,4-oxadiazole-2-thiol (142) (Scheme 48).^92^

### 5.7. Synthesis of quinolinyl-pyranones and 1,2-dihydropyridone

**Scheme 49:**
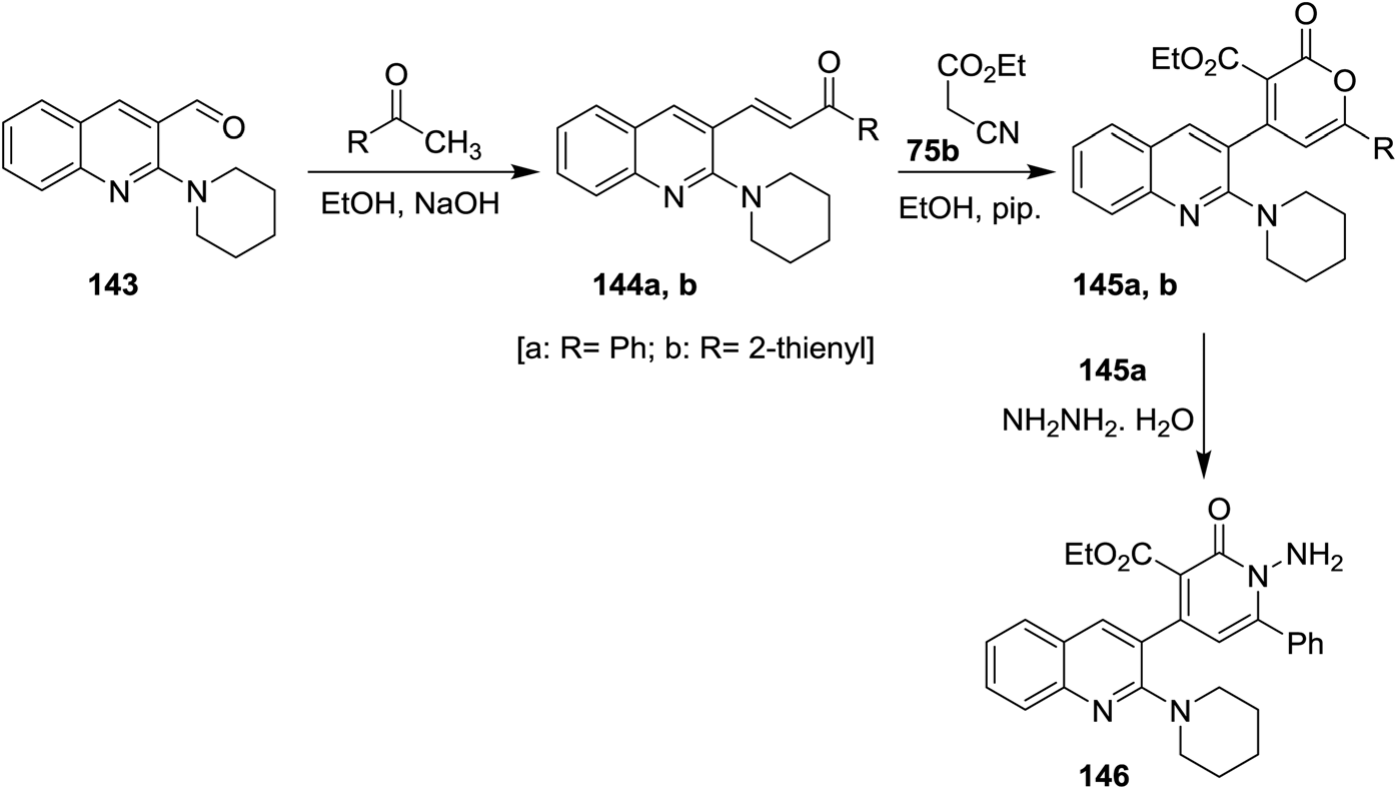
Synthesis of quinolinyl-1,2-dihydropyridine-3-carboxylate derivative.

Condensation of *N*-pyridylquinoline 143 with each of acetophenone and 1-(thiophen-2-yl)ethan-1-one in ethanol containing sodium hydroxide gave unsaturated ketones 144a,b, respectively. Cyclocondensation of 144a,b with ethyl cyanoacetate (75b) in ethanol containing piperidine gave the corresponding quinolinyl-pyranones 145a,b. Aminolysis of 145a with hydrazine hydrate gave quinolinyl-1,2-dihydropyridine-3-carboxylate 146. The reaction was preceded by nucleophilic addition at carbonyl group of lactone followed by condensation (Scheme 49).^92^

### 5.8. Synthesis of quinolinyl-quinazolines

**Scheme 50:**
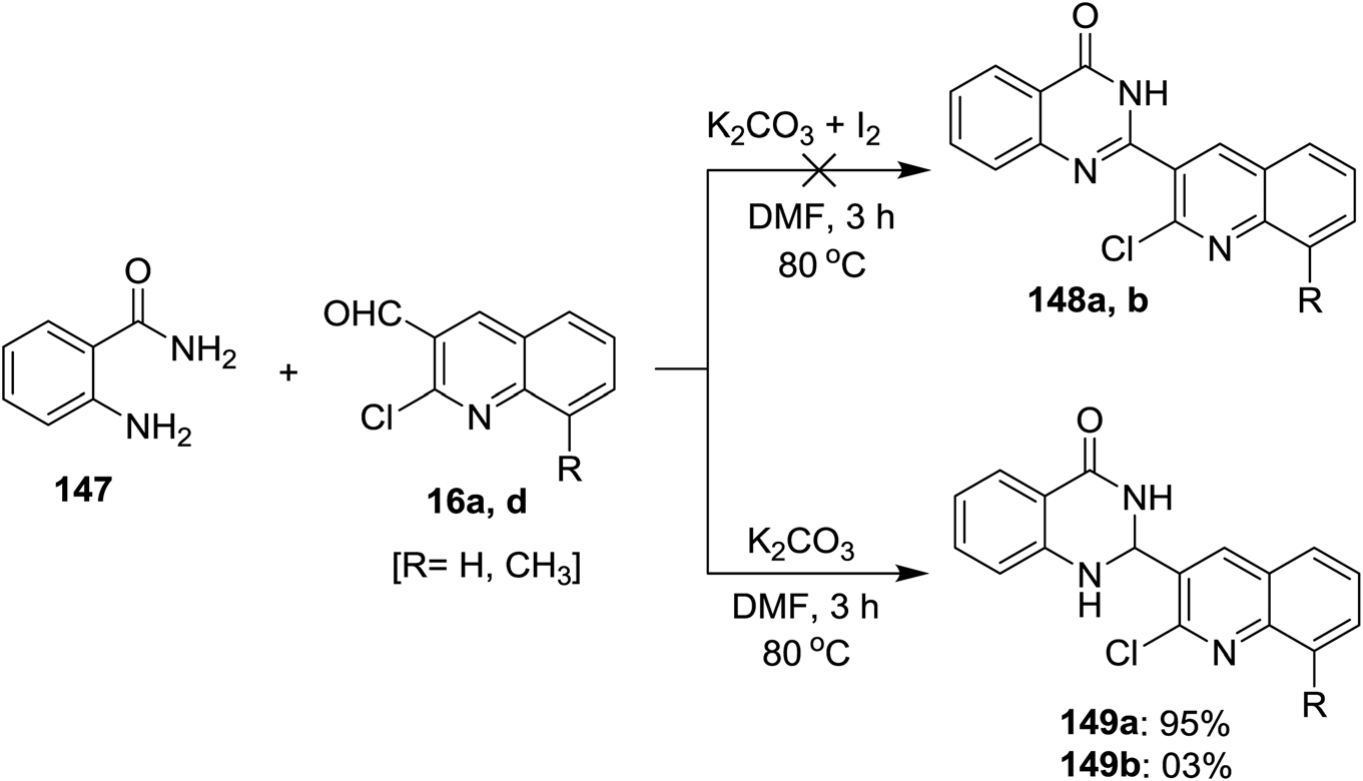
Synthesis of quinolinyl-quinazoline systems.

Treatment of each of quinolines 16a and 16d with 2-aminobenzamide (147) in hot DMF containing potassium carbonate gave 2-(2-chloro-quinolin-3-yl)-2,3-dihydroquinazolin-4(1*H*)-one 149a and its 8-methyl analog 149b was obtained in very low yield (3%). Unfortunately, the same reaction was carried out under the same conditions with the addition of iodine, but 2-(2-chloro-quinolin-3-yl)quinazolin-4(3*H*)-ones 148a and 148b were not obtained (Scheme 50).^93^

**Scheme 51:**
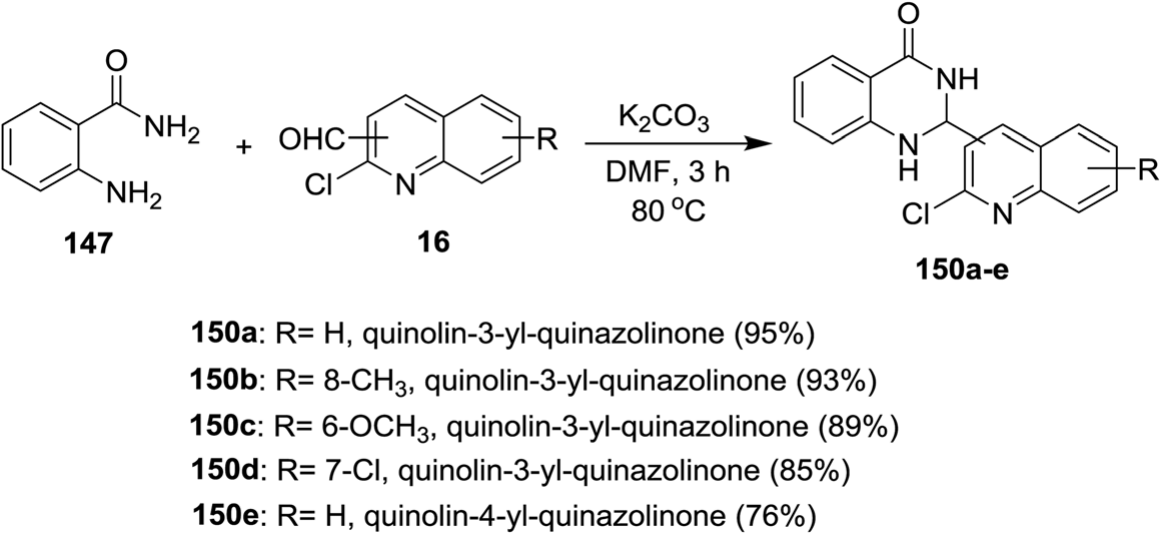
Synthesis of substituted-quinolinyl-quinazolines.

Similarly, a series of quinolinyl-quinazolinones 150a–e were prepared in good to excellent yields by heating the reactants 147 and 16 in DMF containing potassium carbonate without the addition of iodine (Scheme 51).^93^

**Scheme 52:**
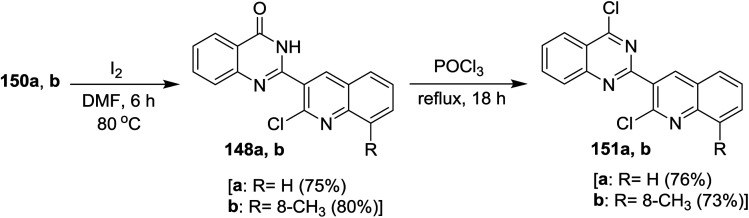
Synthesis of 4-chloro-2-(2-chloro-quinolin-3-yl)quinazolines.

Treatment of 2-(2-chloro-quinolin-3-yl)-2,3-dihydroquinazolin-4(1*H*)-ones 150a and 150b with iodine in hot DMF furnished 2-(2-chloro-quinolin-3-yl)quinazolin-4(3*H*)-ones 148a and 148b, respectively, through oxidation process using iodine or KMnO_4_. Refluxing of each of 148a and 148b with phosphorus oxychloride gave 4-chloro-2-(2-chloro-quinolin-3-yl) quinazolines 151a and 151b, respectively, in good yields (76 and 73%). It is noteworthy to mention that the product 151a was separated from resinous crude reaction products by recrystallization from ethanol (Scheme 52).^93^

### 5.9. Synthesis of quinolinyl-pyrimidines

**Scheme 53:**
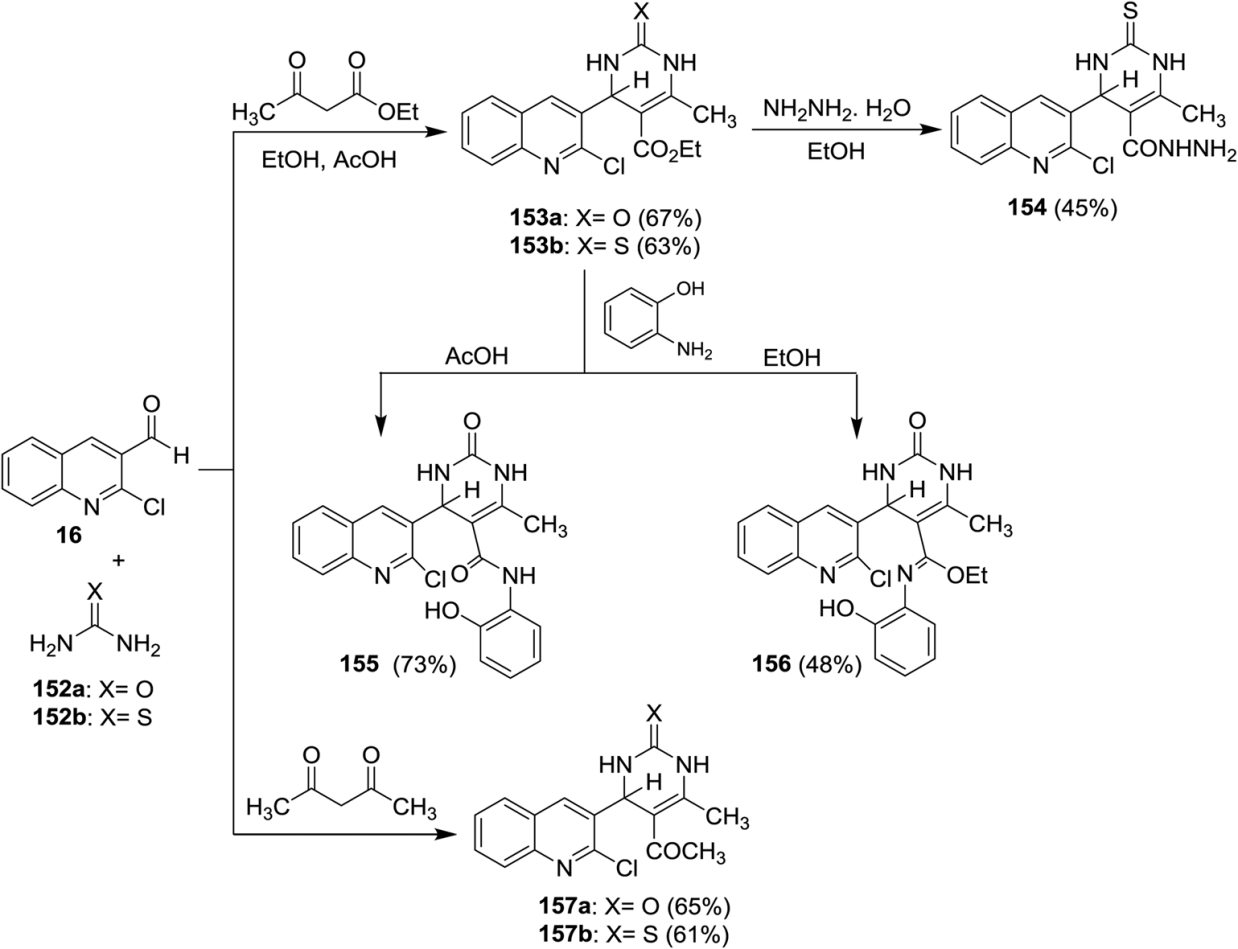
Synthesis of quinolinyl-pyrimidine systems.

Quinolinyl-tetrahydropyrimidine derivatives 153a,b, and 157a,b were synthesized from one-pot multicomponent reactions of quinoline 16 with urea (152a) or thiourea (152b) and ethyl acetoacetate or acetylacetone in refluxing ethanol containing acetic acid (1 mL). Treatment of 153b with hydrazine hydrate gave 4-(2-chloroquinolin-3-yl)-6-methyl-2-thioxo-1,2,3,4-tetrahydro-pyrimidine-5-carbohydrazide (154) through the elimination of ethanol molecule. The reaction of 153a with *o*-aminophenol in acetic acid gave 155, while the same reaction produced 156 by refluxing the reactants in ethanol instead of acetic acid for 6 h (Scheme 53).^62^

**Scheme 54:**
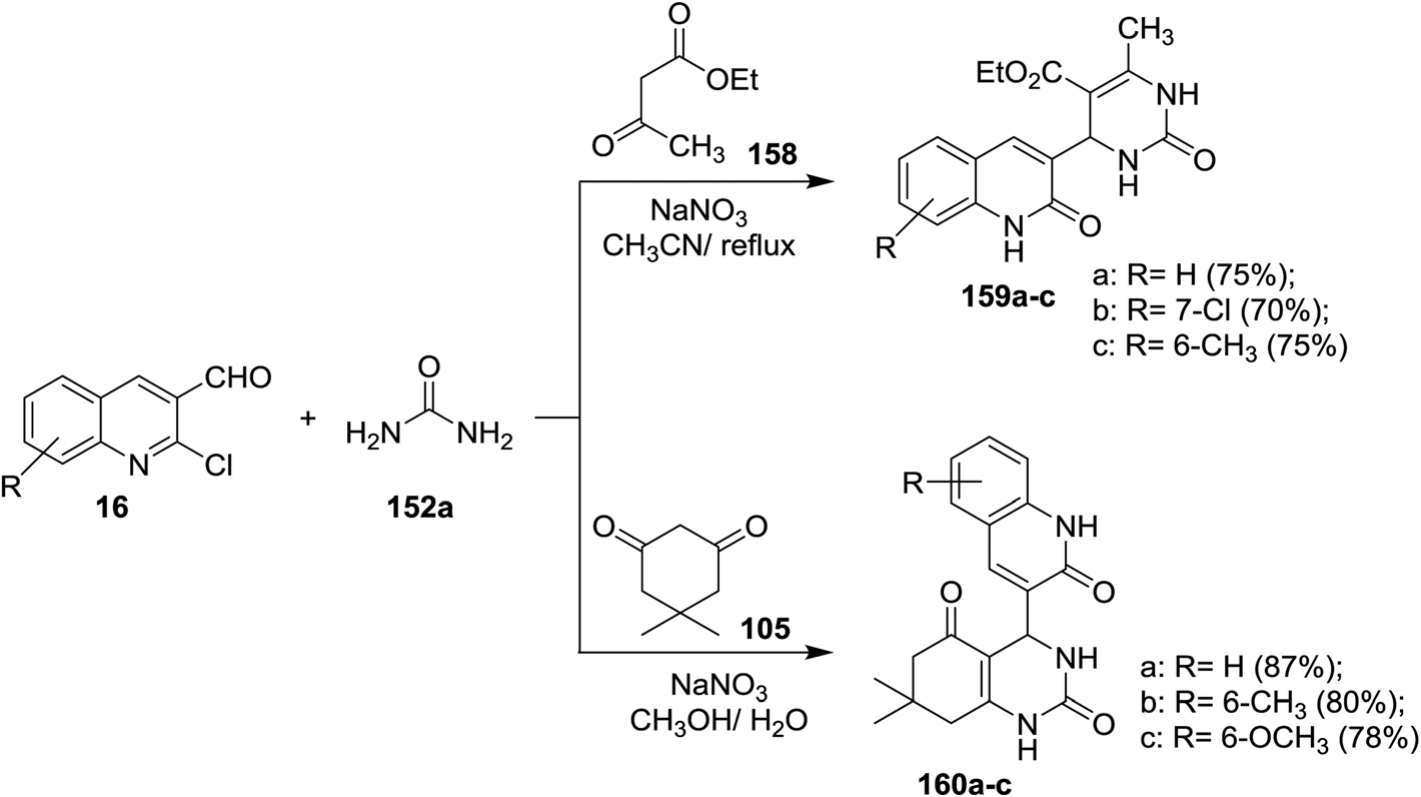
Synthesis of pyrimidinyl-quinoline systems.

Multicomponent reactions of substituted-2-chloro-3-formyl-quinolines 16 with urea (152a), and each of ethyl acetoacetate (158) and 5,5-dimethylcyclohexane-1,3-dione (105) in the presence of catalytic NaNO_3_ yielded tetrahydropyrimidine-5-carboxylates 159a–c and tetrahydro-quinazoline-2,5(1*H*,3*H*)-diones 160a–c, respectively. The presence of NaNO_3_ facilitate the condensation process of aldehydic carbonyl with amino group of urea followed by nucleophilic attack of the condensed nitrogen to active the hydrogen of ethyl acetoacetate (158) and condensation of another terminal amino group of urea with carbonyl group to produce the respective products 159a–c (Scheme 54).^94^

### 5.10. Synthesis of quinolinyl-pyranopyrazole

**Scheme 55:**
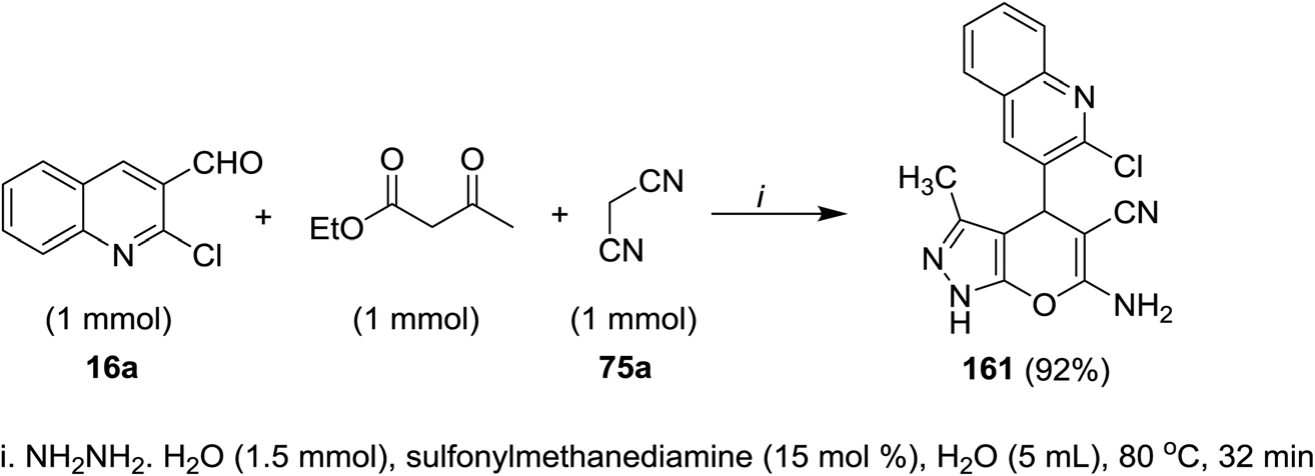
Multicomponent synthesis of quinolinyl-pyranopyrazole.

A multicomponent reaction of 2-chloro-3-formyl-quinoline (16a) with ethyl 3-oxobutanoate and malononitrile (75a) in water containing sulfonyl methane-diamine as a catalyst gave the respective quinolinyl-pyranopyrazole 161 (Scheme 55). The reaction was initiated by abstraction of active hydrogen from methylene group of malononitrile in the presence of a catalytic sulfonyl methanediamine followed by nucleophilic attack of the formed anion to formyl group of quinoline. Knoevenagel condensation of the produced alcohol produced the corresponding arylidene. Next, hydrazine hydrate reacted with ethyl acetoacetate to form pyrazole derivative which reacted with the formed arylidene in the previous step through Michael-type addition followed by intramolecular nucleophilic cyclization to give quinolinyl-pyranopyrazole 161.^95^

### 5.11. Synthesis of piperazinyl-quinolinyl-acridinones

**Scheme 56:**
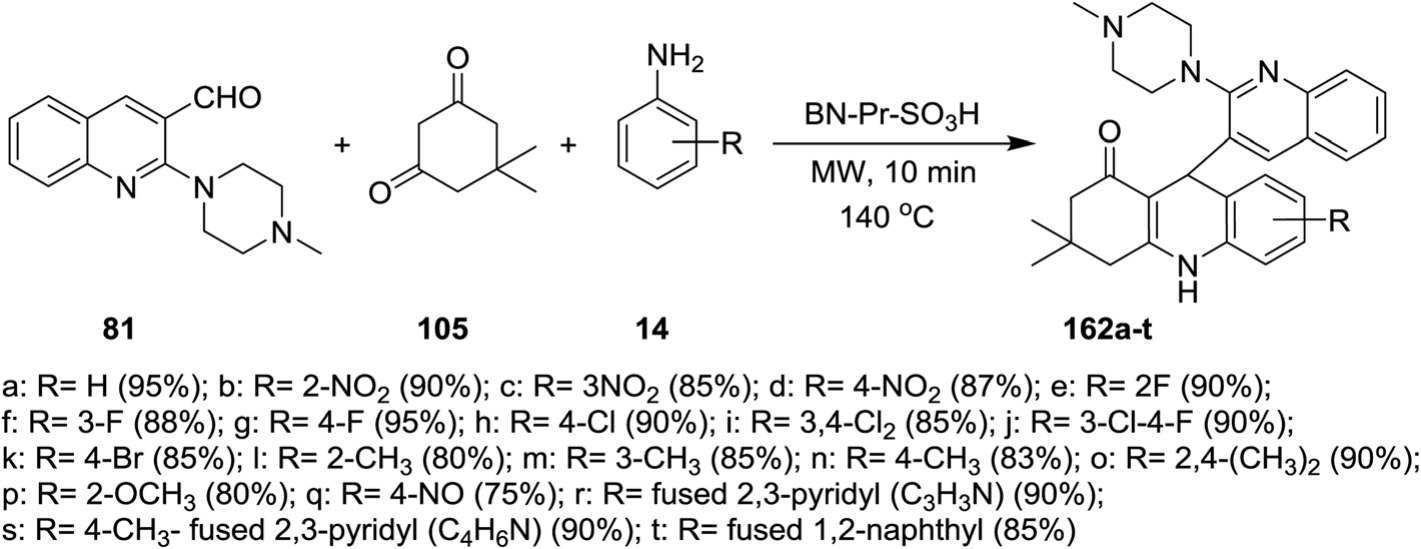
Synthesis of piperazinyl-quinolinyl-acridinone systems.

A multicomponent type reactions have been preferred than multistep synthetic reaction due to the high obtained yields%, low reaction time, high purified product, selectivity and formation of several bonds through a one-pot reactions.^96–98^ A multicomponent and solvent-free reaction of piperazinyl-quinoline 81 with 5,5-dimethylcyclohexane-1,3-dione (105) and substituted amines 14 under microwave conditions in the presence of catalytic amount of BN-Pr-SO_3_H afforded the respective piperazinyl-quinolinyl-acridinones 162a–t (Scheme 56). The reaction to prepare 3,3-dimethyl-9-(2-(4-methyl-piperazin-1-yl)quinolin-3-yl)-3,4,9,10-tetra-hydroacridin-1(2*H*)-ones 162a was carried out in different solvents such as ethanol, methanol, acrylonitrile and solvent-free conditions at room temperature or at 140 °C. It was found that the best yield was obtained (95%) following the conditions of solvent-free reaction at 140 °C after 10 minutes.^73^

**Scheme 57:**
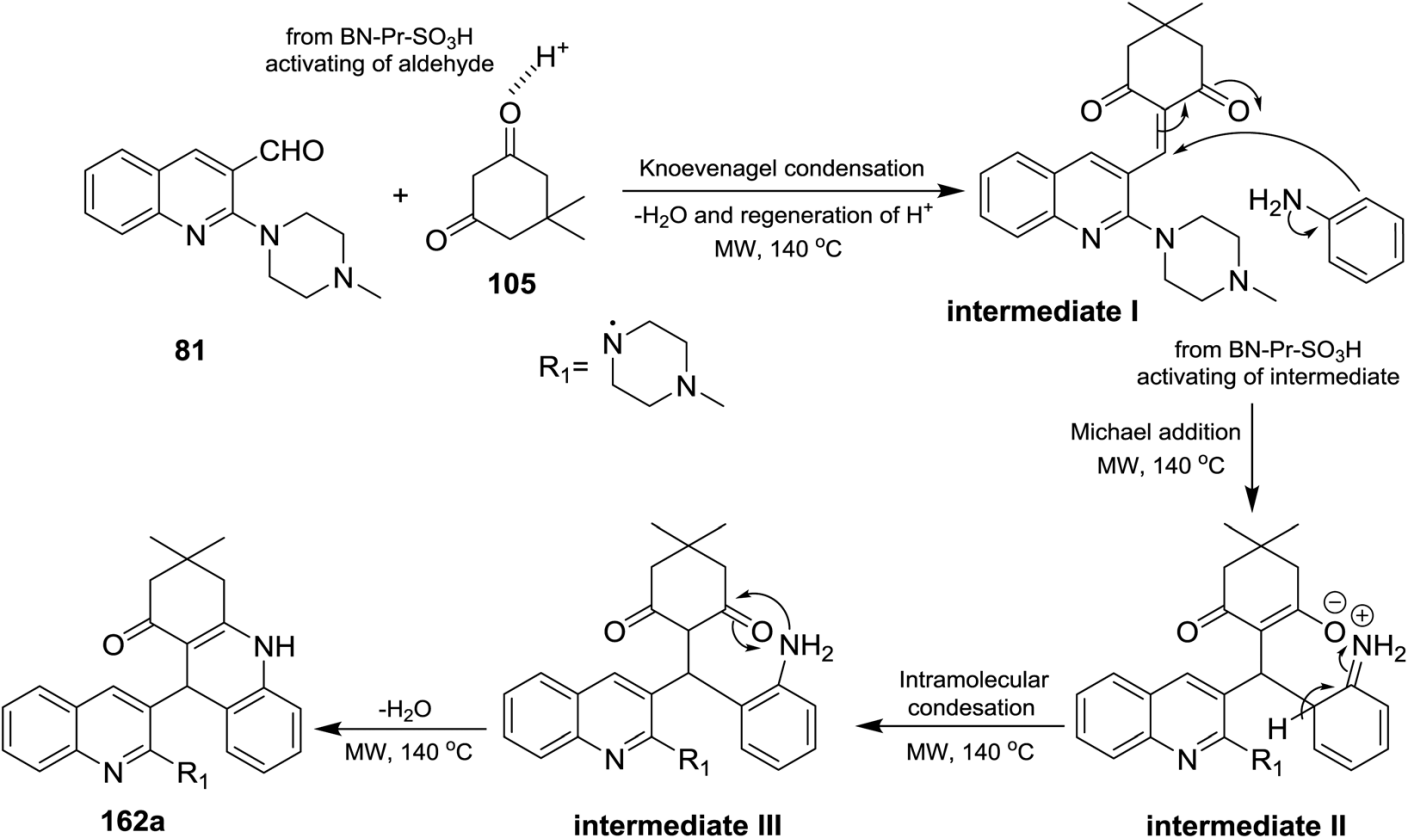
Mechanistic pathway for the synthesis of quinolinyl-tetrahydro-acridinone.

The reaction mechanism for the preparation of substituted 3,3-dimethyl-9-(2-(4-methylpiperazin-1-yl)quinolin-3-yl)-3,4,9,10-tetrahydro-acridin-1(2*H*)-ones 162a–t as shown in Scheme 57. The reaction was initiated through activating the aldehyde 81 using BN-Pr-SO_3_H followed by Knoevenagel condensation to form intermediate I. Next, Michael addition of the aromatic amines to the previously formed intermediate activated by BN-Pr-SO_3_H produced intermediate II which possess intramolecular condensation to yield 3,4,9,10-tetrahydroacridin-1(2*H*)-ones 162a–t after condensation of the formed intermediate III.^73^

### 5.12. Synthesis of quinolinyl-benzopyrido[1,4]diazepinones

**Scheme 58:**
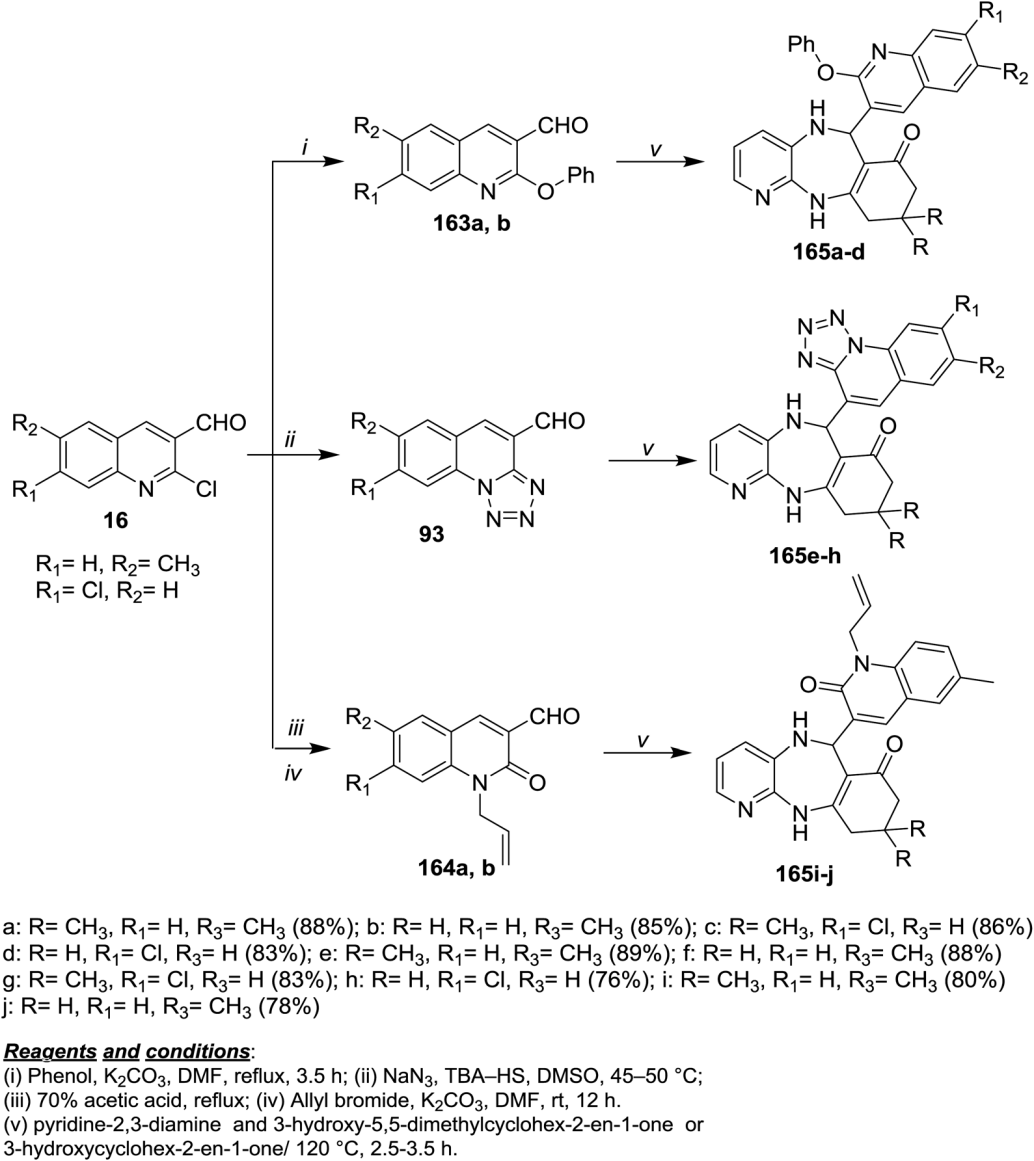
Synthesis of dihydroquinolinyl-benzopyrido[1,4]diazepinones and tetrazoloquinolinyl-benzopyrido[1,4]diazepinones.

Refluxing of each of 16a and 16b with phenol in DMF/K_2_CO_3_ afforded 2-phenoxyquinolines 163a and 163b, while the treatment with sodium azide in DMSO/TBA-HS gave tetrazolo[1,5-*a*]quinolines 93. Refluxing of 16 in acetic acid followed by treatment with allyl bromide in DMF/K_2_CO_3_ gave 164a,b. Multicomponent reactions of 93, 163 and 164a,b with pyridine-2,3-diamine and 3-hydroxy-5,5-dimethylcyclohex-2-en-1-one or 3-hydroxy-cyclohex-2-en-1-one gave quinolinyl-benzopyrido[1,4]diazepinones and tetrazoloquinolinyl-benzo-pyrido[1,4]diazepinones 165a–j in excellent yields (Scheme 58).^99^

### 5.13. Synthesis of piperazinyl-quinolinyl-benzothiazepines

**Scheme 59:**
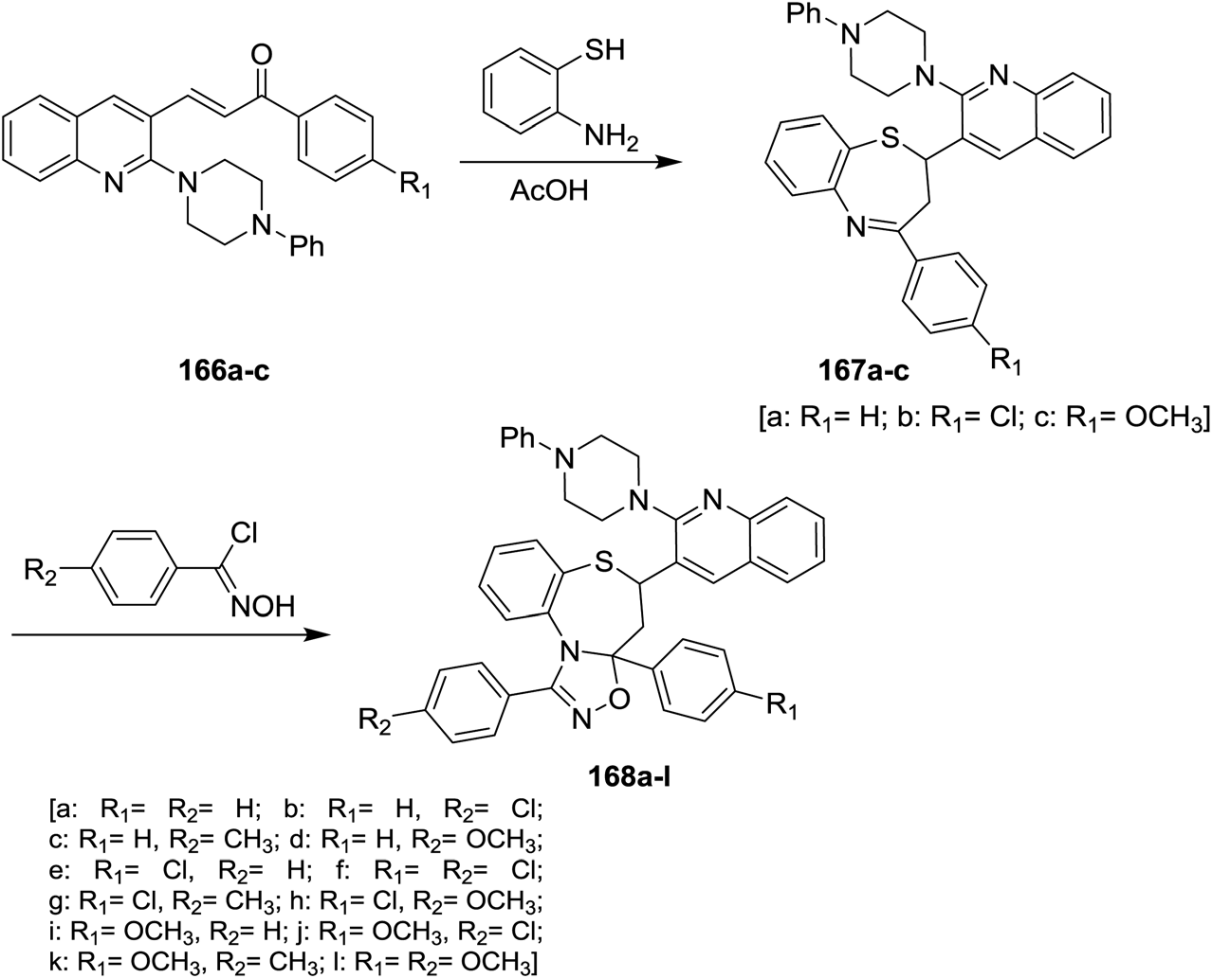
Synthesis of piperazinyl-quinolinyl-benzothiazepines.

Treatment of quinoline 16a with *N*-phenylpiperazine in hot DMF containing potassium carbonate yielded the desired 2-(4-phenyl-piperazin-1-yl)quinoline-3-carbaldehyde. Condensation of 2-(4-phenyl-piperazin-1-yl)quinoline-3-carbaldehyde with 4-substituted acetophenone in ethanol catalyzed by sodium hydroxide gave the respective unsaturated ketones 166a–c. Michael addition reaction of 166a–c with *o*-aminothiophenol in acetic acid yielded piperazinyl-quinolinyl-benzo-thiazepines 167a–c. Cycloaddition 1,3-dipolar reaction of quinolinyl-benzothiazepines 167a–c with *N*-hydroxy-4-substituted-benzimidoyl chloride in the presence of catalytic amount of triethylamine gave quinolinyl-benzo[*b*][1,2,4]oxadiazolo[4,5-*d*][1,4]thiazepines 168a–l (Scheme 59).^100^

## 6. Synthesis of heteryl-arylidenes

### 6.1. Synthesis of quinolidene-rhodanine system

**Scheme 60:**
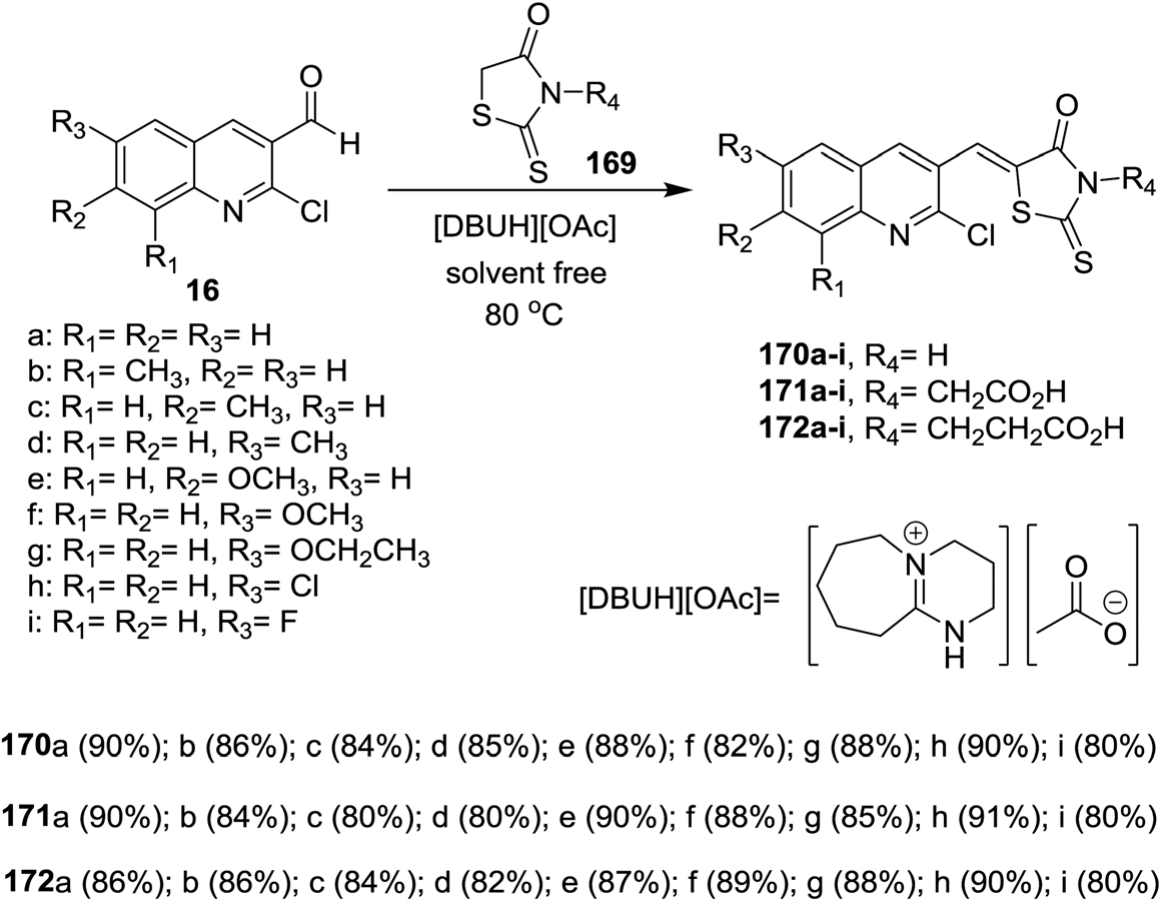
Synthesis of trisubstituted-quinolinyl-thioxo-thiazolidin-4-ones.

Solvent-free Knoevenagel condensation of quinolines 16 with *N*-substituted-2-thioxothiazolidin-4-ones (169) in the presence of ionic liquids of [Et_3_NH][HSO_4_] and [DBUH][OAc] afforded the respective trisubstituted-quinolinyl-thioxo-thiazolidin-4-ones 170–172a–i (Scheme 60). Quinolinyl-thioxo-thiazolidin-4-one 170g was more potent antifungal agent against *A. niger* (MIC = 25 μg mL^−1^) compared to miconazole, while, 170i is a good antifungal agent against *F. oxysporum*, *A. niger*, *C. neoformans* microorganisms (MIC = 25 μg mL^−1^) and *A. flavus* (MIC = 12.5 μg mL^−1^).^101^

### 6.2. Synthesis of quinoline-oxadiazoles

**Scheme 61:**
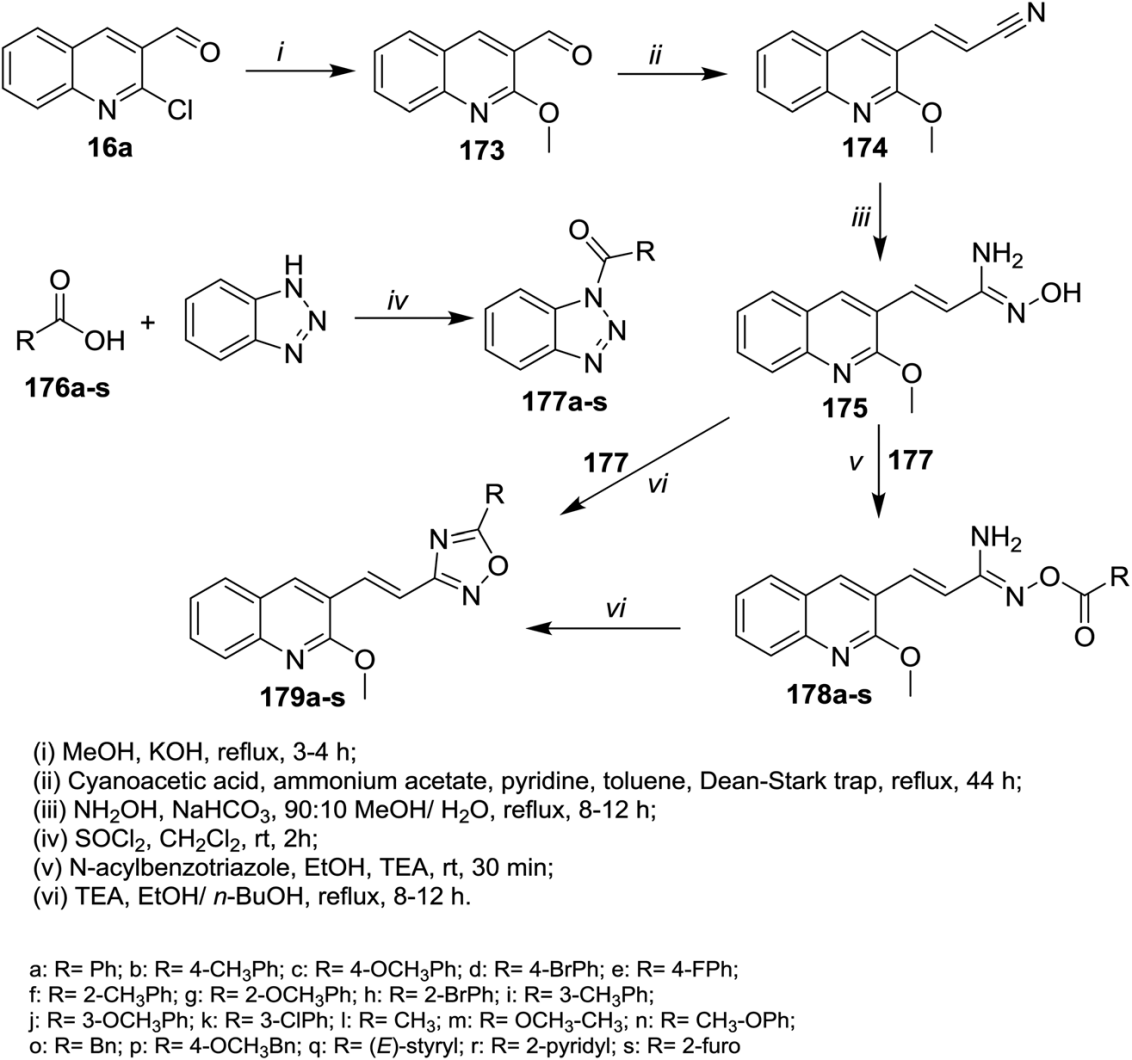
Synthetic routes to prepare quinolinyl-1,2,4-oxadiazoles.

Refluxing of quinoline 16a in methanol containing potassium hydroxide afforded 2-methoxyquinoline-3-carbaldehyde (173). Knoevenagel condensation of methoxy derivative 173 with cyanoacetic acid gave the corresponding arylidene derivative 174. Heating of 174 with *N*-acylbenzotriazole hydroxylamine hydrochloride in a mixture of methanol/water (9 : 1) containing sodium bicarbonate gave *N*′-hydroxy-3-(2-methoxyquinolin-3-yl)acryl imidamide (175). Treatment of 175 with *N*-acylbenzotriazole in ethanol catalyzed by triethylamine afforded the desired ester derivatives 178a–s. Intramolecular cyclization of 178a–s in boiling ethanol/*n*-butanol afforded quinolinyl-1,2,4-oxadiazoles 179a–s, respectively (Scheme 61).^102^

## 7. Concluding remarks

2-Chloroquinoline-3-carbaldehydes represent an extremely interesting class of organic compounds that can be exploited as precursors and building blocks for the synthesis of a wide range of heterocyclic systems and potent antibiotics for microbial and cancer treatment. In addition, quinoline moiety is the basic skeleton of many naturally occurring alkaloids and anticancer drugs. The recent publications describe the synthetic routes of 2-chloroquinoline-3-carbaldehydes following the Meth-Cohn synthesis using Vilsmeier reagent (DMF + POCl_3_ or PCl_5_) upon heating. 2-Oxo-1,2-dihydroquinoline-3-carbaldehydes are considered as reactive synthons in organic synthesis and obtained from the respective 2-chloro derivative by heating in acetic acid containing sodium acetate. 3-Formylquinolines are reactive towards condensation reactions with amines and hydrazines to produce Schiff bases. Nevertheless, in the last five years, the synthesis of fused quinoline heterocyclic systems was reported through a condensation reaction of formyl quinolines either with intramolecular cyclization or reaction with sodium azide or from multicomponent reaction with azidotrimethylsilane, isocyanides, and arylamines. Eventually, reactions with active methylene-containing compounds tend to yield the respective fused systems. The synthesis of binary heterocyclic systems has been attracted the researcher's interest in the last years due to the valuable biological and medicinal importance through the incorporation of other heterocycles into quinoline ring system. The present survey highlighted the recently cited research data in the literature on the chemistry of 2-chloroquinoline-3-carbaldehyde besides related analogs and their applications. It is certain that 2-chloroquinoline-3-carbaldehydes will continue to attract the attention of many researchers and that improvements in their synthesis, as well as novel transformations of these compounds, will be reported in the literature in the near future.

## Conflicts of interest

The author(s) confirm that this article content has no conflict of interest.

## Supplementary Material

## References

[cit1] Nammalwar B., Bunce R. A. (2014). Molecules.

[cit2] Mphahlele M. J., Adeloye A. O. (2013). Molecules.

[cit3] Al-Shaalan N. H. (2007). Molecules.

[cit4] Afzal O., Bawa S., Kumar S., Tonk R. K. (2012). Molbank.

[cit5] Jaware J., Borhade S. (2014). Indo Am. J. Pharm. Res..

[cit6] Michael J. P. (2004). Nat. Prod. Rep..

[cit7] Michael J. P. (2003). Nat. Prod. Rep..

[cit8] Alhaider A. A., Abdelkader M. A., Lien E. J. (1985). J. Med. Chem..

[cit9] Campbell S. F., Hardstone J. D., Palmer M. J. (1988). J. Med. Chem..

[cit10] Wu D. (2003). Tetrahedron.

[cit11] Subhashini N. J. P., Amanaganti J., Boddu L., Nagarjuna P. A. (2013). J. Chem. Pharm. Res..

[cit12] Gao W., Liu J., Jiang Y., Li Y. (2011). Beilstein J. Org. Chem..

[cit13] Keri R. S., Patil S. A. (2014). Biomed. Pharmacother..

[cit14] Vandekerckhove S., Herreweghe S. V., Willems J., Danneels B., Desmet T., de Kock C., Smith P. J., Chibale K., D'hooghe M. (2015). Eur. J. Med. Chem..

[cit15] Desai N. C., Kotadiya G. M., Trivedi A. R. (2014). Bioorg. Med. Chem. Lett..

[cit16] Vlahov R., Parushev J., Nickel P., Snatzke G. (1990). J. Pure Appl. Chem. Res..

[cit17] Srivastava A., Singh M. K., Singh R. M. (2005). Indian J. Chem..

[cit18] Pramilla S., Garg S. P., Nautiyal S. R. (1998). Indian J. Heterocycl. Chem..

[cit19] Vandekerckhove S., D'hooghe M. (2015). Bioorg. Med. Chem..

[cit20] Lyon M. A., Lawrence S., William D. J., Jackson Y. A. (1999). J. Chem. Soc., Perkin Trans. 1.

[cit21] Ahmed N., Brahmbhatt K. G., Sabde S., Mitra D., Singh I. P., Bhutani K. K. (2010). Bioorg. Med. Chem..

[cit22] Spanò V., Parrino B., Carbone A., Montalbano A., Salvador A., Brun P., Vedaldi D., Diana P., Cirrincione G., Barraja P. (2015). Eur. J. Med. Chem..

[cit23] El-Feky S. A., Abd El-Samii Z. K., Osman N. A., Lashine J., Kamel M. A., Thabet H. Kh. (2015). Bioorg. Chem..

[cit24] Kerry M. A., Boyd G. W., Mackay S. P., Meth-cohn O., Platt L. (1999). J. Chem. Soc., Perkin Trans. 1.

[cit25] Heinz H. P., Milhahn H. C., Eckart E. (1999). J. Med. Chem..

[cit26] Vivekanand B., Raj K. M., Mruthyunjayaswamy B. H. M. (2015). J. Mol. Struct..

[cit27] Maguire M. P., Sheets K. R., McVety K., Spada A. P., Zilberstein A. (1994). J. Med. Chem..

[cit28] Russo C. M., Adhikari A. A., Wallach D. R., Fernandes S., Balch A. N., Kerr W. G., Chisholm J. D. (2015). Bioorg. Med. Chem. Lett..

[cit29] Medapi B., Renuka J., Saxena S., Sridevi J. P., Medishetti R., Kulkarni P., Yogeeswari P., Sriram D. (2015). Bioorg. Med. Chem..

[cit30] Spicer J. A., Gamage S. A., Finlay G. J., Denny W. A. (1997). J. Med. Chem..

[cit31] Jacobs M. R., Appelbaum P. C. (2006). Expert Opin. Pharmacother..

[cit32] Yamakawa T., Mitsuyama J., Hayashi K. (2002). J. Antimicrob. Chemother..

[cit33] Kaur K., Jain M., Reddy R. P., Jain R. (2010). Eur. J. Med. Chem..

[cit34] Zhi C., Long Z., Manikowski A., Comstock J., Xu W., Brown N. C., Tarantino P. M., Holm K. A., Dix E. J., Wright G. E., Barnes M. H., Butler M. M., Foster K. A., Lamarr W. A., Bachand B., Bethell R., Cadilhac C., Charron S., Lamothe S., Motorina I., Storer R. (2006). J. Med. Chem..

[cit35] Shah N. M., Patel M. P., Patel R. G. (2012). J. Chem. Sci..

[cit36] Wall M. E., Wani M. C., Cook C. E., Palmer K. H., McPhail A. T., Sim G. A. (1966). J. Am. Chem. Soc..

[cit37] Kingsbury W. D., Boehm J. C., Jakas D. R., Holden K. G., Hecht S. M., Gallagher G., Caranfa M. J., Mccabe F. L., Faucette L. F., Johnson R. K., Hertzberg R. P. (1991). J. Med. Chem..

[cit38] Ban H.-J., Oh I.-J., Kim K.-S., Ju J.-Y., Kwon Y.-S., Kim Y.-I., Lim S.-C., Kim Y.-C. (2009). Tuberc. Respir. Dis..

[cit39] Kawato Y., Aonuma M., Hirota Y., Kuga H., Sato K. (1991). Cancer Res..

[cit40] Lin L.-Z., Cordell G. A. (1989). Phytochemistry.

[cit41] Abdel-Wahab B. F., Khidre R. E. (2013). J. Chem..

[cit42] Abdel-Wahab B. F., Khidre R. E., Farahat A. A., El-Ahl A.-A. S. (2012). ARKIVOC.

[cit43] Zoorob H. H., Hamama W. S. (1986). Pharmazie.

[cit44] Hamama W. S., Hassanien A. E., El-Fedawy M. G., Zoorob H. H. (2015). J. Heterocycl.
Chem..

[cit45] Hamama W. S., Hassanien A. E., Zoorob H. H. (2014). Synth. Commun..

[cit46] Hamama W. S., Hassanien A. E., El-Fedawy M. G., Zoorob H. H. (2017). J. Heterocycl. Chem..

[cit47] Hamama W. S., Hassanien A. E., El-Fedawy M. G., Zoorob H. H. (2016). J. Heterocycl. Chem..

[cit48] Meth-Cohn O. (1993). Heterocycles.

[cit49] Meth-Cohn O., Narine B., Tarnowski B. A. (1981). J. Chem. Soc., Perkin Trans. 1.

[cit50] Romero A. H. (2016). Synth. Commun..

[cit51] Tekale A. S., Mukhedker S. S., Shaikh S. A. L. (2015). Int. J. Chem. Stud..

[cit52] Toth J. (2006). Synth. Commun..

[cit53] Meth-Cohn O., Narine B. (1978). Tetrahedron Lett..

[cit54] Nayak G., Shrivastava B., Singhai A. K. (2016). Int. J. Curr. Pharm. Res..

[cit55] Zaheer Z., Khan F. A. K., Sangshetti J. N., Patil R. H., Lohar K. S. (2016). Bioorg. Med. Chem. Lett..

[cit56] Jia W., Liu Y., Li W., Liu Y., Zhang D., Zhang P., Gong P. (2009). Bioorg. Med. Chem..

[cit57] Aneesa F., Rajanna K. C., Reddy K. R., Ali M. M., Kumar Y. A. (2015). Synth. React. Inorg., Met.-Org., Nano-Met. Chem..

[cit58] Pericherla S., Mareddy J., Geetha R. D. P., Gollapudi P. V., Pal S. (2007). J. Braz. Chem. Soc..

[cit59] Desai N. C., Patel B. Y., Jadeja K. A., Dave B. P. (2017). Nov. Appro. Drug Des. Dev..

[cit60] Reddy L. V., Nakka M., Suman A., Ghosh S., Helliwell M., Mukkanti K., Mukherjee A. K., Pal S. (2011). J. Heterocycl. Chem..

[cit61] Nadaraj V., Selvi S. T. (2011). Int. Trans. J. Eng., Manage., Appl. Sci. Technol..

[cit62] Abd-El Maksoud M. A., Tawfik H. A., Maigali S. S., Soliman F. M., Moharam M. E., Dondeti M. F. (2016). Der Pharma Chem..

[cit63] Bontemps A., Mariaulea G., Desbène-Finck S., Helissey P., Giorgi-Renault S., Michelet V., Belmont P. (2016). Synthesis.

[cit64] Chavan H. V., Sirsat D. M., Mule Y. B. (2016). Iran. Chem. Commun..

[cit65] Bingul M., Tan O., Gardner C. R., Sutton S. K., Arndt G. M., Marshall G. M., Cheung B. B., Kumar N., Black D. S. C. (2016). Molecules.

[cit66] Sheejadevi K., Beulapriyanka G., Arul salomon T., Bhagyalakshmi S., Vijayakumar P., Suchitra M. (2013). Asian J. Pharm. Anal. Med. Chem..

[cit67] Li C.-R., Liu Z.-C., Wang B.-D., Li T.-R., Yang Z.-Y. (2015). Synth. Met..

[cit68] Bondock S., Gieman H. (2015). Res. Chem. Intermed..

[cit69] Raghavan S., Manogaran P., Narasimha K. K. G., Kuppusami B. K., Mariyappan P., Gopalakrishnan A., Venkatraman G. (2015). Bioorg. Med. Chem. Lett..

[cit70] Rao P. V., Kailas G. (2014). Int. J. Chem. Sci..

[cit71] Wang D.-W., Lin H.-Y., Cao R.-J., Chen T., Wu F.-X., Hao G.-F., Chen Q., Yang W.-C., Yang G.-F. (2015). J. Agric. Food Chem..

[cit72] Gohil J. D., Patel H. B., Patel M. P. (2016). Heterocycl. Lett..

[cit73] Murugesan A., Gengan R. M., Krishnan A. (2017). Mater. Chem. Phys..

[cit74] Muthumani P., Venkataraman S., Meera R., Nayak G., Chidambaranathan N., Devi P., Kameswari B. (2010). Der Pharma Chem..

[cit75] Mungra D. C., Kathrotiya H. G., Ladani N. K., Patel M. P., Patel R. G. (2012). Chin. Chem. Lett..

[cit76] Sangani C. B., Makawana J. A., Duan Y. T., Yin Y., Teraiya S. B., Thumar N. J., Zhu H. L. (2014). Bioorg. Med. Chem. Lett..

[cit77] Deshmukh A. R., Bhosle M. R., Khillare L. D., Dhumal S. T., Mishra A., Srivastava A. K., Mane R. A. (2017). Res. Chem. Intermed..

[cit78] Unnamatla M. V. B., Islas-Jácome A., Quezada-Soto A., Ramírez-López S. C., Flores-Alamo M., Gamez-Montano R. (2016). J. Org. Chem..

[cit79] Devi N., Rawal R. K., Singh V. (2015). Tetrahedron.

[cit80] Kishore K. G., Basavanag U. M. V., Islas-Jácome A., Gámez-Montaño R. (2015). Tetrahedron Lett..

[cit81] Kanani M. B., Patel M. P. (2014). RSC Adv..

[cit82] Rajkumar R., Dhivya P., Rajendran S. P. (2016). J. Chem., Biol. Phys. Sci. Sec. A.

[cit83] Fu L., Lin W., Hu M.-H., Liu X.-C., Huang Z.-B., Shi D.-Q. (2014). ACS Comb. Sci..

[cit84] Shiri M., Heydari M., Zadsirjan V. (2017). Tetrahedron.

[cit85] Shiri M., Pourabed R., Zadsirjan V., Sodagar E. (2016). Tetrahedron Lett..

[cit86] Ghandi M., Zarezadeh N., Abbasi A. (2016). Mol. Diversity.

[cit87] Ghandi M., Zarezadeh N. (2013). Tetrahedron.

[cit88] Desai N. C., Patel B. Y., Dave B. P. (2016). Med. Chem. Res..

[cit89] Desai N. C., Joshi V. V., Rajpara K. M. (2013). Med. Chem. Res..

[cit90] Shastri R. A. (2013). Chem. Sci. Trans..

[cit91] Shelke R. N., Pansare D. N., Pawar C. D., Deshmukh A. C., Pawar R. P., Bembalkar S. R. (2017). Res. Rev.: J. Chem..

[cit92] Radini I. A. M., Elsheikh T. M. Y., El-Telbani E. M., Khidre R. E. (2016). Molecules.

[cit93] Derabli C., Boulcina R., Kirsch G., Debache A. (2017). Tetrahedron.

[cit94] Boumoud B., Mennana I., Boumoud T., Mosset P., Debache A. (2013). Res. J. Pharm., Biol. Chem. Sci..

[cit95] Vekariya R. H., Patel K. D., Patel H. D. (2016). Res. Chem. Intermed..

[cit96] (b) Bienaymé H. and Zhu J., ed H. Bienaymé, Multicomponent Reactions, Wiley-VCH, Weinheim, 2005

[cit97] Ganem B. (2009). Acc. Chem. Res..

[cit98] Domling A., Ugi I. (2000). Angew. Chem., Int. Ed..

[cit99] Barad H. A., Sutariya T. R., Brahmbhatt G. C., Parmar N. J., Lagunes I., Padron J. M., Murumkar P., Sharma M. K., Yadav M. R. (2013). New J. Chem..

[cit100] Yang P., Lin H., Fei T., Liu F. (2017). J. Heterocycl. Chem..

[cit101] Subhedar D. D., Shaikh M. H., Shingate B. B., Nawale L., Sarkar D., Khedkar V. M., Khan F. A. K., Sangshetti J. N. (2016). Eur. J. Med. Chem..

[cit102] Jain P. P., Degani M. S., Raju A., Anantram A., Seervi M., Sathaye S., Ray M., Rajan M. G. R. (2016). Bioorg. Med. Chem. Lett..

